# METTL3 inhibition promotes radiosensitivity in hepatocellular carcinoma through regulation of SLC7A11 expression

**DOI:** 10.1038/s41419-024-07317-x

**Published:** 2025-01-11

**Authors:** Chen Zhang, Tianpeng Yang, Hanbin Chen, Xiaofeng Ding, Huajian Chen, Zhenzhen Liang, Yinlong Zhao, Shumei Ma, Xiaodong Liu

**Affiliations:** 1 School of Public Health, Wenzhou Medical University, the first affiliated hospital of Wenzhou Medical University, Wenzhou, 325035 China; 2 School of Public Health, Wenzhou Medical University, Wenzhou, 325035 China; 3 the first affiliated hospital of Wenzhou Medical University, Wenzhou, 325035 China; 4 School of Public Health, Wenzhou Medical University, School of Public Health, Xinxiang Medical University, Xinxiang, China; 5 Department of Nuclear Medicine, The Second Norman Bethune Hospital of Jilin University, Changchun, 130000 China; 6 School of Public Health, Wenzhou Medical University; South Zhejiang Institute of Radiation Medicine and Nuclear Technology, Wenzhou, 325035 China; 7 School of Public Health, Wenzhou Medical University; Key Laboratory of Watershed Science and Health of Zhejiang Province, Wenzhou Medical University, Wenzhou, 325035 China

**Keywords:** Radiotherapy, RNA modification

## Abstract

Radiotherapy is one of the main treatment modalities for advanced hepatocellular carcinoma (HCC). Ferroptosis has been shown to promote the radiosensitivity of HCC cells, but it remains unclear whether epigenetic regulations function in this process. In this study, we found that the overexpression of METTL3 was associated with poor prognosis. Knockdown of METTL3 promoted radiosensitivity of HCC by inducing ferroptosis. Mechanistically, METTL3 targeted adenine (+1795) on the SLC7A11 mRNA, and the m^6^A reader IGF2BP2 promoted SLC7A11 mRNA stability by recognizing and binding to the m^6^A site. Additionally, METTL3 decreased the ubiquitination of SLC7A11 protein through the m^6^A/YTHDF2/SOCS2 axis. Furthermore, in vivo studies showed that HCC models with low METTL3/IGF2BP2 expression have higher radiosensitivity. In conclusion, our study suggests that METTL3 regulates the stability of SLC7A11 mRNA in an m^6^A/IGF2BP2-dependent manner and the ubiquitination of SLC7A11 protein through the m^6^A/YTHDF2/SOCS2 pathway, both of which require the m^6^A methyltransferase activity of METTL3. METTL3 or IGF2BP2 may be promising targets for radiotherapy of HCC.

## Highlights

- METTL3 contributed to the resistance to radiation by regulating the expression of SLC7A11 in HCC cells.
- METTL3 maintained the mRNA stability of SLC7A11 in an m^6^A/IGF2BP2-dependent manner, via the binding of IGF2BP2 with the m6A site (+1795) of SLC7A11 mRNA.
- METTL3 decreased the ubiquitination of SLC7A11 protein through the m^6^A/YTHDF2/SOCS2 axis.
- Knockdown of METTL3/IGF2BP2 improved the radiosensitivity of HCC.

## Graphical abstract

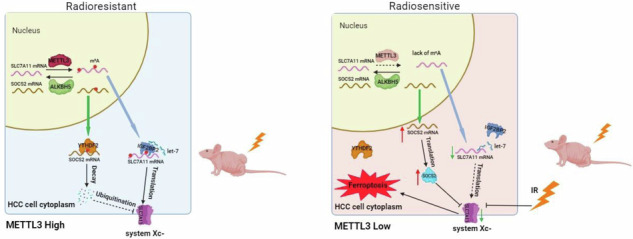

## Background

Primary liver cancer is currently the sixth most commonly diagnosed cancer and the third leading cause of cancer-related deaths worldwide, with approximately 906,000 new cases and 83,000 deaths each year. Hepatocellular carcinoma (HCC) accounts for the majority (75–85% of cases) of primary liver cancers [1]. Due to its insidious onset and lack of early specific symptoms, most patients were diagnosed at advanced stage and have not received appropriate treatment [2, 3]. With the development of local precision radiotherapy, such high-efficiency radiotherapy as stereotactic body radiation therapy (SBRT) has become one of the mainstream treatments for HCC. Localized precision radiotherapy can better protect normal liver tissues than conventional radiotherapy by optimizing the target dose and allowing the tumor to reach the therapeutic dose locally [4]. However, endogenous and treatment-induced radioresistance limits the efficacy of radiotherapy for HCC [5–7].

Contemporary patient selection for radiotherapy regimens is based on factors related to the patient (age, performance status or presence of comorbidity) and the tumor (such as size, stage and histological subtype); while the usage of information related to tumor biology is limited. Radiation affects multiple molecular pathways, including DNA damage, hypoxia, proliferation, stem cell phenotype and immune response, etc. Therefore, understanding the influence of these pathways on tumor response to radiotherapy and applying these mechanisms for the guidance of treatments, are of great clinical value. Recently high-throughput analyses and rapid profiling of RNA and DNA have been embedded in clinical laboratories. RNA-based signatures are among the most advanced tools currently available. For example, a ten-gene signature of cellular radiosensitivity is commercially available, and hypoxia-related signatures need further testing in clinical trials. Moreover, the development of DNA-based signatures is currently in progress, while some areas were still need further study such as the role of somatic mutations in DNA damage response and radiosensitivity [8]. Excitingly, our recent research established a multi-gene risk scoring model based on apoptosis, this model might predict the radiotherapy benefits of lung adenocarcinoma patients, importantly for those radioresistant patients classified by the model, we also selected effective adjuvant chemicals which would be used to make up the deficiencies of radiotherapy [9]. Therefore, improving or accurately predicting radiosensitivity of HCC is of great significance for patients’ treatment.

N6-methyladenosine (m^6^A) is the most common mRNA modification among various RNA epigenetic changes [10, 11]. Mechanistically, m^6^A is involved in almost all processes of RNA life span, including mRNA splicing, export, folding, degradation, and translation [12, 13]. By altering target gene expression, m^6^A influences multiple biological functions, such as tissue development, stemness maintenance and differentiation, DNA damage response, and metabolism [14–17]. The regulation of m^6^A involves three main components: methyltransferases (writers), demethylases (erasers), and RNA-binding proteins (readers) [18]. The m^6^A methyltransferase complex (MTC), which catalyzes the m^6^A modification, contains a core subunit, methyltransferase-like 3 (METTL3), and other accessory regulators, including METTL14, Wilms’ tumor 1-associating protein (WTAP), VIRMA, RBM15, and ZC3H13. Demethylases, such as fat mass and obesity-associated (FTO) and AlkB homology 5 (ALKBH5), reverse this effect. Additionally, RNA-binding proteins, including the YT521-B homology (YTH) domain family of proteins (YTHDF1/2/3 and YTHDC1/2) and insulin-like growth factor 2 mRNA binding proteins (IGF2BPP1/2/3), they could bind to m^6^A sites and transport target RNA to different destinations, or enhance RNA stability [19]. Recently, m^6^A formation have been reported to be essential for tumorigenesis and cancer progression in various cancer types, including HCC [20–22]. For instance, YTHDF2 modulates the m^6^A methylation of OCT4 mRNA to promote the cancer stem cell phenotype and cancer metastasis [23]; while METTL3 promotes HCC progression through an m^6^A-YTHDF2-dependent post-transcriptional silencing of SOCS2 [24].

Ferroptosis is a recently identified form of regulated cell death (RCD) driven by iron-dependent lipid peroxidation, which differs from apoptosis, necroptosis, and autophagy morphologically, genetically, and biochemically [25, 26]. The diverse array of ferroptosis defense systems, especially the system x_c_^-^-glutathione (GSH)-GPX4-dependent antioxidant defense, detoxifies lipid peroxides, thereby preventing their accumulation and maintaining cell survival [27, 28]. It was reported that ferroptosis and its regulatory proteins, such as glutathione peroxidase 4 (GPX4), and solute carrier family 7 member 11 (SLC7A11) played crucial roles in HCC [29, 30]. Nevertheless, it remains elusive whether epigenetic regulation, especially m^6^A methylation modifications, functions in the radiosensitivity of HCC.

Our previous study found that ferroptosis promotes the radiosensitivity of HCC cells [31]. Herein, we found that in HCC, METTL3 was correlated with ferroptosis and radiosensitivity, and further studies showed that METTL3 could directly regulate the transcription of the ferroptosis regulator SLC7A11 through m^6^A/IGF2BP2, and indirectly regulate the stability of SLC7A11 protein through m^6^A/YTHDF2/SOCS2. In HCC cells with METTL3 expression, IR mediated ferroptosis resistance, the underlying mechanism included the METTL3-dependent modulation of SLC7A11. In HCC cells without METTL3 expression, i.e., knockdown of METTL3 could lead to radiosensitization and mediate radiation-induced ferroptosis in HCC. Targeting METTL3 or IGF2BP2 may represent a promising therapeutic strategy, and the analysis of METTL3 and IGF2BP2 expression could be a potential signature for predicting the sensitivity of clinical radiotherapy in HCC.

## Materials and methods

### Cell lines and cell culture

HEK293T and Huh-7 cells were purchased from the Cell Resource Center, Peking Union Medical College (which is the headquarter of National Infrastructure of Cell Line Resource, NSTI). HepG2 and MHCC-97H cells were purchased from the Shanghai Cell Bank of the Chinese Academy of Sciences, China. L-O2 cells were kindly provided by Dr. Yujuan Shan’s lab (Wenzhou Medical University, China), and Hep3B and HCCLM3 cells were from Prof. Gang Chen’s lab (The First Affiliated Hospital of Wenzhou Medical University, China). All cells were cultured at 37 °C in a humidified incubator with 5% CO_2_ in Dulbecco’s modified Eagle’s medium (DMEM) (Gibco, United States) or Roswell Park Memorial Institute (RPMI) 1640 (Gibco, United States) supplemented with 10% fetal bovine serum (FBS) (WISENT, China), 100 U/ml penicillin and 100 μg/ml streptomycin (Invitrogen). Cells were passaged every 2 days to maintain logarithmic growth. Cell lines were authenticated by short tandem repeats (STR) profiling and confirmed to be mycoplasma-free.

### Plasmids construction and establishment of stable-transfected cell models

Target fragments were inserted into vectors such as pcDNA 3.1 Flag or lentiviral vectors pLVX-puro-HA and pLKO.1, followed by DNA sequencing for verification. Together with pMD2G and psPAX2 plasmids, recombinant lentiviral plasmids were transfected into HEK293T cells, in which recombinant lentivirus was generated. Target cells were incubated in lentivirus supernatant supplemented with 10 μg/ml polybrene, then treated with puromycin or Blasticidin for 7 to 14 days. The total protein was then extracted for western blotting to confirm the establishment of cell model with overexpression or knock-down of specific gene phenotype.

### RNA preparation and PCR

Total RNAs of cells were extracted using TRIzol reagent (TAKARA, Japan), and then reverse transcribed into cDNA using the Prime-Script™ one step real-time polymerase chain reaction (RT-PCR) kit (TAKARA, Japan). Semi-quantitative RT-PCR was performed with 2× Taq PCR Master Mix (TAKARA, Japan). GAPDH was used as the internal control. RT-qPCR was performed on QuantStudio 3 (Applied Biosystems, United States) using SYBR Green Mix (TAKARA, Japan). All primers used in our study were listed in Supplementary Tables 1 and  2 (Supplementary Additional file 1).

### Protein extraction and immunoblotting

Cells were trypsinized, washed with phosphate buffer saline (PBS), and lysed in ice-cold RIPA lysis buffer. Equal amounts of protein were separated by 10~12% SDS-PAGE and transferred to polyvinylidene difluoride (PVDF) membranes (Millipore, United States). The membranes were blocked for 1~2 h at room temperature in TBST with 6% (w/v) non-fat milk and incubated overnight at 4 °C with primary antibodies. After incubation with secondary antibodies, proteins were visualized with the ECL detection system (Thermo Fisher Scientific). The following antibodies were used: β-actin (66009-1-Ig, Proteintech), METTL3 (#86132S, CST), IGF2BP2 (11601-1-AP, Proteintech), ALKBH5 (A22137, Abclonal), SLC7A11/xCT (#12691S, CST), GPX4 (#59735S, CST), FTH1 (#4393S, CST), CD71 (#13113S, CST), HO-1 (ab137744, Abcam), ACSL4 (22401-1-AP, Proteintech), Transferrin (#35293S, CST), FLAG (F1804, Sigma), IGF2BP3 (14642-1-AP, Proteintech), FTO (A3861, Abclonal), METTL14 (A8530, Abclonal).

### RNA-immunoprecipitation

Cells were collected in lysis buffer (10 mM Tris-HCl (PH 7.5), 100 mM NaCl, 0.5% NP-40, 1% Triton X-100) containing 1 table EDTA-free protease Inhibitor Cocktail (Roche), 5 mM DTT (Thermo Scientific^TM^) and 200 units/ml Rnase OUT(invitrogen). Then 10% of cell lysate was taken as ‘input’ and the remains were divided into equal amounts and incubated overnight at 4 °C with anti-flag magnetic beads (Bimake) or IgG control. Subsequently, Lysis-antibody mixture was re-incubated with Protein A/G Dynabeads (B23201, Bimake) for 2 h at 4 °C and then washed 6 times with 1×NT-2 buffer (20 mM Tris-HCl (PH 7.5), 150 mM NaCl, 1 mM MgCl_2_, 0.05% NP-40). In contrast, lysis-beads mixture was washed directly for 6 times with 1×NT-2 buffer using DynaMag^TM^ -2 Magnet (Invitrogen). Next, washed samples were digested at 55 °C for 30 min with Proteinase K digestion buffer (1×NT-2 buffer containing 1% SDS, 1.2 mg/ml Proteinase K). Finally, the immunoprecipitated RNA was extracted using RNAiso Plus (Takara). Fold enrichment of the target region was determined after normalization to the input compared with IgG control.

### Methylated RNA immunoprecipitation sequencing and qPCR

Total RNA was isolated with TRIzol reagent (Invitrogen) and mRNA was enriched by the Dynabeads mRNA Purification Kit (Invitrogen). Next, RNA Fragmentation Reagents (AM8740, Invitrogen) were used to shear the RNA into about 100 nucleotide fragments. About 1/10 of the fragmented RNA was separated as input control for further RNA-seq. The remaining fragmented RNA was mixed with 50 μl of Dynabeads Protein A (Life Technologies) premixed with 16 μg of anti-m^6^A antibody at 4 °C overnight in IP buffer [150 mM NaCl, 10 mM Tris-HCl, and 0.1% NP-40 supplemented with ribonuclease (RNase) inhibitor and protein inhibitor]. The bead-antibody-RNA mix was washed with high-salt washing buffer twice, middle-salt washing buffer twice, and low-salt washing buffer twice separately. Following the last wash, 500 μl of TRIzol was added to the mix to extract the binding RNA. Both input and m^6^A IP samples were prepared for the next-generation sequencing by Novogene (China). Quantitative analysis of specific gene enrichment was calculated by qPCR, and the corresponding m^6^A enrichment in each sample was calculated by normalizing to the input.

### Colony formation assay

Cells were seeded in 6-well dishes and allowed to grow until visible colonies formed in a complete growth medium (10 days–2 weeks). The cell culture medium was refreshed every 3 days. Cell colonies were fixed with methanol, stained with crystal violet (Beyotime), and counted.

### Flow cytometric analysis

For assay for total cell death, cells were assessed by flow cytometry with trypan blue (Gibco, United States) following the manufacturer’s instruction. In brief, 72 h after IR cells were harvested and washed twice with cold PBS. Subsequently, cells were incubated with 200 μl of PBS containing 0.04% of trypan blue. Data were analyzed using NovoExpress Software on 10,000 events.

Lipid peroxidation was measured as previously described [32, 33], Fresh medium containing 5 μM BODIPY 581/591 C11 dye (Invitrogen, D3861) was added to the collected cell suspension for lipid peroxidation detection. After 30 min in the incubator (at 37 °C, 5% CO2), the cells were washed twice with PBS and finally resuspended in 200 μl PBS and analyzed by flow cytometry using the NovoExpress software (ACEA, USA) and per condition analyzed a minimum of 10,000 cells.

Reactive oxygen species generation was determined by flow cytometry with Dihydroethidium staining (DHE, D-1168). DHE could be oxidized by ROS into 2-hydroxyethidium (2-HE) (emission at 605 nm) and fluoresces red. The samples were collected and stained with 5 μM DHE and then incubated in the dark, inside the water bath at 37 °C for 15 min. The cell suspension was then transferred to a 5 ml FACS tube and analyzed on a flow cytometer within 10 min using Cell Quest software (BD Biosciences).

### Measurement of intracellular Fe^2+^ content

Cells were seeded at a density of 3000 cells per well in 96-well plates. According to the manufacturer’s instructions, intracellular Fe^2+^ ions was measured with a Ferro-orange kit (Dojindo, Molecular Technologies Inc., Shanghai, China). Briefly, cells were irradiated with 10 Gy of X-rays, and stained with 1 μM Ferro-orange in HBSS for 30 min at 37 °C at 24 h after IR. Then the fluorescence absorbance of the culture was immediately detected in an automatic microplate spectrophotometer (Synergy H1, BioTek Instruments, Vermont, USA) with an excitation wavelength of 543 nm and an emission wavelength of 580 nm.

### Transmission Electron Microscopy (TEM)

Cells were collected, fixed in TEM fixative at 4 °C, and washed with 0.1 M phosphate buffer (PB, pH 7.4). The samples were embedded in 1% agarose, post-fixed with 1% osmium tetroxide in PB for 2 hours, and dehydrated through a graded ethanol series followed by acetone. Resin infiltration was performed with acetone:EMBed 812 mixtures, progressing to pure resin, and samples were polymerized at 60 °C for 48 h. Ultrathin sections (60–80 nm) were cut, placed on copper grids, and stained with 2% uranyl acetate and 2.6% lead citrate. The grids were observed under a TEM, and images were captured.

### MDA content measurement

MDA in HCC cells were measured using a Malondialdehyde(MDA) Content Assay Kit (Solarbio life science, Beijing, BC0025), in accordance with the manufacturer’s instructions.

### In vivo xenograft experiments

All procedures for animal experiments were performed in accordance with the Guide for the Care and Use of Laboratory Animals (National Institutes of Health publication no. 80-23, revised 1996). All animal studies were approved by Wenzhou Medical University Committee for Experimental Animal Studies and Ethics.

Male athymic BALB/c-Nude mice, aged 5 weeks old, were purchased from GemPharmatech (Nanjing, China) and used at 6 weeks. The experimental animals were randomly divided into two groups with 10 mice in each group. Then, a total of 5× 10^6^ cells (shNC, shMETTL3/shBP2) were suspended in a mixture of 100 μL PBS and then injected subcutaneously into the hind leg. When the majority of tumors grew to about 100 mm^3^, each group of mice was further randomized into two groups of 5 mice each, the sham group and the IR group. IR groups were treated with 8 Gy X-ray for 3 consecutive days. Under the shield of lead plate, only tumor locations were exposed to IR. Tumor sizes were measured and calculated every 3 days. when the volume approached 1000 mm^3^, mice were sacrificed and tumors were excised and measured. Then the tumors were fixed in 4% tissue fixative for further examinations. Tumor volume was calculated as below: V (mm^3^)  =  width^2^ (mm^2^) × length (mm)/2.

### Statistical analysis

All values were expressed as mean ±S.D. Statistical analysis was performed by the unpaired Student’s *t* test. A probability value of *P* < 0.05 was considered statistically significant.

## Results

### METTL3 was high- expression in HCC and correlated with tumor proliferation

**Fig. 1:**
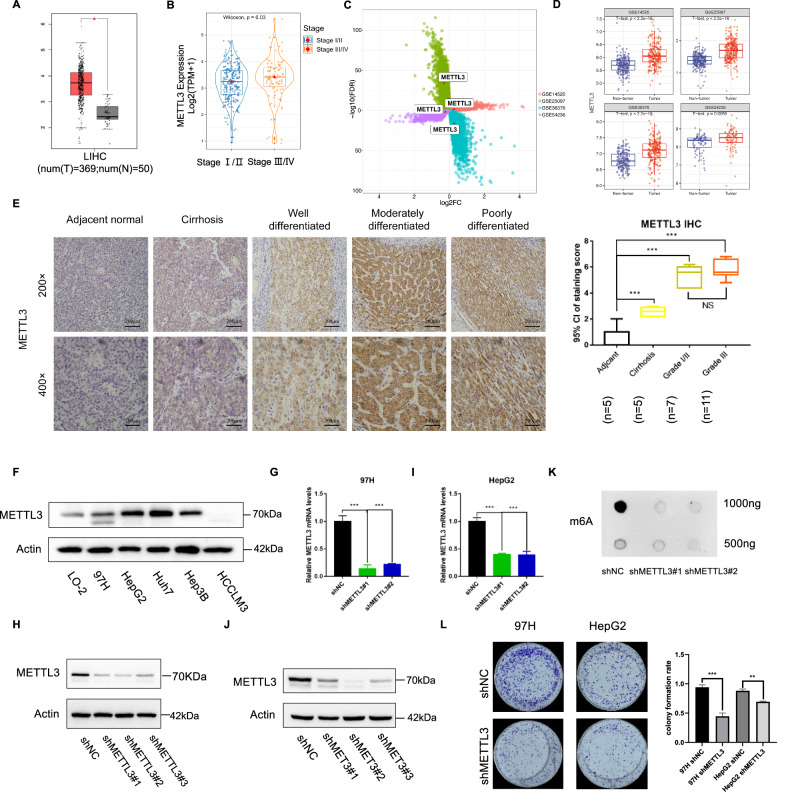
METTL3 was high- expressed in HCC and correlated with tumor proliferation. **A** TCGA database showed that METTL3 was elevated in LIHC tissue compared with normal tissue; **B** Analysis of METTL3 tumor stage-specific expression in TCGA LIHC database; **C**, **D** Analysis of METTL3 expression in four GEO datasets (GSE14520, GSE25097, GSE36376, GSE54236); **E** Representative immunohistochemistry images of the expression of METTL3 in HCC specimens compared with adjacent tissues, scale bar: 200 μm and association of METTL3 IHC-staining scores with tumor grades (I, II, and III). Data are plotted as the means of 95% confidence interval ±S.D; **F** Protein levels of METTL3 in L-O2, HepG2, MHCC-97H and Huh7 were detected by western blot; **G**, **I** Relative METTL3 mRNA levels after METTL3 knockdown in 97H (**G**) or HepG2 (**I**) were detected by qRT-PCR; **H**, **J** METTL3 protein levels after METTL3 knockdown in 97H (**H**) or HepG2 (**J**) were detected by western blot; **K** Overall m^6^A levels after knockdown of METTL3 in MHCC-97H cells were detected by m^6^A dot blot; **L** Cell proliferation abilities after knockdown of METTL3 in MHCC-97H and HepG2 cells were detected by colony formation assay; *P < 0.05, **P < 0.01, ***P < 0.001.

To investigate the role of METTL3 in HCC, we analyzed the METTL3 mRNA levels in LIHC from the TCGA database on the GEPIA platform (http://gepia.cancer-pku.cn/) and found that HCC tissues were with high expression of METTL3 (Fig. 1A). Furthermore, we analyzed the expression of METTL3 in diverse pathological stages of HCC and found that its expression was significantly higher in stage III and IV HCC compared to stage I and II (Fig. 1B). We also analyzed the METTL3 mRNA levels in several GEO datasets (Fig. 1C, D, GSE14520, GSE25097, GSE36376, GSE54236) and found that METTL3 was highly expressed in HCC samples of these datasets. Notably, METTL3 expression was also studied in paraffin-embedded sections of human HCC and normal liver specimens using immunohistochemistry (IHC), and the results indicated high expression of METTL3 in tumor tissues (Fig. 1E). Interestingly, the expression of METTL3 was also significantly higher in cirrhotic tissues than in normal liver tissues, but lower than that in HCC tissues (Fig. 1E), suggesting that METTL3 may play a role in the process of hepatocarcinogenesis. We examined the protein expression of METTL3 in five HCC cell lines (MHCC-97H, HepG2, Huh7, Hep3B, and HCCLM3) as well as in a normal liver cell line, L-O2, using western blotting. The results indicated that METTL3 was significantly upregulated in HCC cells, except for HCCLM3 (Fig. 1F). In addition, we examined the expression of other key m^6^A writer and eraser proteins (METTL14, FTO, and ALKBH5) in these four cell lines and found that the expression of FTO was higher in HCC cells than in normal hepatocytes (Supplementary Fig. S1A). To gain insight into the functional role of METTL3 in RT of HCC, we knock down METTL3 in HepG2 and MHCC-97H cell lines. The pLKO.1 vector was used to construct three shRNAs targeting METTL3. We utilized a lentiviral three-plasmid packaging system to generate lentiviral particles carrying shRNAs, and then infected the cell lines and measured knockdown efficiency by qRT-PCR and Western blot (Fig. 1G–J). Depletion of METTL3 resulted in a substantial decrease in m^6^A abundance in total RNA as detected by m^6^A dot blot assay (Fig. 1K). Further, knockdown of METTL3 induced a significant reduction in the proliferative capacity of HCC cells (Fig. 1L). These findings suggest that targeting METTL3 may represent a promising therapeutic strategy for HCC treatment.

### Knockdown of METTL3 enhanced radiosensitivity of HCC cells by promoting ferroptosis

**Fig. 2:**
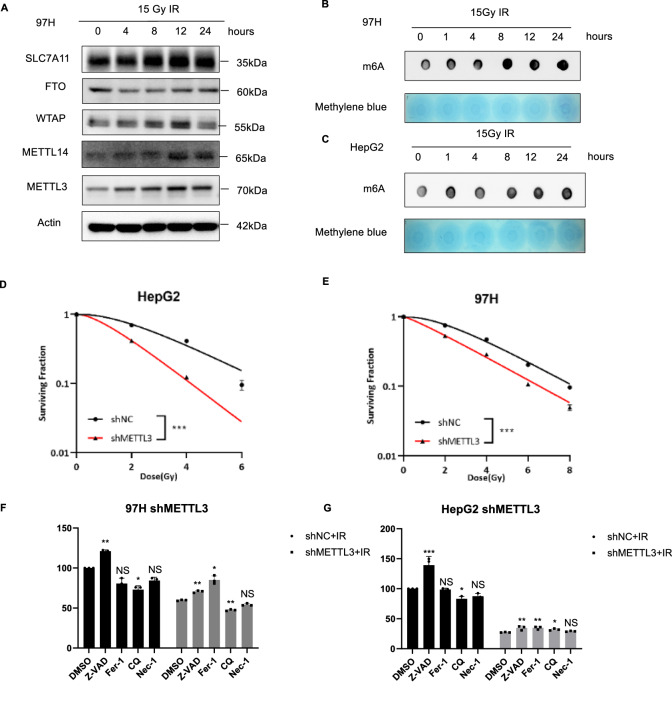
Knockdown of METTL3 enhanced radiosensitivity of HCC cells by promoting ferroptosis. **A** Western blot analysis of the effects of IR on the m^6^A modification related proteins and SLC7A11 in 97H cells at different time points after IR; Overall m^6^A levels at different time points after IR in MHCC-97H (**B**) or HepG2 (**C**) cells were detected by m^6^A dot blot; Dose responses of survival fractions of shNC, shMETTL3 HepG2 (**D**) and MHCC-97H (**E**) cells after IR; MHCC-97H (**F**) or HepG2 (**G**) shNC/shMETTL3 cells were pretreated with different inhibitors of cell death ZVAD, Fer-1, CQ and Nec-1 for 2 h and then irradiated. The cell viability was detected by CCK-8 kit. *P < 0.05, **P < 0.01, ***P < 0.001.

We aimed to explore the impact of IR on HCC, specifically focusing on the role of METTL3 and m^6^A modification. Following IR treatment, the expression of m^6^A writers METTL3, METTL14, and WTAP exhibited a gradual upregulation until 12 h, while the m^6^A eraser FTO displayed a temporary decrease followed by restoration (Fig. 2A). To assess the overall m^6^A modification in HCC cells post-IR, dot blot assays were employed. The results revealed a significant increase in the m^6^A modification in both HepG2 and MHCC-97H cells compared to the control group, persisting up to 24 h after IR (Fig. 2B, C). This suggests a dynamic modulation of m^6^A modification in response to IR, highlighting potential implications for HCC treatment strategies. We observed a remarkable reduction in the survival fraction of HepG2 and MHCC-97H cells after IR treatment (Fig. 2D, E, Supplementary Fig. S1F, G). The apoptosis inhibitor Z-VAD exhibited a remarkable rescue effect on cell death induced by IR in cells with scramble vector. While, in cells with METTL3 knockdown, the rescue of cell death by the ferroptosis inhibitor Fer-1 was notably intensified (Fig. 2F, G). This intriguing observation suggests a transformative impact of METTL3 knockdown on IR-induced HCC cell death dynamics, steering the transition from apoptosis-dominated to ferroptosis-dominated pathways.

**Fig. 3:**
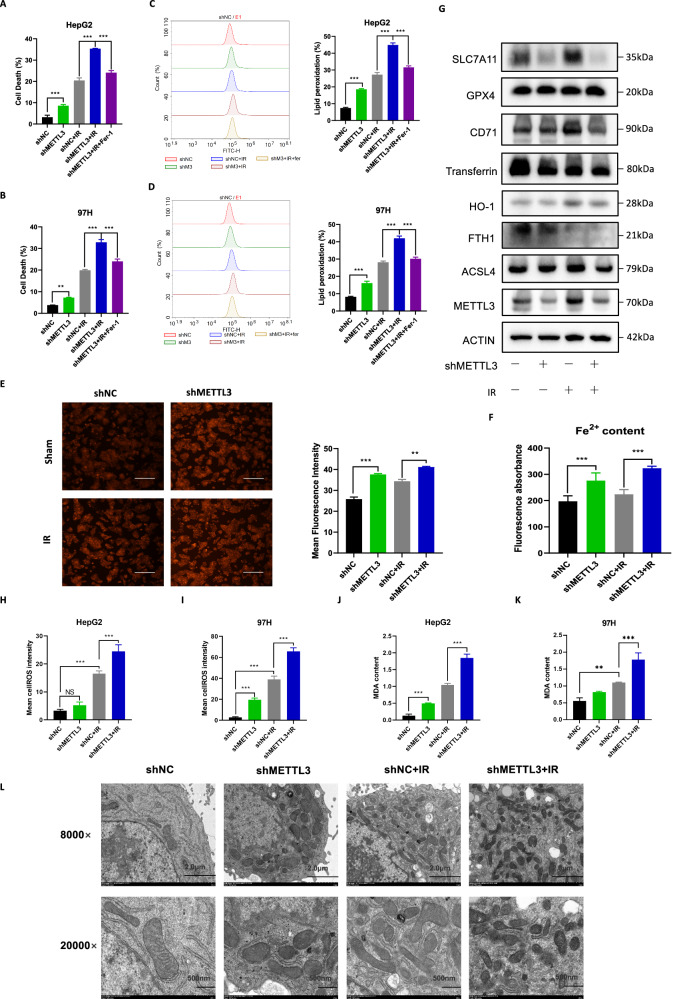
Knockdown of METTL3 promoted ferroptosis in vitro and in vivo. Cell death rates of shNC, shMETTL3 HepG2 (**A**) and MHCC-97H (**B**) cell lines treated with IR alone or together with Fer-1 were detected by Trypan blue staining; Lipid peroxidation assessment in shNC, shMETTL3 HepG2 (**C**) or MHCC-97H (**D**) cell lines after exposure to IR, bar graphs showing relative levels of lipid peroxidation by C11-BODIPY staining in the indicated cells. Error bars are means ± SD, n = 3 independent repeats; **E** Representative image of FerroOrange dye staining in shNC, shMETTL3 HepG2 cell after exposure to IR; **F** Fe^2+^ content in shNC, shMETTL3 HepG2 cell after exposure to IR was detected in automatic microplate spectrophotometer after staining with FerroOrange dye; **G** Western blot analysis of the effects of METTL3 on the ferroptosis related proteins in HepG2 cells after IR; Relative levels of ROS by DHE staining in shNC, shMETTL3 HepG2 (**H**) or MHCC-97H (**I**) cell lines after exposure to IR, Error bars are means ± SD, n = 3 independent repeats; **J**, **K** Relative levels of MDA in shNC, shMETTL3 HepG2 (**H**) or MHCC-97H (**I**) cell lines after exposure to IR, Error bars are means ± SD, n = 3 independent repeats; **L** Representative transmission electron microscopy images of mitochondria in shNC, shMETTL3 HepG2 cell line after exposure to IR, *P < 0.05, **P < 0.01, ***P < 0.001.

Similarly, the detection of cell death with Trypan blue staining confirmed that ferroptosis is one of the main causes of increased cell death after the knockdown of METTL3 combined IR (Fig. 3A, B). Consistent with these results, we observed that lipid peroxidation generated in shMETTL3 groups with or without IR treatment was much higher than in their respective control groups and the lipid peroxidation was all restored after the pretreatment of Ferrostatin-1 in two HCC cell lines (Fig. 3C, D; *P* < 0.001). In addition, intracellular Fe^2+^ content, ROS levels, and MDA content were significantly higher in the IR combined with METTL3 knockdown treatment group than in the control group (Fig. 3E–K). In Fig. 3L, a transmission electron microscope was used to observe the ultrastructure of mitochondria. IR induced mitochondria shrinkage, an increase in the mitochondrial bilayer membrane density and the disappearance of mitochondria cristae, which was exacerbated by knocking down METTL3. To explore which pathway/regulator was involved in METTL3 meditated ferroptosis in HCC, we used Western Blot analysis to detect the expression of major regulatory pathways of ferroptosis, including system x_c_^-^-GPX4 (GPX4, SLC7A11), iron metabolism (CD71, Transferrin, FTH1, and HO-1) and lipid metabolism (ACSL4), results showed that only the expression of SLC7A11 is upregulated after IR treatment, and treatment of METTL3 knockdown group with IR did not increase the SLC7A11 expression. (Fig. 3G, Supplementary Fig. S1C). We proceeded to investigate whether SLC7A11 was involved in the METTL3-mediated ferroptosis regulation, by introducing SLC7A11 into METTL3 knockdown HepG2 or MHCC-97H cells (Supplementary Fig. S3I, L). The results showed that overexpression of SLC7A11 reversed the increased lipid peroxidation levels in METTL3 knockdown HCC cells after IR treatment (Supplementary Fig. S3J, M), leading to a reduction in cell death compared with their corresponding control (Supplementary Fig. S3K, N). These findings suggest that SLC7A11 is involved in METTL3-regulated ferroptosis in HCC cells after IR treatment. Taken together, these results suggested that knockdown of METTL3 enhances HCC cells’ radiosensitivity by promoting ferroptosis.

### The expression of SLC7A11 is regulated by METTL3 mediated- m^6^A modification

**Fig. 4:**
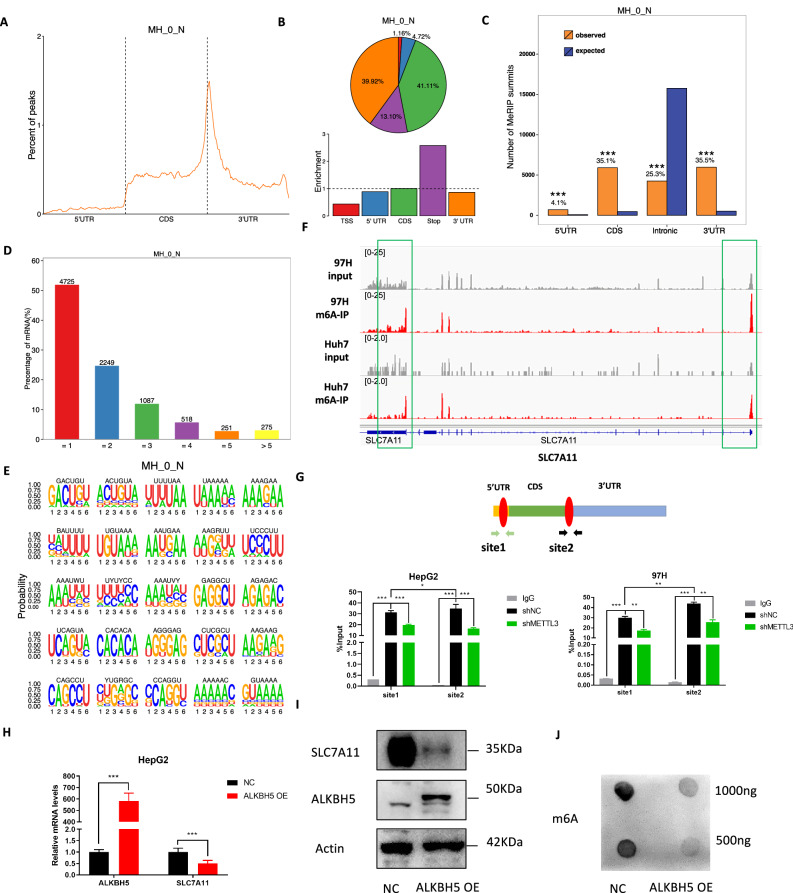
METTL3-mediated m^6^A modification on SLC7A11 transcripts. **A** Metagene distribution of m^6^A-immunocipitated reads across the length of mRNA transcripti of MHCC-97H cell line; **B** Distribution of m^6^A peaks on functional regions of mRNA transcripts; **C** Histogram of the distribution of peaks on the 4 transcript function regions (5’UTR, 3’UTR, CDS and Intronic), with expected values in blue; **D** Statistics of the number of m^6^A peaks distributed on mRNA; **E** Identification of 25 representative motifs on the mRNA regions where m6A methylation occurred was performed using HOMER software; **F** Genome Browser screenshots of MeRIP-seq read density signals on SLC7A11 mRNA in MHCC-97H and Huh7 cells; **G** Gene-specific MeRIP-qPCR for shNC and shMETTL3 HepG2 and MHCC-97H cells, m^6^A positive regions were selected based on MeRIP-seq; **H** Relative mRNA levels of ALKBH5 and SLC7A11 after ALKBH5 overexpression in HepG2 cells were detected by qRT-PCR; **I** Protein levels of ALKBH5 and SLC7A11 after ALKBH5 overexpression in HepG2 cells were detected by western blot; **J** Overall m^6^A levels after overexpression of ALKBH5 in HepG2 cells were detected by m^6^A dot blot; *P < 0.05, **P < 0.01, ***P < 0.001.

To gain a more comprehensive understanding of how METTL3 regulates ferroptosis, we conducted a MeRIP-seq analysis of RNAs from MHCC-97H and Huh7 cells, as METTL3 is the key catalytic subunit of m^6^A methyltransferase machinery. Consistent with previous studies, m^6^A peaks were found to be significantly enriched in the vicinity of the stop codon CDS and 3’UTR (Fig. 4A–D), with the consensus motif ‘RRACH’ being the most commonly enriched in peaks from all samples (Fig. 4E). We then analyzed m^6^A modifications on mRNAs of several key ferroptosis regulators in our MeRIP-seq data and found that the m^6^A peaks on SLC7A11 transcripts were significantly higher in the m^6^A-IP group than the input group in both MHCC-97H and Huh7 cells (Fig. 4F and Supplementary Fig. s2A). Using gene-specific MeRIP-qPCR assays, we confirmed the authentic deposition of RNA m^6^A modifications by METTL3 on SLC7A11 in control and METTL3 knockdown HepG2/MHCC-97H cells. Results showed that the m^6^A modifications of SLC7A11 were enriched on the 5’UTR and around the stop codon, with the modification much higher on the 3’UTR than the 5’UTR, and all these modifications were significantly reduced after METTL3 knockdown (Fig. 4G). Moreover, overexpression of ALKBH5 led to a suppression in the expression of SLC7A11 at both the RNA and protein levels (Fig. 4H, I; Supplementary Fig. S3A). Overexpression of ALKBH5 also resulted in a substantial decrease in m^6^A abundance in total RNA as detected by m^6^A dot blot assay (Fig. 4J). Collectively, these data suggest that m^6^A can positively regulate SLC7A11 expression in HCC cells.

**Fig. 5:**
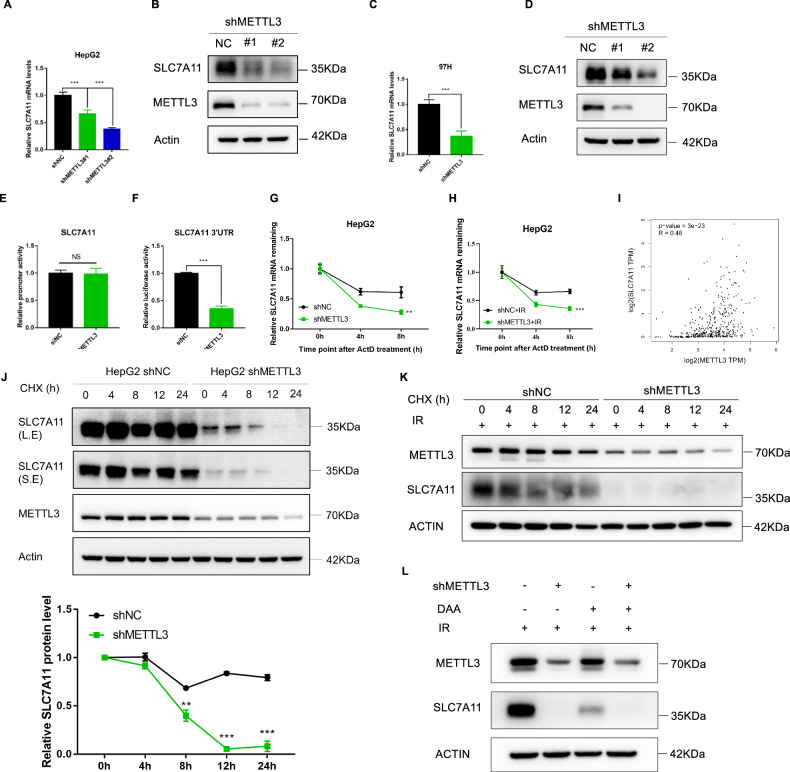
METTL3 regulated the expression of SLC7A11. Relative mRNA levels of SLC7A11 after METTL3 knockdown in HepG2 (**A**) and MHCC-97H (**C**) cell lines were detected by qRT-PCR. Protein levels of SLC7A11 and METTL3 after METTL3 knockdown in HepG2 (**B**) and MHCC-97H (**D**) cell lines were detected by western blot. **E** Relative promoter activity of SLC7A11 in 293T cells transfected with siNC or siMETTL3 was detected by dual luciferase reporter assay. **F** Relative 3’UTR activity of SLC7A11 in 293T cells transfected with siNC or siMETTL3 was detected by dual luciferase reporter assay. Relative mRNA levels of SLC7A11 in shNC, shMETTL3 HepG2 cell line treated with 5 μg/ml actinomycin D at indicated time points with (**H**) or without (**G**) IR treatment. **I** Correlation analysis of the expression of SLC7A11 and METTL3 in TCGA LIHC database. **J** The protein stability of SLC7A11 in shNC and shMETTL3 HepG2 cell lines was detected by Western blot analysis at 0, 4, 8, 12 and 24 h after treatment with CHX (100 μg/ml), and the relative levels of SLC7A11 protein were normalized against that of Actin and plotted over the time course of CHX treatments. **K** The protein stability of SLC7A11 in shNC and shMETTL3 HepG2 cell lines was detected by Western blot analysis at 0, 4, 8, 12 and 24 h after treatment with CHX (100 μg/ml) under radiotherapy conditions, **L** Protein levels of SLC7A11 and METTL3 in shNC and shMETTL3 HepG2 cell lines treated with DAA or not under radiotherapy conditions were detected by western blot. Error bars represent the mean ± SD (n = 3 independent repeats); *P < 0.05, **P < 0.01, ***P < 0.001.

SLC7A11 is the key component of system Xc^-^, which is a recognized ferroptosis gatekeeper and plays a central role in limiting lipid peroxidation [28]. In this study, we investigated how METTL3 regulates the expression of SLC7A11. Since METTL3 has RNA methyltransferase activity, we first focused on the RNA level of SLC7A11. Our results showed that the knockdown of METTL3 in HepG2 and MHCC-97H cells significantly decreased the RNA and protein expression of SLC7A11 (Fig. 5A–D). To understand the potential mechanisms, we examined the promoter activity of SLC7A11 and found no significant difference between negative control and METTL3 knockdown cells (Fig. 5E). However, knockdown of METTL3 significantly reduced the 3’UTR activity of SLC7A11 (Fig. 5F), and mRNA stability of SLC7A11 in METTL3 knockdown HCC cells was significantly less than that in control after Act-D treatment (which blocks the production of new RNA transcripts and is recognized as a commonly used drug to detect the rate of degradation of existing RNAs [34, 35]) whether or not exposed to IR (Fig. 5G, H, Supplementary Fig. S3F). In addition, we found that METTL3 was positively correlated with the SLC7A11 mRNA in HCC patients (Fig. 5I), suggesting a potential link between METTL3 and SLC7A11 in HCC patients. Furthermore, our data indicated that METTL3 can regulate the protein stability of SLC7A11, as the protein stability of SLC7A11 in the METTL3 group showed a certain reduction compared with the control group in HepG2 cells after CHX treatment (which prevents new protein synthesis without significantly affecting existing proteins, and is recognized as a commonly used drug to monitor the degradation of pre-existing proteins [36, 37]) whether or not exposed to IR (Fig. 5J, K). To further validate the relationship between SLC7A11 and METTL3-mediated m^6^A methylation, we combined treatment with the m^6^A inhibitor DAA on top of ionizing radiation treatment, and the results showed that DAA could significantly inhibit the expression of SLC7A11, but since knockdown of METTL3 had resulted in near-absence of SLC7A11 expression, DAA combined with METTL3 knockdown did not cause a further decrease in SLC7A11 expression (Fig. 5L).

### IGF2BP2 is responsible for METTL3-mediated transcriptional regulation of SLC7A11

**Fig. 6:**
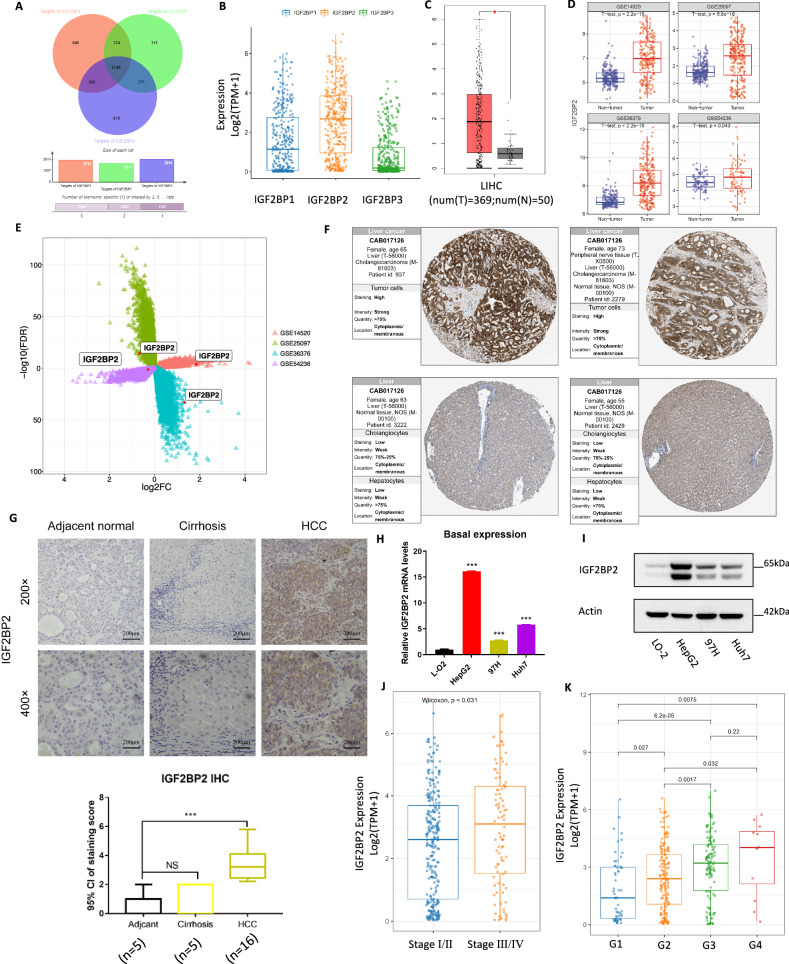
Higher expression of IGF2BP2 was associated with prognosis of human HCC. **A** Venn diagram showing the intersection of IGF2BP1, 2 and 3 downstream target mRNAs; **B** Relative expression levels of IGF2BP1, 2 and 3 mRNA in LIHC patients according to the RNA-seq data from TCGA; **C** TCGA database showed that IGF2BP2 was elevated in LIHC tissue compared with normal tissue; **D**, **E** Analysis of IGF2BP2 expression in four GEO datasets (GSE14520, GSE25097, GSE36376, GSE54236); **F** HPA database showed that IGF2BP2 was highly expressed in liver tissue of HCC patients compared to healthy controls; **G** Representative immunohistochemistry images of the expression of IGF2BP2 in HCC specimens compared with adjacent tissues, scale bar: 200 μm; **H** Relative mRNA levels of IGF2BP2 in L-O2, HepG2, MHCC-97H and Huh7 cell lines were detected by qRT-PCR; **I** Protein levels of IGF2BP2 in L-O2, HepG2, MHCC-97H and Huh7 cell lines were detected by western blot; **J**, **K** Analysis of METTL3 tumor stage (**I**) and Grade (**J**) specific expression in TCGA LIHC database; *P < 0.05, **P < 0.01, ***P < 0.001.

To elucidate the mechanisms responsible for m^6^A-mediated regulation of mRNA stability, we then found that m^6^A modification may regulate mRNA stability through the action of readers IGF2BP1 ~ 3. Although there is considerable overlap in the target genes of these readers (Fig. 6A), IGF2BP2 was found to be highly expressed in HCC compared to normal liver tissue, based on analysis of TCGA and GEO databases (Fig. 6B–E). The protein expression of IGF2BP2 was also confirmed to be higher in HCC tissues compared to normal liver tissues using the HPA database (https://www.proteinatlas.org/) (Fig. 6F) and in our collection of HCC samples using IHC (Fig. 6G). Additionally, our verification in cell lines showed that both mRNA and protein levels of IGF2BP2 were higher in HCC cell lines (HepG2, MHCC-97H, Huh7) compared to normal liver L-O2 cells (Fig. 6H, I). However, the difference in the expression of IGF2BP3 in HCC cells and hepatocytes was less pronounced than that of IGF2BP2 (Supplementary Fig. S1B). Furthermore, analysis of the TCGA database revealed that IGF2BP2 expression was significantly higher in stage III/IV HCC samples than in stage I/II, and its expression increased progressively with tumor grade (Fig. 6J, K). For this reason, we focused on IGF2BP2 for further investigation.

**Fig. 7:**
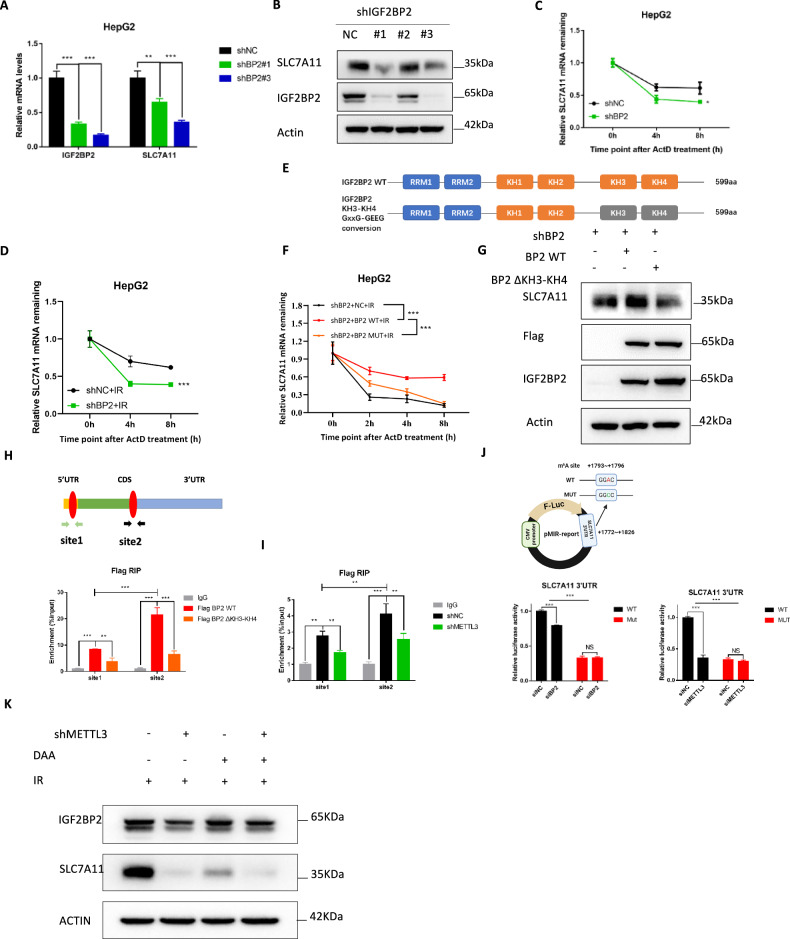
IGF2BP2 functioned as m^6^A reader to enhance SLC7A11 mRNA stability. **A** Relative mRNA levels of IGF2BP2 and SLC7A11 in HepG2 after knockdown of IGF2BP2 were detected by qRT-qPCR; **B** Protein levels of IGF2BP2 and SLC7A11 in HepG2 after knockdown of IGF2BP2 were detected by western blot; Relative mRNA levels of SLC7A11 in shNC and shIGF2BP2 HepG2 cells treated with 5 μg/ml actinomycin D at indicated time points with (**D**) or without (**C**) IR treatment; **E** Schematic diagram of wild-type and ΔKH3-KH4 GxxG-GEEG conversion IGF2BP2; **F** Relative mRNA levels of SLC7A11 in NC, WT and Mutant IGF2BP2 overexpressed HepG2 shBP2 cells treated with 5 μg/ml actinomycin D at indicated time points under radiotherapy conditions; **G** Protein levels of IGF2BP2 and SLC7A11 in HepG2 shBP2 cells after overexpression of WT or ΔKH3-KH4 mutant IGF2BP2 were detected by western blot; **H** RIP-qPCR analysis of SLC7A11 mRNA 5’UTR and 3’UTR in WT or ΔKH3-KH4 mutant IGF2BP2 overexpressed shIGF2BP2 HepG2 cells; **I** RIP-qPCR analysis of SLC7A11 mRNA 5’UTR and 3’UTR in shNC or shMETTL3 HepG2 cells; **J** Schematic generation strategy for the pMIR-report luciferase reporters containing WT and Mut (GGAC to GGCC) SLC7A11 3′-UTR; Luciferase activities from the indicated pMIR vector were measured in 293T cells with or without IGF2BP2 knockdown; **K** Protein levels of SLC7A11 and METTL3 in shNC and shIGF2BP2 HepG2 cell lines treated with DAA or not under radiotherapy conditions were detected by western blot. *P < 0.05, **P < 0.01, ***P < 0.001.

To explore the relationship between IGF2BP2 and SLC7A11, we constructed a stable knockdown of IGF2BP2 in HepG2 cells. The results showed a significant reduction in both mRNA and protein levels of SLC7A11 upon knockdown of IGF2BP2 (Fig. 7A, B). Given that IGF2BP2 is known to act as an m^6^A reader, we further examined its effect on SLC7A11 mRNA stability. Our results indicated that the knockdown of IGF2BP2 in HepG2 cells significantly decreased the half-life of SLC7A11 mRNA whether under radiotherapy condition or not (Fig. 7C, D). Previous study reveals that KH3-4 di-domains play essential roles in IGF2BP binding to m^6^A-modified mRNAs and in the regulation of target gene expression [19], we constructed the IGF2BP2 KH3-4 GxxG to GEEG conversion (ΔKH3-KH4) in pLVX-HA vector and confirmed the overexpression efficiency of IGF2BP2 by RT-qPCR and western blot analysis (Fig. 7E, Supplementary Fig. S2B). Our results showed that only wild-type IGF2BP2, but not ΔKH3-KH4, could increase the mRNA and protein expression of SLC7A11 in HepG2 and MHCC-97H cell lines (Fig. 7F, G and Supplementary Fig. S3G, H). Additionally, only wild-type IGF2BP2 significantly enhanced the mRNA stability of SLC7A11, while the ΔKH3-KH4 had no significant effect on SLC7A11 mRNA stability (Fig. 7F, Supplementary Fig. S3H). To verify the binding between IGF2BP2 and SLC7A11 mRNA, we performed RIP-qPCR in HepG2 cells with flag-tagged WT IGF2BP2 or ΔKH3-KH4 IGF2BP2, followed by immunoprecipitation with anti-flag magnetic beads. The results showed that IGF2BP2 interacted with SLC7A11 mRNA significantly at site 1 on the 5’UTR and site 2 on the 3’UTR, with higher binding intensity at site 2. These bindings were significantly suppressed in ΔKH3-KH4 cells (Fig. 7G, H). Furthermore, we examined the binding of IGF2BP2 with SLC7A11 in METTL3 knockdown cells and found that the binding intensity of IGF2BP2 at site 2 was significantly higher than that at site 1, and the binding ability of IGF2BP2 with both sites was significantly weakened after knockdown of METTL3 (Fig. 7I). This revealed that IGF2BP2 preferred to bind with the 3’UTR of SLC7A11 mRNA and that the binding was m^6^A-dependent. Lastly, we examined whether IGF2BP2 stabilizes SLC7A11 mRNA via the putative m^6^A site. We generated the wild-type (WT) SLC7A11 3’UTR and mutant (Mut) 3’UTR (GGAC to GGCC) downstream of the luciferase encoding region in the pMIR-report plasmid (Supplementary Fig. S2C). Our results suggested that knockdown of IGF2BP2 decreased the luciferase activity of WT 3’UTR instead of Mut 3’UTR of SLC7A11 (Fig. 7J). To understand the expression pattern of IGF2BP2 after IR, we performed a western blot, and the results showed no significant changes in its expression at different time points post-IR (Supplementary Fig. S1H, I). To further validate the relationship between SLC7A11 and IGF2BP2-mediated recognition and regulation of the m^6^A locus, we combined treatment with DAA on top of ionizing radiation treatment, results showed that DAA could significantly inhibit the expression of SLC7A11 and DAA combined with IGF2BP2 knockdown treatment further reduced the expression level of SLC7A11 (Fig. 7K).

### Knockdown of IGF2BP2 enhanced radiosensitivity of HCC cells by promoting ferroptosis

**Fig. 8:**
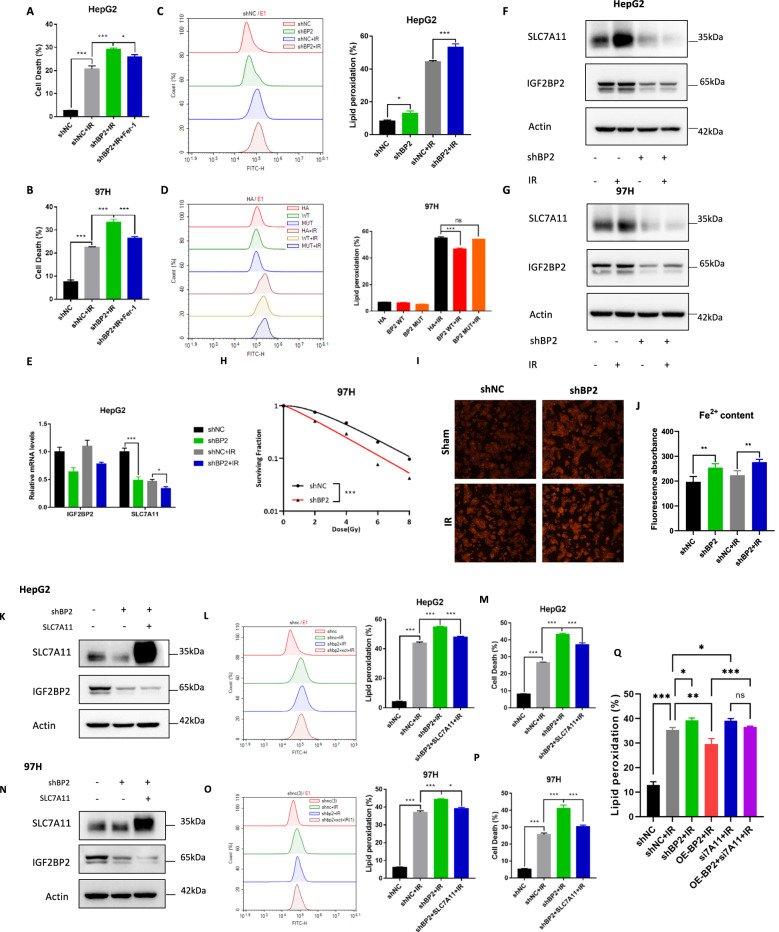
Knockdown of IGF2BP2 enhanced radiosensitivity of HCC cells by promoting ferroptosis. Cell death rates of shNC, shIGF2BP2 HepG2 (**A**) and MHCC-97H (**B**) cell lines treated with IR alone or together with Fer-1 were detected by Trypan blue staining; **C** Lipid peroxidation assessment in shNC, shIGF2BP2 HepG2 cells after exposure to IR, bar graphs showing relative levels of lipid peroxidation by C11-BODIPY staining in the indicated cells. Error bars are means ± SD, n = 3 independent repeats; **D** Lipid peroxidation assessment in NC, WT and ΔKH3-KH4 IGF2BP2 overexpressed MHCC-97H cells after exposure to IR, bar graphs showing relative levels of lipid peroxidation by C11-BODIPY staining in the indicated cells. Error bars are means ± SD, n = 3 independent repeats; **E** Relative mRNA levels of IGF2BP2 and SLC7A11 in shNC, shIGF2BP2 HepG2 cells treated with or without IR were detected with qRT-PCR; Protein levels of IGF2BP2 and SLC7A11 in shNC, shIGF2BP2 HepG2 (**F**) and MHCC-97H (**G**) cells treated with or without IR were detected with western blot; **H** Dose responses of survival fractions of shNC, shIGF2BP2 97H cells after IR; **I** Representative image of FerroOrange dye staining in shNC, shBP2 HepG2 cell after exposure to IR; **J** Fe^2+^ content in shNC, shBP2 HepG2 cell after exposure to IR was detected in automatic microplate spectrophotometer after staining with FerroOrange dye; Protein levels of IGF2BP2 and SLC7A11 in shNC, shIGF2BP2 and shIGF2BP2 + SLC7A11 overexpression HepG2 (**K**) or MHCC-97H (**N**) cell lines were detected by western blot; Lipid peroxidation assessment in shNC, shIGF2BP2 and shIGF2BP2 + SLC7A11 overexpression HepG2 (**L**) or MHCC-97H (**O**) cell lines after exposure to IR, bar graphs showing relative levels of lipid peroxidation by C11-BODIPY staining in the indicated cells. Error bars are means ± SD, n = 3 independent repeats; Cell death rates of shNC, shIGF2BP2 and shIGF2BP2 + SLC7A11 overexpression HepG2 (**M**) or MHCC-97H (**P**) cell lines treated with IR were detected by Trypan blue staining; **Q** Lipid peroxidation assessment in shNC, shNC+IR, IGF2BP2 knockdown+IR, IGF2BP2 overexpression+IR, SLC7A11 knockdown+IR and OE-BP2+si7A11+IR HepG2 cells by C11-BODIPY staining. *P < 0.05, **P < 0.01, ***P < 0.001.

Firstly, knockdown of IGF2BP2 induced a significant decrease in the proliferative capacity of HCC cells (Supplementary Fig. S3E). To further explore the effect of IGF2BP2 on ferroptosis, we investigated the extent of cell death with Fer-1 after IR treatment in IGF2BP2 knockdown cells, and results indicated that ferroptosis is one of the main causes of IR-induced cell death after knockdown IGF2BP2 (Fig. 8A, B). Additionally, the lipid peroxidation generated in shIGF2BP2 groups, with or without IR treatment, was significantly higher than their respective control groups (Fig. 8C, *P* < 0.001). Conversely, overexpression of WT IGF2BP2 in MHCC-97H cells reduced IR-induced lipid peroxidation compared to controls, whereas this effect was not observed for mutant IGF2BP2 (Fig. 8D).

We next investigated the expression of SLC7A11 in HepG2 cells at the mRNA and protein levels after IR treatment. IR induced a decrease in SLC7A11 expression, which was further reduced after IGF2BP2 knockdown at both mRNA and protein levels (Fig. 8E–G). However, overexpression of wild-type IGF2BP2 partially reversed the reduction in SLC7A11 expression after IR treatment (Supplementary Fig. S3B). Knockdown of IGF2BP2 resulted in a remarkable reduction in the survival fraction of MHCC-97H cells after IR (Fig. 8H, Supplementary Fig. S1G). Although overexpression of wild-type IGF2BP2 could somewhat improve survival under 2 Gy and 4 Gy, this effect was not significant (Supplementary Fig. S3C), possibly due to the higher basal expression of IGF2BP2 in HCC cells. Moreover, HepG2 cells showed higher Fe^2+^ content in the shBP2±IR group than in their respective control groups (Fig. 8I, J). Furthermore, we overexpressed SLC7A11 in IGF2BP2 knockdown cells (Fig. 8K, N). Overexpression of SLC7A11 also alleviated the increased lipid peroxidation (Fig. 8L, O) and cell death (Fig. 8M, P) of IGF2BP2 knockdown cells after IR treatment. This implies that SLC7A11 is involved in IGF2BP2-regulated ferroptosis in HCC cells after IR treatment. To further investigate the interaction between IGF2BP2 and SLC7A11 in the regulation of ferroptosis, we set up the following subgroups to detect the levels of lipid peroxidation in the context of radiotherapy, and the results showed that knockdown of IGF2BP2 and SLC7A11 resulted in a significant increase in lipid peroxidation levels. In contrast, overexpression of IGF2BP2 resulted in a decrease in peroxidation levels. In addition, knockdown of SLC7A11 on the basis of overexpression of IGF2BP2 resulted in a significant increase in lipid peroxidation level, which was somewhat but not significantly lower than that of knockdown of SLC7A11 alone, which may be related to the mechanism by which IGF2BP2 regulates the stability of SLC7A11 mRNA through m^6^A (Fig. 8Q).

### SOCS2 is responsible for METTL3-mediated regulation of SLC7A11 protein stability

**Fig. 9:**
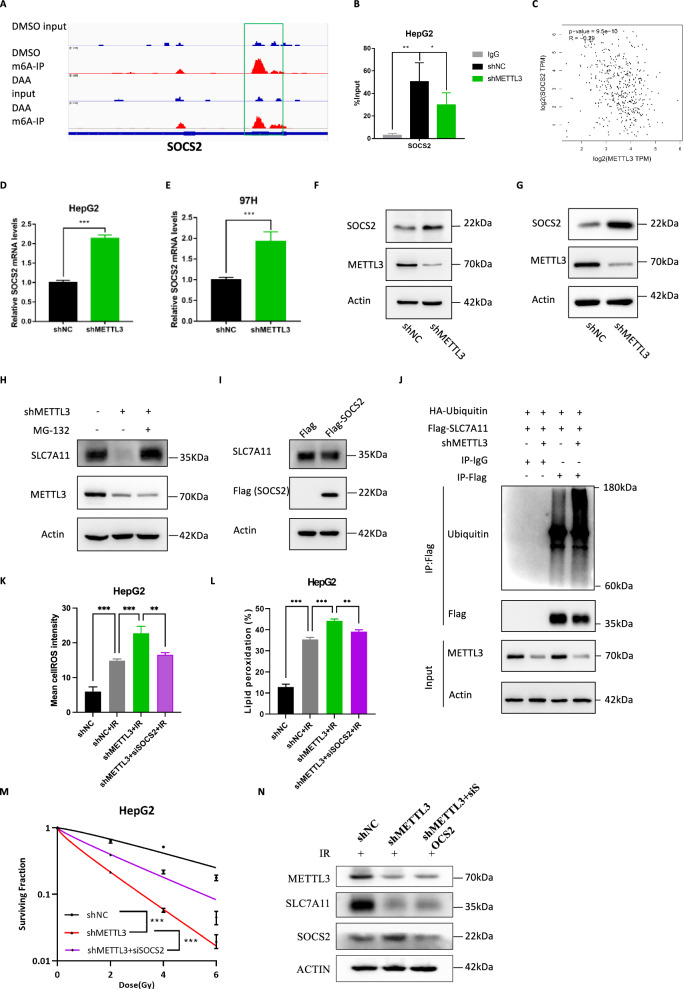
METTL3 regulates SLC7A11 ubiquitination and protein stability through SOCS2. **A** Genome Browser screenshots of MeRIP-seq read density signals on SOCS2 mRNA in MHCC-97H treated with DMSO or DAA; **B** Gene-specific MeRIP-qPCR for shNC and shMETTL3 HepG2 cells, m^6^A positive region on SOCS2 transcript was selected based on MeRIP-seq; **C** Correlation analysis of the expression of SOCS2 and METTL3 in TCGA LIHC database; Relative mRNA level of SOCS2 in HepG2 (**D**) and MHCC-97H (**E**) cells with METTL3 knockdown were detected by qRT-PCR; **F**, **G** Protein levels of SOCS2 and METTL3 in shNC, shMETTL3 HepG2 (**F**) and MHCC-97H cells were detected by western blot; **H** Protein levels of SLC7A11 and METTL3 in shNC, shMETTL3 HepG2 cells treated with 10 μM MG-132 for 12 h were detected by western blot; **I** Protein levels of SLC7A11 and Flag-tag (SOCS2) in HepG2 cells transfected with pcDNA3.1-flag SOCS2 were detected by western blot; **J** Anti-Ub immunoblotting assay of SLC7A11 polyubiquitination in shNC and shMETTL3 HepG2 cells. **K** Relative levels of ROS by DHE staining in shNC, shNC+IR, shMETTL3+IR and shMETTL3+siSOCS2+IR HepG2 cells, error bars are means ± SD, n = 3 independent repeats; **L** Lipid peroxidation assessment in shNC, shNC+IR, shMETTL3+IR and shMETTL3+siSOCS2+IR HepG2 cells by C11-BODIPY staining, error bars are means ± SD, n = 3 independent repeats; **M** Dose responses of survival fractions of shNC, shMETTL3 and shMETTL3+siSOCS2 HepG2 cells after IR; **N** Protein levels of SOCS2, METTL3 and SLC7A11 in shNC, shMETTL3 and shMETTL3+siSOCS2 HepG2 cells under radiotherapy conditions were detected by western blot; *P < 0.05, **P < 0.01, ***P < 0.001.

Besides, we would like to explore the mechanism by which METTL3 regulates the protein stability of SLC7A11, for in our previous results we found that METTL3 can regulate not only the RNA stability but also the protein stability of SLC7A11. We screened through bioinformatics and literatures [24] and predicted suppressor of cytokine signaling 2 (SOCS2) as a potential target of METTL3-mediated m^6^A modification. To verify the underlying mechanism, we screened for the m^6^A peaks of SOCS2 in our MeRIP-seq data and found a significant enrichment in SOCS2 3’UTR. This m^6^A peak was somewhat reduced after treatment with m^6^A inhibitor DAA (3-Deazaadenosine) (Fig. 9A). Using gene-specific MeRIP-qPCR assays, we confirmed the authentic deposition of RNA m^6^A modifications by METTL3 on SOCS2 in control and METTL3 knockdown HepG2 cells, and this modification was significantly reduced after METTL3 knockdown (Fig. 9B). In addition, expression correlation analysis showed a negative correlation between SLC7A11 and SOCS2 expression in TCGA LIHC samples (Fig. 9C). To investigate the expression pattern of SOCS2 after IR, we performed western blot analysis at different time points after IR. The results showed that in HepG2 cells, the expression level of SOCS2 began to decrease at 12 h after IR treatment, and in 97H cells, it decreased significantly at 24 h after IR treatment (Supplementary Fig. S1H, I). Furthermore, knockdown of METTL3 in HepG2 and MHCC-97H cells induced upregulation of SOCS2 both in RNA and protein levels (Fig. 9D–G). Treatment with the proteasome inhibitor MG132 on the basis of knockdown of METTL3 returned SLC7A11 expression to some extent (Fig. 9H, Supplementary Fig. S3D). Overexpression of SOCS2 did reduce SLC7A11 expression (Fig. 9I). Ubiquitination IP experiment showed that the ubiquitination of SLC7A11 in HepG2 cells with knocked-down METTL3 was significantly higher than that in control. This indicated that METTL3 could reduce the ubiquitination of SLC7A11 (Fig. 9J). In addition, to further understand the role of SOCS2 in METTL3-mediated regulation of SLC7A11 expression and ferroptosis, we further knocked down SOCS2 on the basis of knockdown of METTL3, and the results showed that the double knockdown group significantly reduced intracellular ROS levels (Fig. 9K), lipid peroxidation levels (Fig. 9L) and radiosensitivity of HCC cells (Fig. 9M). Importantly, further knockdown of SOCS2 on the basis of knockdown of METTL3 restored the protein expression of SLC7A11 to some extent (Fig. 9N).

### METTL3/IGF2BP2 mediated in vivo radiosensitivity and the clinical prognosis

**Fig. 10:**
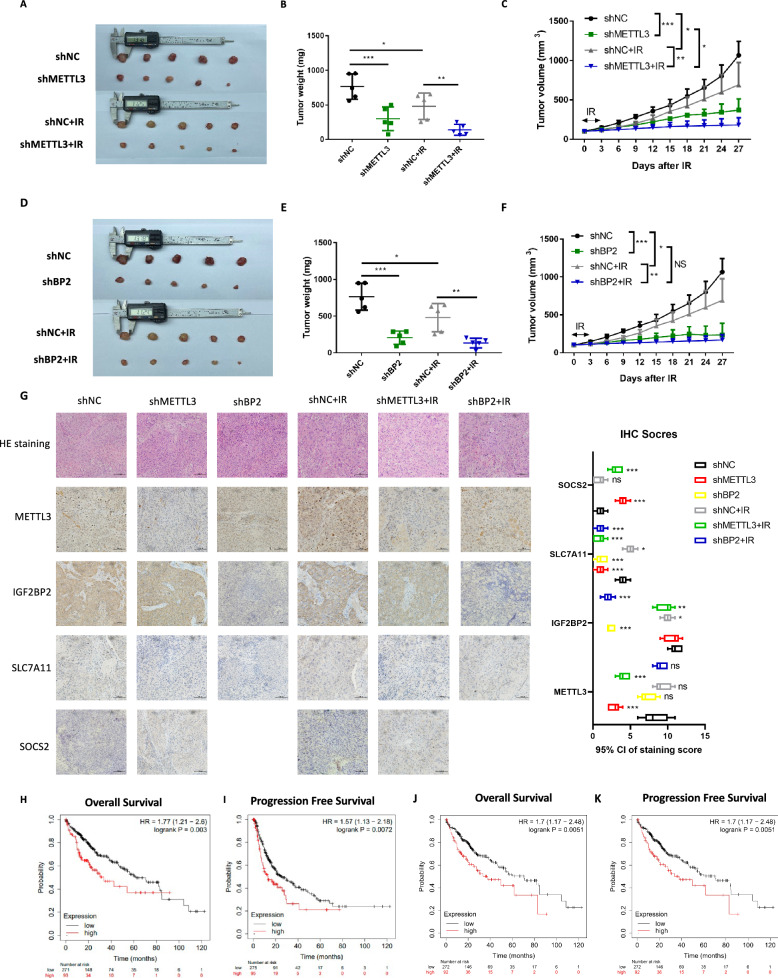
Knockdown of METTL3/IGF2BP2 promoted radiosensitivity of HCC in vivo. **A** Images of isolated tumors from mice euthanized at the end of experiment; **B** Individual tumor weights of each treatment group; **C** Tumor volumes over the experimental period; **D** Images of isolated tumors from mice euthanized at the end of experiment; **E** Individual tumor weights of each treatment group; **F** Tumor volumes over the experimental period; **G** Representative immunohistochemistry images of the expression of METTL3, IGF2BP2, SLC7A11 and SOCS2 in shNC, shMETTL3, shBP2, shNC+IR, shMETTL3+IR and shBP2+IR mouse xenograft tumor tissue, scale bar: 100 μm and IHC-staining scores of each marker, data are plotted as the means of 95% confidence interval ± s.d; **H**, **I** Kaplan–Meier analyses of the correlation between METTL3 mRNA levels and the overall survival (**H**) or progression free survival (**I**) in 364 patients with HCC; **J**, **K** Kaplan–Meier analyses of the correlation between METTL3 mRNA levels and the overall survival (**J**) or progression free survival (**K**) in 364 patients with HCC; *P < 0.05, **P < 0.01, ***P < 0.001.

To investigate the relationship between METTL3 or IGF2BP2 and radiosensitivity in vivo, we established a mouse xenograft model and irradiated the primary tumor site using irradiator. We found that IR induced a reduction in xenograft volume, which was more pronounced in shMETTL3 cells than in shNC cells (Fig. 10A–C; Supplementary Fig. S1H–J). Similarly, both IR and IGF2BP2 knockdown were effective in suppressing xenograft growth. However, the combined treatment of IGF2BP2 knockdown and IR did not show a more pronounced inhibitory effect. This could be attributed to the fact that knockdown of IGF2BP2 alone already resulted in a significant reduction in tumor volume (Fig. 10D–F; Supplementary Fig. S1J–L). To validate the METTL3-SOCS2-SLC7A11 and METTL3-IGF2BP2-SLC7A11 axis in vivo, we performed immunohistochemical analyses, stained for each marker, and consistent with the in vitro results, knockdown of METTL3 with or without treatment with IR increased the expression of SOCS2 and decreased SLC7A11 expression, while knockdown of IGF2BP2 decreased SLC7A11 expression (Fig. 10G).

Furthermore, high METTL3 expression was significantly associated with poor prognosis (overall and progression-free survival) in HCC patients (Fig. 10H, I). Similarly, the expression of IGF2BP2 was significantly correlated with the prognosis of HCC patients (Fig. 10J, K), suggesting its potential importance in the pathogenesis of HCC. Taken together, these findings suggest that low METTL3/IGF2BP2 expression is associated with high radiosensitivity in vivo and better clinical prognosis in HCC.

## Discussion

### METTL3 contributed to the radioresistance by regulating the expression of SLC7A11 in HCC cells

According to the most recent National Comprehensive cancer network (NCCN) guideline, radiotherapy is an option for patient with unresectable or inoperable HCC [4]. Previous studies have shown that ferroptosis promotes the radiosensitivity of HCC cells [31, 38]. In this study, we found that METTL3 was significantly upregulated in HCC tissues compared with normal liver tissues, then we focused on m^6^A modification dynamics. Following IR treatment, there was a notable upregulation in the expression of the major m^6^A writer until 24 hours, paralleled by an increase in overall m^6^A modification. These findings underscore the significance of m^6^A modification in the radiobiological responses of HCC. Remarkably, knockdown of METTL3, a pivotal methyltransferase for m^6^A modification, substantially augmented the radiosensitivity of HCC cells, accompanied by heightened ferroptosis induction across all cell death modalities. This suggests a potential role for the METTL3/m^6^A in mediating HCC radiosensitization, particularly through the regulation of ferroptosis pathways. Furthermore, our observations revealed a temporal upregulation of the major negative regulator of ferroptosis, SLC7A11, post-irradiation, potentially reflecting an early protective cellular response. Intriguingly, METTL3 knockdown abrogated this upregulation, fostering increased ferroptosis levels and heightened radiation sensitivity in HCC cells

### METTL3 maintained mRNA stability of SLC7A11 in an m^6^A/IGF2BP2- dependent manner, via the binding with the 3’UTR (+1795) site of SLC7A11

The m^6^A modification can regulate nearly all stages in the life cycle of RNA, including transcription, splicing, and translation modulation [39]. METTL3 is the main component of the methyltransferase complex (MTC) and performs the function of methyltransferase [21]. Our data showed that METTL3 methylates both the 5’UTR and 3’UTR of SLC7A11 mRNA, and the level of m^6^A modification on the 3’UTR is higher and more dynamic compared to the 5’UTR. Further studies revealed that m^6^A reader IGF2BP2 enhances SLC7A11 mRNA stability by recognizing the m^6^A site on its 3’UTR. Our results show that the expression of IGF2BP2 is not dependent on IR dose or time, indicating that the post-IR expression changes of SLC7A11 are primarily caused by METTL3. The increased expression of METTL3 post-IR leads to an increase in m^6^A modification abundance on the SLC7A11 transcript, which subsequently recruits IGF2BP2, enhancing its mRNA stability and, in turn, increasing its expression. However, the co-factors of IGF2BP2 that may enhance the stability of SLC7A11 mRNA are unknown, such as ELAV-like RNA binding protein 1 (ELAVL1; also known as HuR), matrin 3 (MATR3) and poly(A)-binding protein cytoplasmic 1 (PABPC1) [19], and require further exploration.

Our study found that METTL3 could methylate the 5’UTR and 3’UTR of SLC7A11 in HCC cells. The m^6^A modification of the 3’UTR (+1795) site has an important regulatory role in the expression on SLC7A11. However, the regulatory role of the m^6^A site of the 5’UTR in expression remains to be investigated. The RIP-qPCR results showed that IGF2BP2 could not only bind to the m^6^A site of SLC7A11 3’UTR but also the m^6^A site of its 5’UTR, although the binding abundance is significantly lower than that of the 3’UTR. A regulatory role cannot be ruled out. Additionally, the m^6^A site of the 5’UTR may promote translation initiation by binding to YTHDF1 [40, 41], or promote cap-independent translation by recruiting eIF3a to a nearby translation initiation site [42], or simply constitutive modifications. These possibilities remain to be further investigated. Once it was reported that in oral squamous carcinoma (OSCC) METTL3 enhanced the stability of SLC7A11 and regulated the proliferation, invasive and migrative ability of tumor cells, and thus promoted the progression of OSCC [43]. Their findings were in good agreement with ours to some extent, but we further determined in HCC that (1) the regulation of SLC7A11 by IGF2BP2 was m^6^A-dependent by RIP-qPCR of mutations in the structural domain of IGF2BP2, (2) identified the key m^6^A modification sites (+1795) that regulate the expression of SLC7A11 and (3) reversible regulation of SLC7A11 expression by m^6^A was confirmed by overexpression of ALKBH5. In addition, another study in lung adenocarcinoma (LUAD) demonstrated that METTL3 enhances RNA stability and promotes translation of SLC7A11 through m^6^A-mediated binding of YTHDF1 and YTHDC2, YTHDC2 prefers to bind to m^6^A-modified SLC7A11 mRNA and promoting its decay [44]. The regulation of SLC7A11 expression by m^6^A modification is heterogeneous. Our study clarifies the m^6^A regulatory pattern of SLC7A11 in HCC and identifies the modification sites that are critical for it, which has important implications for the clinical treatment of HCC.

### METTL3 decreased the ubiquitination of SLC7A11 protein through the m6A/YTHDF2/SOCS2 axis

Fer-1 rescued cell death in the shMETTL3+IR group than in the shIGF2BP2+IR group, this suggests that other pathways contribute to the METTL3-induced ferroptosis besides IGF2BP2. We showed that METTL3 methylates SOCS2 mRNA and induces its degradation. The decrease in SOCS2 expression reduces the ubiquitination level of SLC7A11 and thus stabilizes its protein expression, and phenomenologically validated such a mechanism. Therefore, METTL3 can regulate the expression of SLC7A11 through both RNA and protein levels, confirming that METTL3 regulates ferroptosis not only through the IGF2BP2 pathway. Of course, it cannot be excluded that other pathways involved in METTL3 regulated ferroptosis.

Based on the present results, we would outline the processes of METTL3 knockdown promoted HCC cells radiosensitisation and ferroptosis. In HCC cells, METTL3 mediated the methylation modification of m^6^A sites of SLC7A11 and SOCS2 mRNA, however, the fates of these two molecules are different. The m^6^A reader IGF2BP2 enhancesd the stability of SLC7A11 by recognizing and binding to the m^6^A modification on SLC7A11. However, the m^6^A site of SOCS2 mRNA was recognized and bound by other m^6^A reader-YTHDF2, thus contributing to its degradation.

SOCS2 could serve as a bridge between SLC7A11 and ubiquitin, utilizing its SH2 region to recognize certain phosphorylated tyrosine residues at SLC7A11-NTD, recruiting ubiquitin through BOX region, thereby transferring ubiquitin to SLC7A11. Reduced expression of SOCS2 decreased the ubiquitination of SLC7A11, thereby maintaining its protein expression. In conclusion, our study provides evidence that METTL3 regulates the expression of SLC7A11 at both the mRNA and protein levels. In HCC cells with METTL3 knockdown, the lack of m^6^A modification on SLC7A11 and SOCS2 mRNAs resulted the decrease of SLC7A11 mRNA and protein.

### Low METTL3/IGF2BP2 expression represents high radiosensitivity of HCC

Many biological factors affect radiosensitivity, including hypoxia, the ability to undergo accelerated repopulation, enrichment in cancer stem cell (CSC) numbers, and immune responses, etc. In two retrospective cohorts of patients with bladder cancer, high levels of MRE11 expression on immunohistochemistry (IHC) were associated with improved cancer-specific survival in patients who received radiotherapy but not in those who underwent surgery [45, 46]. In breast cancer, Speers et al. [47] derived a 51-gene signature by irradiating multiple cell lines and correlating SF2 values with gene expression data. Transcriptome-wide expression profiling of 136 muscle-invasive bladder cancer samples identified several signatures, including one related to T cell activation, which was associated with improved disease-specific survival in patients receiving chemoradiotherapy [48].

However, there are few reports on m^6^A-related genes as predictive signatures for radiosensitivity. In this study, we found that HCC cells with low METTL3/IGF2BP2 expression exhibited higher levels of ferroptosis after irradiation, as well as higher radiosensitivity. In vivo experiments confirmed that tumors with low METTL3 expression were more sensitive to radiotherapy. Nevertheless, more studies are needed to explore whether other m^6^A-related genes are associated with the radiosensitivity of HCC and whether METTL3/IGF2BP2 can predict the radiosensitivity of pan-cancer.

In summary, we provided compelling evidence demonstrating that METTL3 regulates the radiosensitivity of HCC cells by modulating the switch from apoptosis to ferroptosis. Mechanistically, METTL3 regulates the RNA stability of SLC7A11 through an IGF2BP2-dependent manner and indirectly controls the ubiquitination level of SLC7A11 via the m^6^A/YTHDF2/SOCS2 axis to subsequently regulate ferroptosis in HCC cells. The methylation of 3’UTR is critical for m^6^A-mediated mRNA stability. METTL3 or IGF2BP2 may be promising targets for predicting radiotherapy sensitivity in HCC patients and may represent a promising therapeutic strategy for HCC.

## Supplementary information

Supplementary material

Original western blots

## Data Availability

The datasets supporting the conclusions of this article are included within the article and its supplementary information files and from the corresponding author upon request.
