# Supplementary material for: METTL3 inhibition promotes radiosensitivity in hepatocellular carcinoma through regulation of SLC7A11 expression
Source: Cell Death Dis. 2025 Jan 11;16(1):9. doi: 10.1038/s41419-024-07317-x (PMC11724875; doi:10.1038/s41419-024-07317-x)
Supplement: Supplementary file 1 — Supplementary material [file 41419_2024_7317_MOESM1_ESM.pdf]

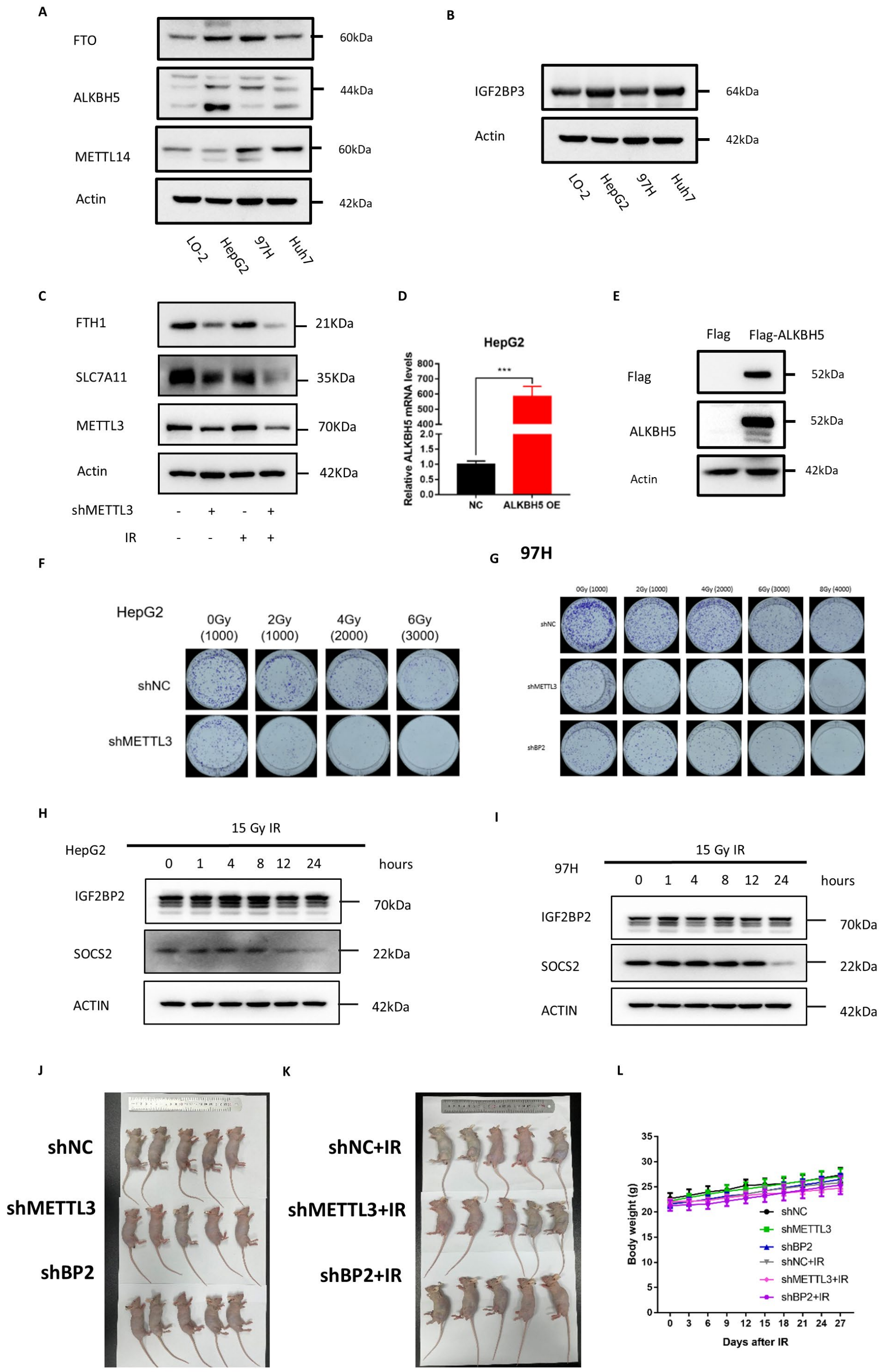
A
FTO
ALKBH5
METTL14
Actin
60kDa
44kDa
60kDa
42kDa
LO-2
HepG2
97H
Huh7
B
IGF2BP3
Actin
64kDa
42kDa
LO-2
HepG2
97H
Huh7
C
FTH1
SLC7A11
METTL3
Actin
21KDa
35KDa
70KDa
42KDa
shMETTL3 - + - +
IR - - + +
D
HepG2
Relative ALKBH5 mRNA levels
***
NC
ALKBH5 OE
E
Flag
Flag-ALKBH5
Flag
ALKBH5
Actin
52kDa
52kDa
42kDa
F
HepG2
0Gy (1000)
2Gy (1000)
4Gy (2000)
6Gy (3000)
shNC
shMETTL3
G
97H
H
15 Gy IR
HepG2
0 1 4 8 12 24
hours
IGF2BP2
SOCS2
ACTIN
70kDa
22kDa
42kDa
I
15 Gy IR
97H
0 1 4 8 12 24
hours
IGF2BP2
SOCS2
ACTIN
70kDa
22kDa
42kDa
J
shNC
shMETTL3
shBP2
K
shNC+IR
shMETTL3+IR
shBP2+IR
L
Body weight (g)
Days after IR
shNC
shMETTL3
shBP2
shNC+IR
shMETTL3+IR
shBP2+IR

Supplementary Fig 1. Knockdown of METTL3 and IGF2BP2 inhibited HCC in vitro and in vivo.

A: Protein levels of FTO, ALKBH5 and METTL14 in L-O2, HepG2, MHCC-97H and Huh7 were detected by western blot; B: Protein level of IGF2BP2 in L-O2, HepG2, MHCC-97H and Huh7 were detected by western blot; C: Western blot analysis of the effects of METTL3 on the ferroptosis related proteins in MHCC-97H cells after IR; D: The overexpression efficiency of ALKBH5 in HepG2 cells were detected by qRT-PCR; E: Protein levels of ALKBH5 and flag-tag after ALKBH5 overexpression in HepG2 cells were detected by western blot; F: Representative images of colony formation of shNC, shMETTL3 HepG2 cells after IR; G: Representative images of colony formation of shNC, shMETTL3 and shIGF2BP2 MHCC-97H cells after IR; H, I: Protein level of IGF2BP2 and SOCS2 in HepG2 (H) and MHCC-97H (I) at different time points after IR were detected by western blot; J, K: Representative images of subcutaneously implanted tumors in nude mice; L: Individual mouse body weights for each treatment group. *$P < 0.05$, **$P < 0.01$, ***$P < 0.001$.

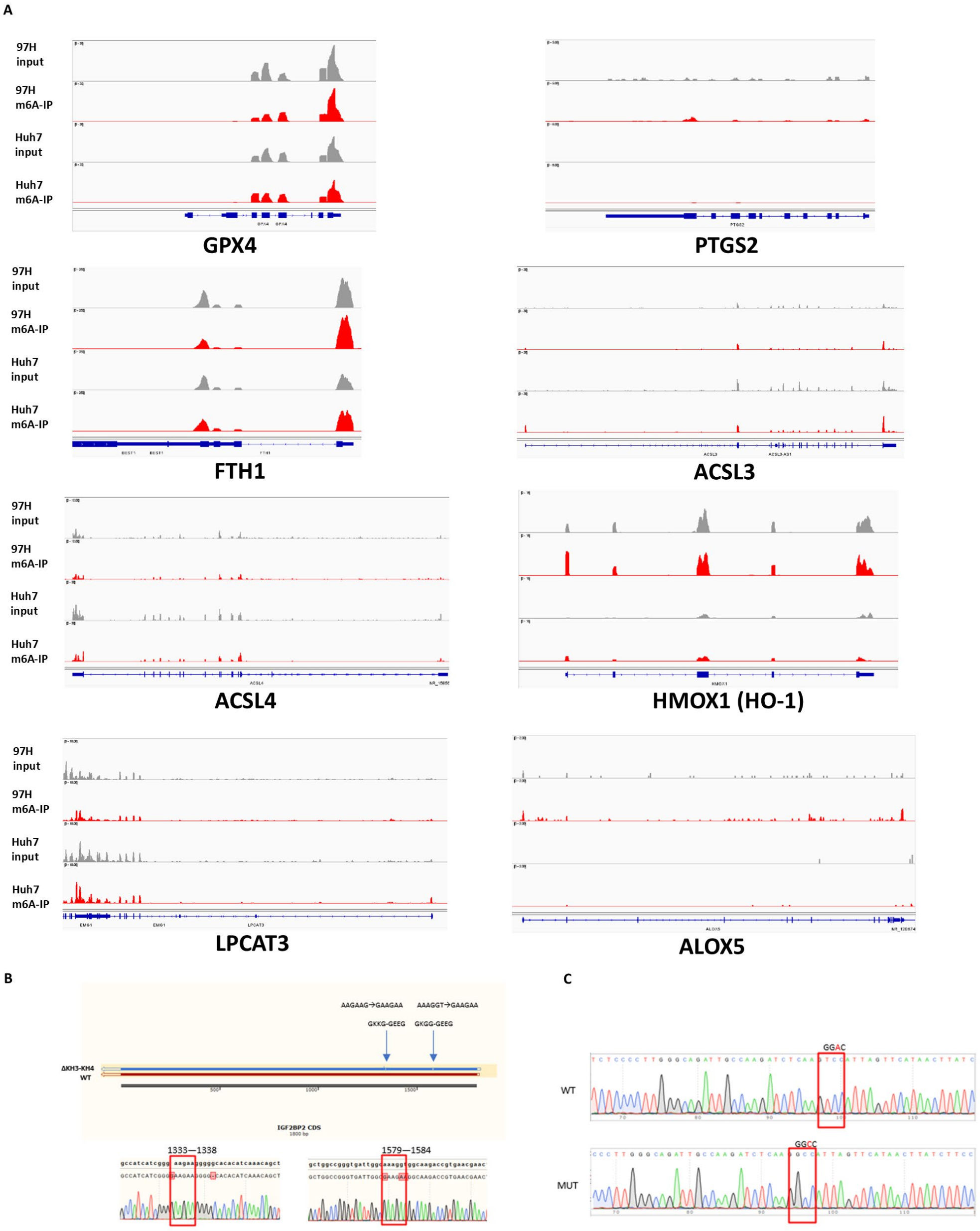
A
97H input
97H m6A-IP
Huh7 input
Huh7 m6A-IP
GPX4
PTGS2
FTH1
ACSL3
ACSL4
HMOX1 (HO-1)
LPCAT3
ALOX5
B
AAGAAG→GAAGAA
AAAGGT→GAAGAA
GKKG-GEEG
GKKG-GEEG
ΔKH3-KH4
WT
IGF2BP2 CDS
1333—1338
1579—1584
C
GGAC
WT
GGCC
MUT

Supplementary Fig 2. Screening of downstream target mRNAs of METTL3.

A: Genome Browser screenshots of MeRIP-seq read density signals on GPX4, PTGS2, FTH1, ACSL3, ACSL4, HMOX1, LPCAT3 and ALOX5 mRNA in MHCC-97H and Huh7 cells; B: WT and ΔKH3-KH4 IGF2BP2 plasmids sequencing comparison; C. SLC7A11 3'UTR WT and Mutant pMIR vector sequencing comparison.

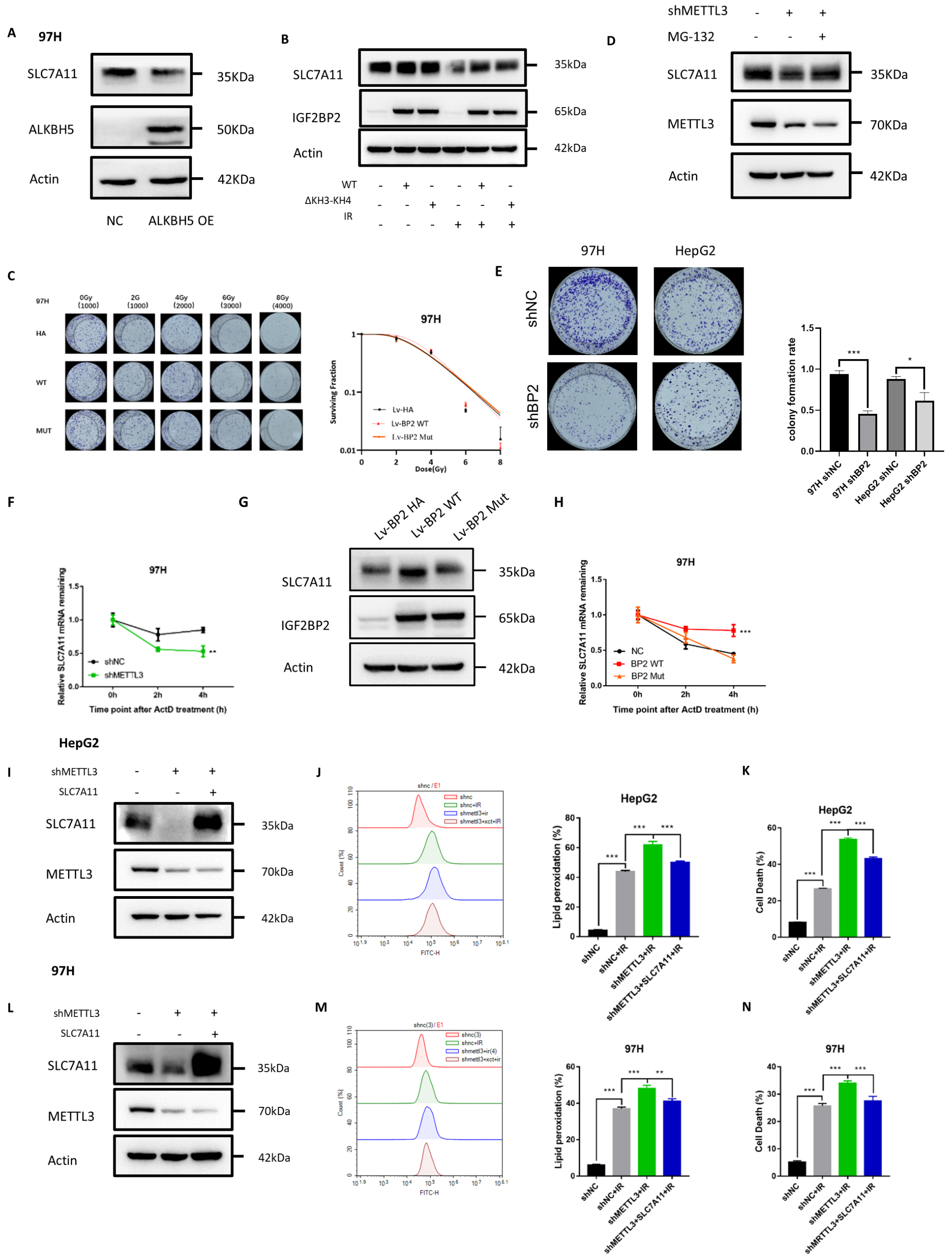
A
97H
SLC7A11
ALKBH5
Actin
35KDa
50KDa
42KDa
NC
ALKBH5 OE
B
SLC7A11
IGF2BP2
Actin
35kDa
65kDa
42kDa
WT
ΔKH3-KH4
IR
D
shMETTL3
MG-132
SLC7A11
METTL3
Actin
35KDa
70KDa
42KDa
C
97H
0Gy (1000)
2G (1000)
4Gy (2000)
6Gy (3000)
8Gy (4000)
HA
WT
MUT
Surviving Fraction
Dose(Gy)
Lv-HA
Lv-BP2 WT
Lv-BP2 Mut
E
97H
HepG2
shNC
shBP2
colony formation rate
97H shNC
97H shBP2
HepG2 shNC
HepG2 shBP2
F
97H
Relative SLC7A11 mRNA remaining
shNC
shMETTL3
Time point after ActD treatment (h)
G
Lv-BP2 HA
Lv-BP2 WT
Lv-BP2 Mut
SLC7A11
IGF2BP2
Actin
35kDa
65kDa
42kDa
H
97H
Relative SLC7A11 mRNA remaining
NC
BP2 WT
BP2 Mut
Time point after ActD treatment (h)
HepG2
I
shMETTL3
SLC7A11
SLC7A11
METTL3
Actin
35kDa
70kDa
42kDa
J
FITC-H
Count (%)
K
HepG2
Lipid peroxidation (%)
shNC
shNC+IR
shMETTL3+IR
shMETTL3+SLC7A11+IR
HepG2
Cell Death (%)
97H
L
shMETTL3
SLC7A11
SLC7A11
METTL3
Actin
35kDa
70kDa
42kDa
M
FITC-H
Count (%)
N
97H
Lipid peroxidation (%)
97H
Cell Death (%)

Supplementary Fig 3. METTL3 and IGF2BP2 contributed to the radioresistance by regulating the expression of SLC7A11.

A: Protein levels of ALKBH5 and SLC7A11 after ALKBH5 overexpression in MHCC-97H cells were detected by western blot; B: Protein levels of IGF2BP2 and SLC7A11 in lv-NC, lv-BP2 WT and lv-BP2 mut MHCC-97H cells treated with or without IR were detected by western blot; C. Representative images of colony formation and dose responses of survival fractions of lv-NC, lv-BP2 WT and lv-BP2 mut MHCC-97H cells after IR; D: Protein levels of SLC7A11 and METTL3 in shNC, shMETTL3 MHCC-97H cells treated with 10 μM MG-132 for 12h were detected by western blot; E: Cell proliferation abilities after knockdown of IGF2BP2 in MHCC-97H and HepG2 cells were detected by colony formation assay; F: Relative mRNA levels of SLC7A11 in shNC, shMETTL3 MHCC-97H cels treated with 5ug/ml actinomycin D at indicated time points; G: Protein levels of IGF2BP2 and SLC7A11 in MHCC-97H after overexpression of WT or ΔKH3-KH4 mutant IGF2BP2 were detected by western blot; H: Relative mRNA levels of SLC7A11 in NC, WT and Mutant IGF2BP2 overexpressed MHCC-97H cells treated with 5ug/ml actinomycin D at indicated time points I, L: Protein levels of METTL3 and SLC7A11 in shNC, shMETTL3 and shMETTL3+ SLC7A11 overexpression HepG2 (I) or MHCC-97H (L) cell lines were detected by western blot; J, M: Lipid peroxidation assessment in shNC, shMETTL3 and shMETTL3+ SLC7A11 overexpression HepG2 (J) or MHCC-97H (M) cell lines after exposure to IR, bar graphs showing relative levels of lipid peroxidation by C11-BODIPY staining in the indicated cells. Error bars are means ± SD, n = 3 independent repeats; K, N: Cell death rates of shNC, shMETTL3 and shMETTL3+ SLC7A11 overexpression HepG2 (K) or MHCC-97H (N) cell lines treated with IR were detected by Trypan blue staining; $^{*}P < 0.05$, $^{**}P < 0.01$, $^{***}P < 0.001$.

Supplementary Table 1. QPCR/ RT-PCR primers used in our study

Gene	Primer (5'-3')	Sequence
METTL3	Forward	TTGTCTCCAACCTTCCGTAGT
	Reverse	CCAGATCAGAGAGGTGGTGTAG
GAPDH	Forward	TCCAAAATCAAGTGGGGCGAT
	Reverse	GGGCAGAGATGATGACCCTTT
IGF2BP2	Forward	AGCCTGTCACCATCCATGC
	Reverse	CTTCGGCTAGTTTGGTCTCATC
SLC7A11	Forward	GCGTGGGCATGTCTCTGAC
	Reverse	GCTGGTAATGGACCAAAGACTTC
ALKBH5	Forward	AGTTCCAGTTCAAGCCTATTCG
	Reverse	TGAGCACAGTCACGCTTCC

Supplementary Table 2. MeRIP-qPCR/ RIP-qPCR primers used in our study

Gene	Primer (5'-3')	Sequence
MeRIP/RIP SLC7A11 site 1	Forward	TGGAACGAGGAGGTGGAGAAT
	Reverse	TCAGCGCTATAGTGTTCACAGG
MeRIP/RIP SLC7A11 site 2	Forward	TGGGACAAGAAACCCAGGTG
	Reverse	GTCTCCCCTTGGGCAGATTG
RIP SLC7A11 site 1	Forward	CCCTCCTCTCTGTTACTATTGG
	Reverse	TCAAGTACGCTGAAACACACAC
RIP SLC7A11 site 2	Forward	ATTGGTTAGGAGAACTGCTTGCT
	Reverse	CAGTCACCACTGCAAAAGAGA
RIP SLC7A11 site 3	Forward	TTCCTAGCACTGATGCCTGC
	Reverse	AGCCCATAAACACCATCTGGC

Supplementary Table 3. Primers for shRNA synthesized in our study

Primers	Sequences
METTL3-shRNA#1-Sense	5’-CCGGGCAAGTATGTTCACTATGAAACTCGAGTTTCATAGTGAACATACTTGCTTTTTG-3’
METTL3-shRNA#1-Antisense	5’-AATTCAAAAAGCAAGTATGTTCACTATGAAACTCGAGTTTCATAGTGAACATACTTGC -3’
METTL3-shRNA#2-Sense	5’-CCGGGCTGCACTTCAGACGAATTATCTCGAGATAATTCGTCTGAAGTGCAGCTTTTTG -3’
METTL3-shRNA#2-Antisense	5’-AATTCAAAAAGCTGCACTTCAGACGAATTATCTCGAGATAATTCGTCTGAAGTGCAGC-3’
METTL3-shRNA#3-Sense	5’-CCGGGCACATCCTACTCTTGTAACCCTCGAGGGTTACAAGAGTAGGATGTGCTTTTTG -3’
METTL3-shRNA#3-Antisense	5’-AATTCAAAAAGCACATCCTACTCTTGTAACCCTCGAGGGTTACAAGAGTAGGATGTGC-3’
IGF2BP2-shRNA#1-Sense	5’-CCGGTTCTTTCCGGGTTGTATATGCCTCGAGGCATATACAACCCGGAAAGAATTTTTG-3’
IGF2BP2-shRNA#1-Antisense	5’-AATTCAAAAATTCTTTCCGGGTTGTATATGCCTCGAGGCATATACAACCCGGAAAGAA -3’
IGF2BP2-shRNA#2-Sense	5’-CCGGTTTCAGTTTCCCAAAGATCCGCTCGAGCGGATCTTTGGGAAACTGAAATTTTTG -3’
IGF2BP2-shRNA#2-Antisense	5’-AATTCAAAAATTTCAGTTTCCCAAAGATCCGCTCGAGCGGATCTTTGGGAAACTGAAA-3’
IGF2BP2-shRNA#3-Sense	5’-CCGGAGTGAAGCTGGAAGCGCATATCTCGAGATATGCGCTTCCAGCTTCACTTTTTG -3’
IGF2BP2-shRNA#3-Antisense	5’-AATTCAAAAAAGTGAAGCTGGAAGCGCATATCTCGAGATATGCGCTTCCAGCTTCACT-3’
