# Supplementary material for: METTL3 inhibition promotes radiosensitivity in hepatocellular carcinoma through regulation of SLC7A11 expression
Source: Cell Death Dis. 2025 Jan 11;16(1):9. doi: 10.1038/s41419-024-07317-x (PMC11724875; doi:10.1038/s41419-024-07317-x)
Supplement: Supplementary file 2 — Original western blots [file 41419_2024_7317_MOESM2_ESM.pdf]

Figure 1F

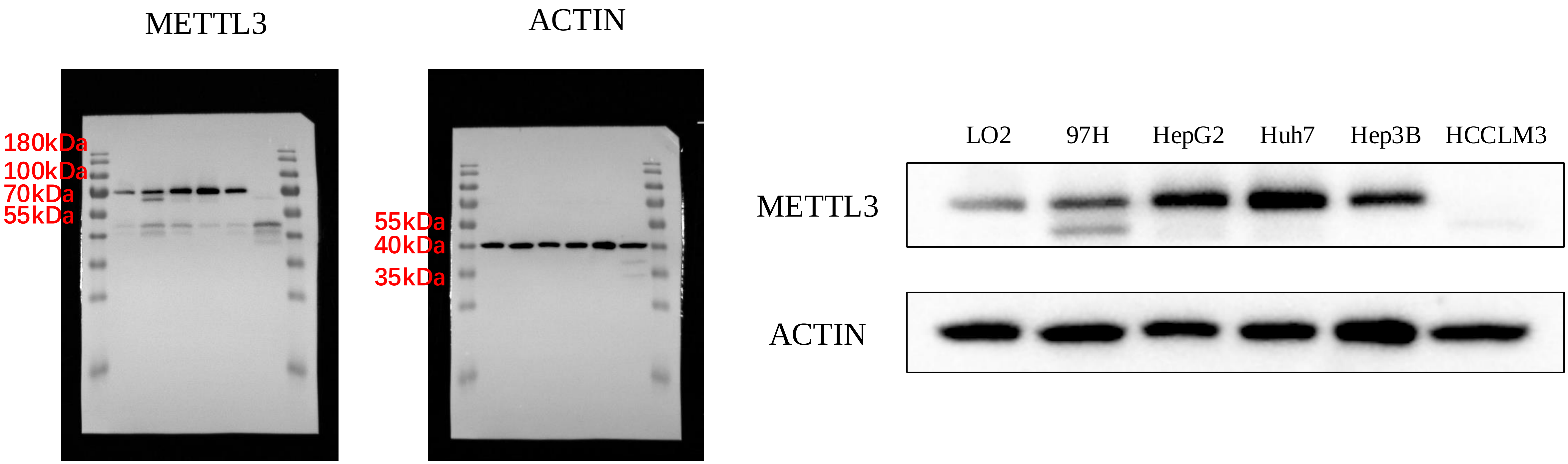
METTL3
ACTIN
180kDa
100kDa
70kDa
55kDa
55kDa
40kDa
35kDa
LO2
97H
HepG2
Huh7
Hep3B
HCCLM3
METTL3
ACTIN

Figure 1H

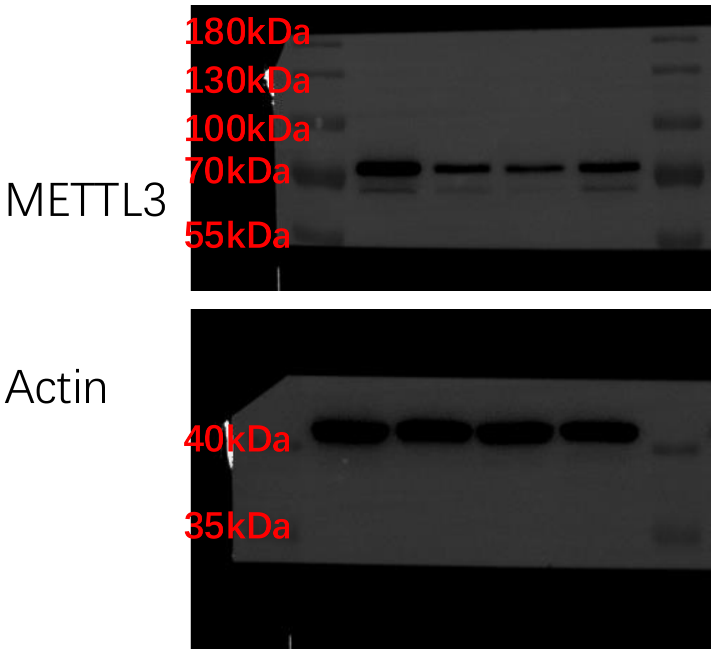
METTL3
Actin
180kDa
130kDa
100kDa
70kDa
55kDa
40kDa
35kDa

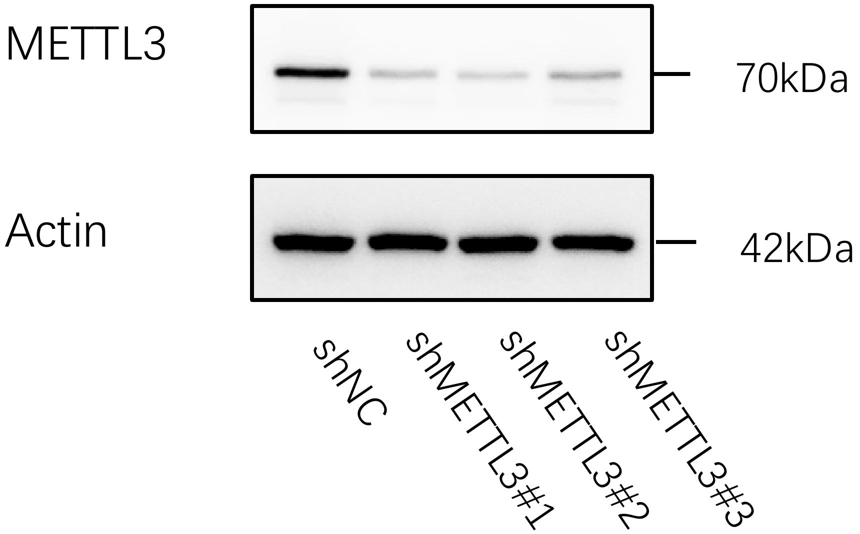
METTL3
Actin
70kDa
42kDa
shNC
shMETTL3#1
shMETTL3#2
shMETTL3#3

Figure 1J

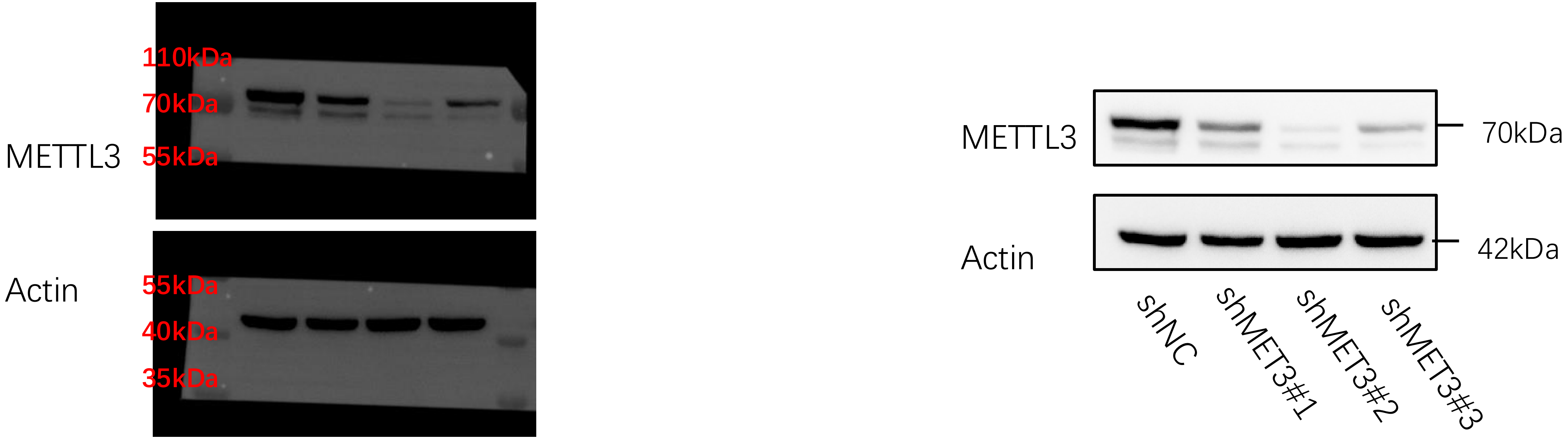
METTL3
110kDa
70kDa
55kDa
Actin
55kDa
40kDa
35kDa
METTL3
70kDa
Actin
42kDa
shNC
shMET3#1
shMET3#2
shMET3#3

Fig 2A

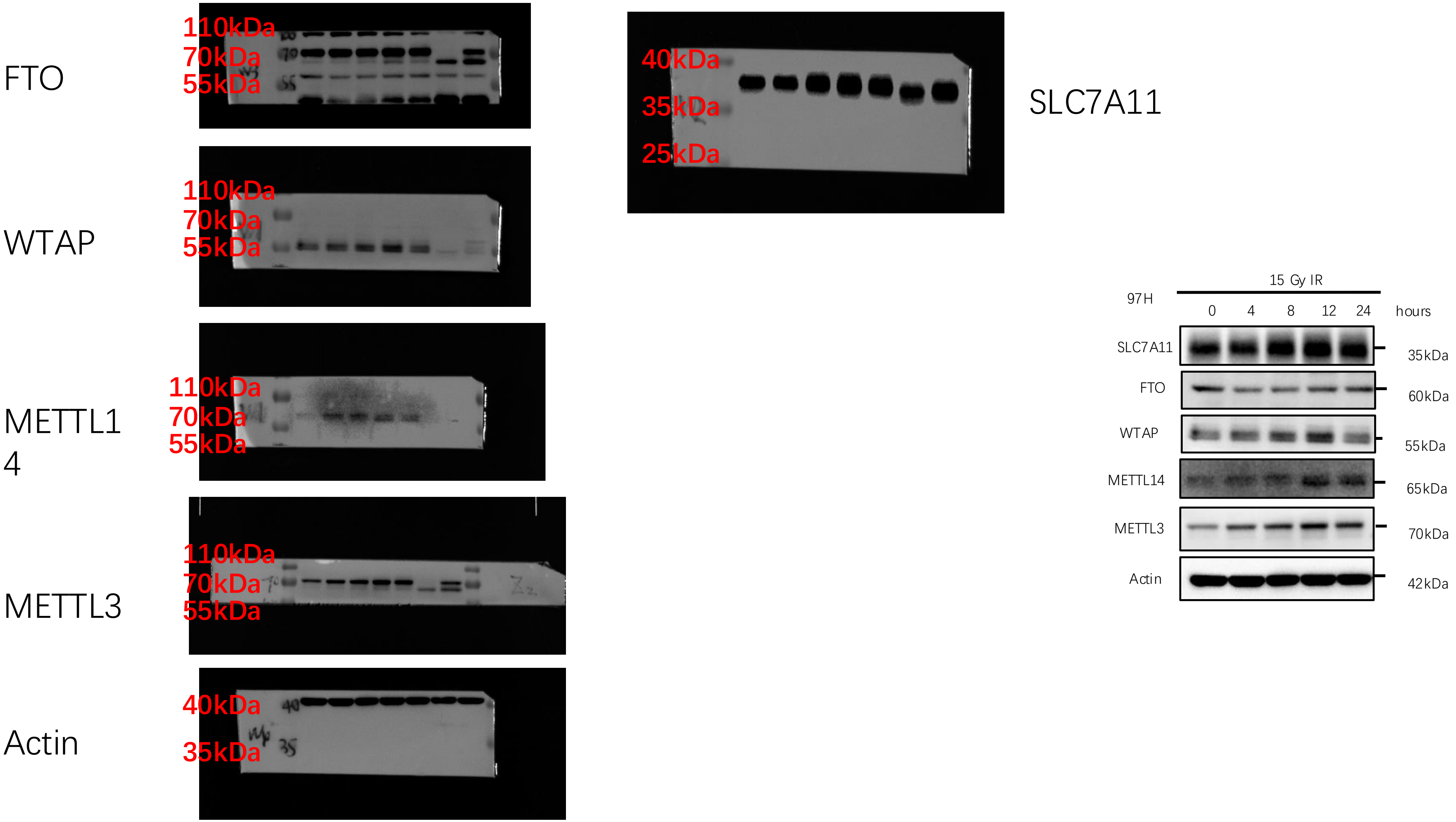

FTO
110kDa
70kDa
55kDa
WTAP
110kDa
70kDa
55kDa
METTL1
4
110kDa
70kDa
55kDa
METTL3
110kDa
70kDa
55kDa
Actin
40kDa
35kDa
40kDa
35kDa
25kDa
SLC7A11
97H
15 Gy IR
0
4
8
12
24
hours
SLC7A11
35kDa
FTO
60kDa
WTAP
55kDa
METTL14
65kDa
METTL3
70kDa
Actin
42kDa

Fig 3G

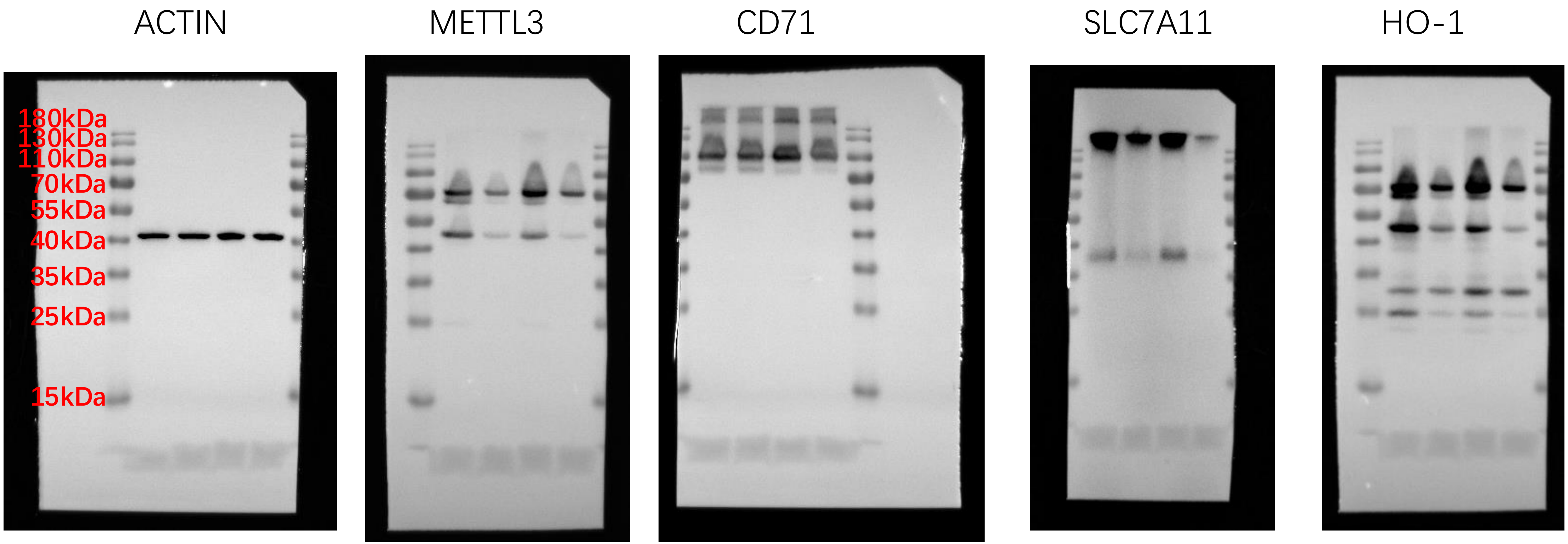
ACTIN
METTL3
CD71
SLC7A11
HO-1
180kDa
130kDa
110kDa
70kDa
55kDa
40kDa
35kDa
25kDa
15kDa

Fig 3G

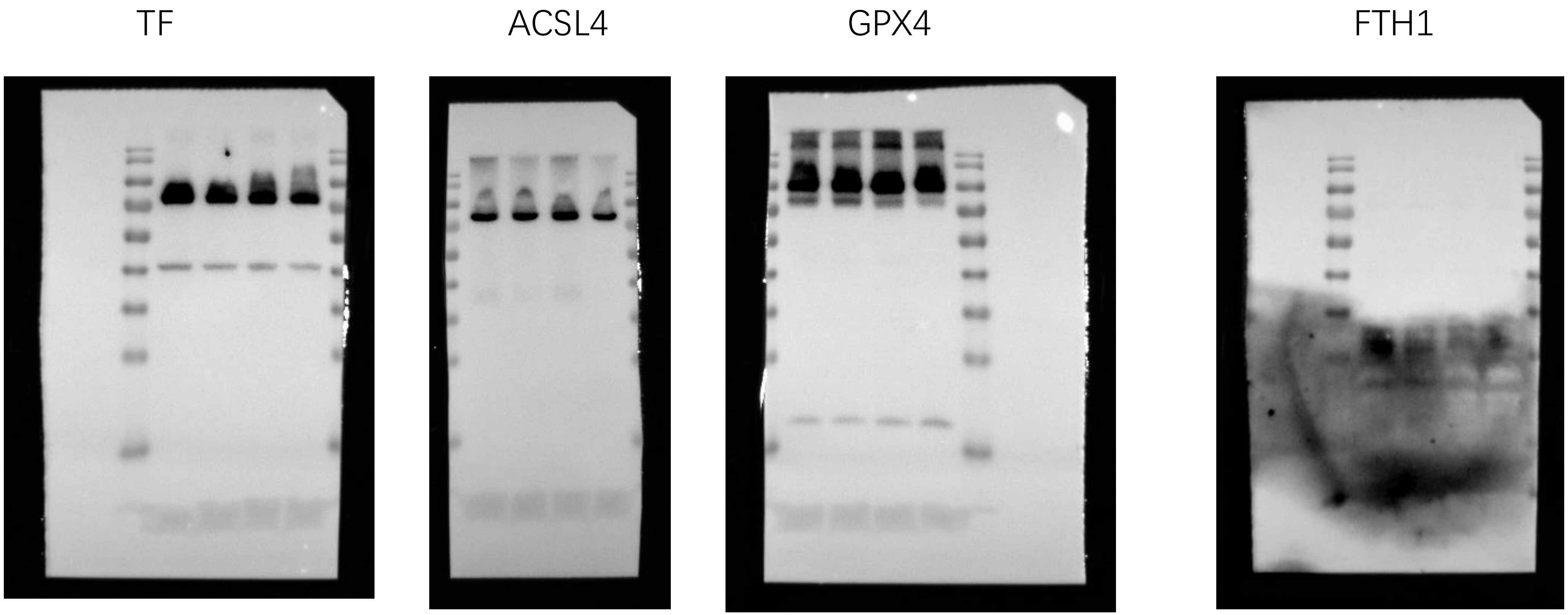

Fig 3G

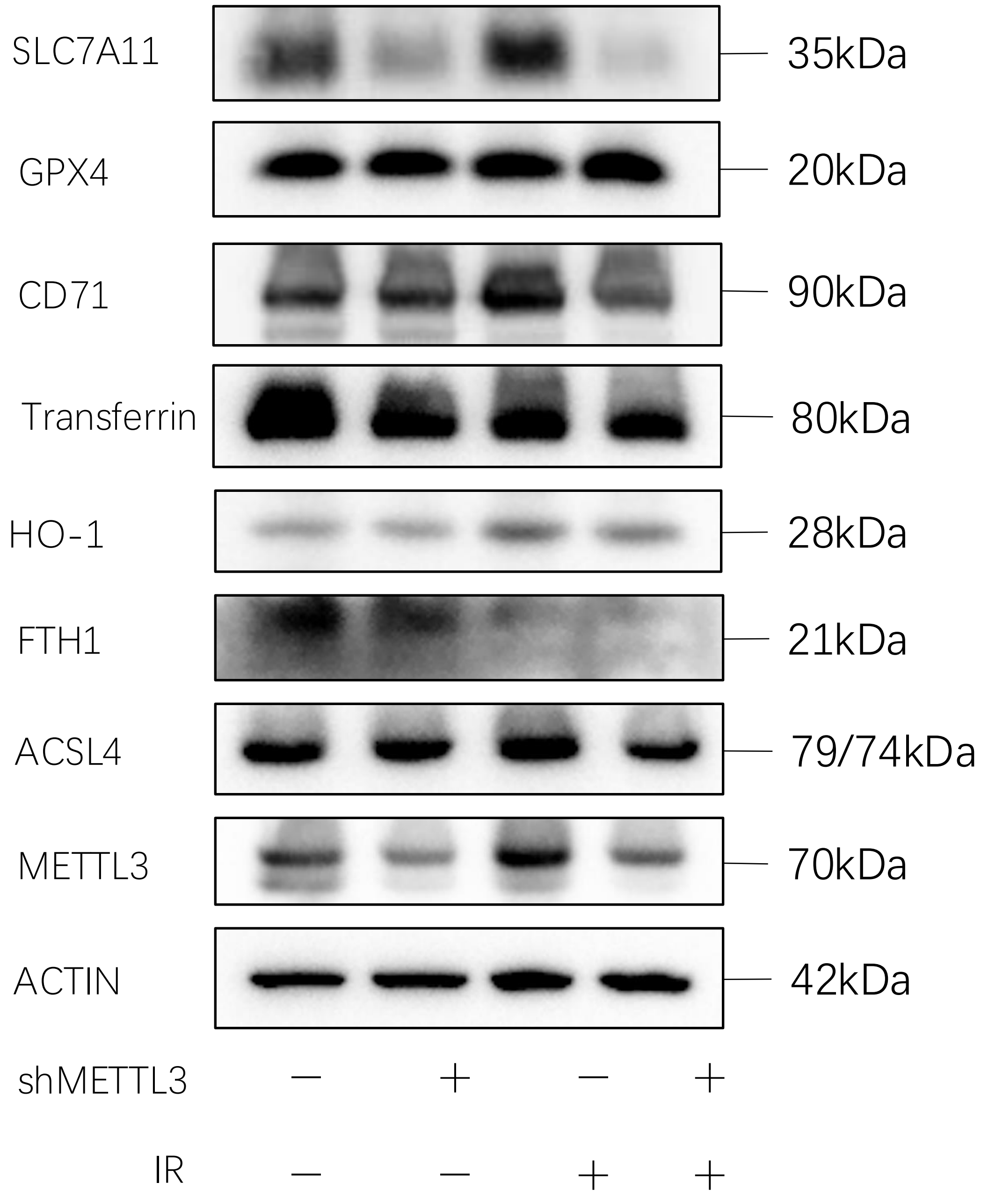
SLC7A11
35kDa
GPX4
20kDa
CD71
90kDa
Transferrin
80kDa
HO-1
28kDa
FTH1
21kDa
ACSL4
79/74kDa
METTL3
70kDa
ACTIN
42kDa
shMETTL3 − + − +
IR − − + +

Figure 4I

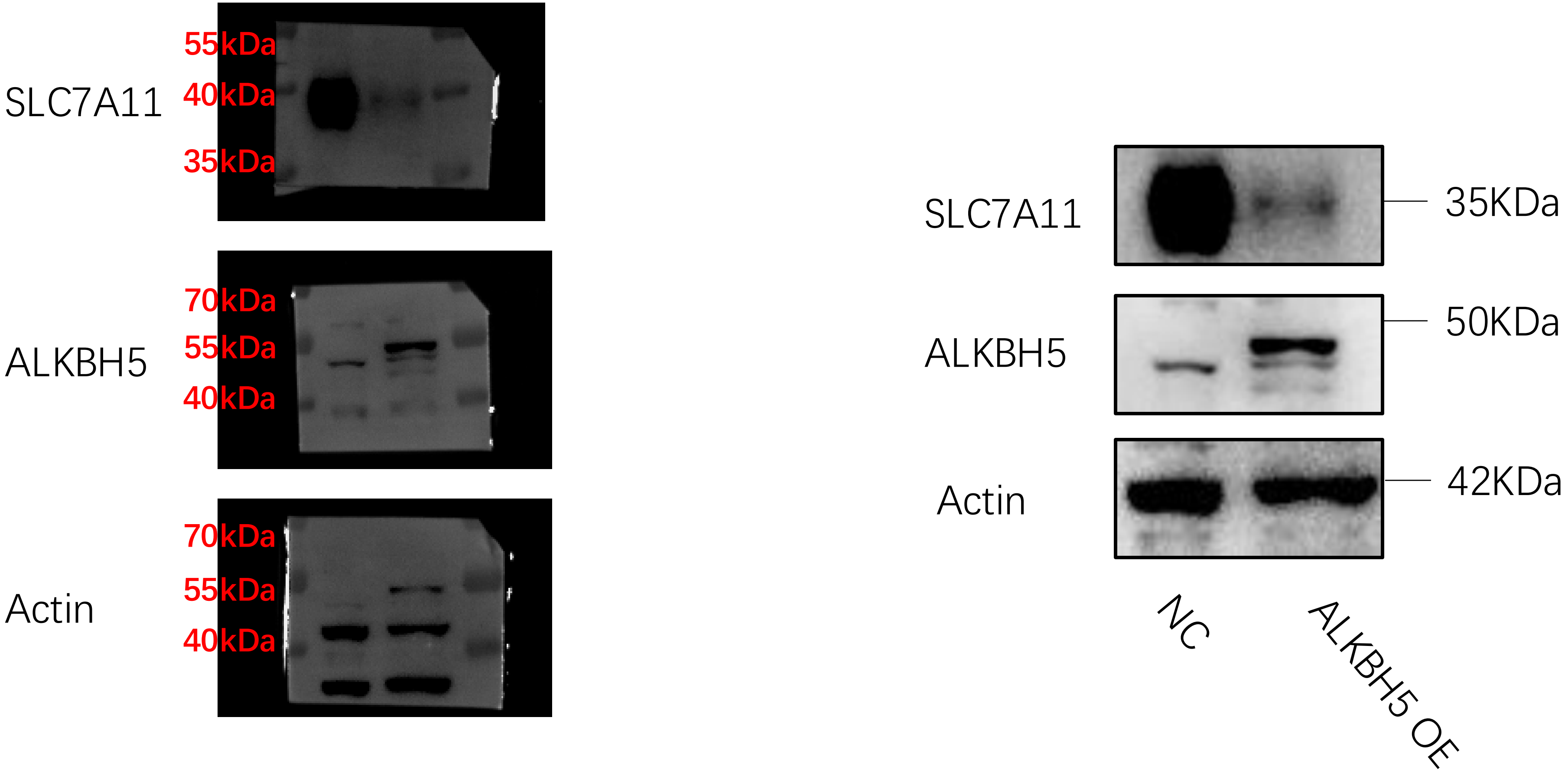
SLC7A11
55kDa
40kDa
35kDa
ALKBH5
70kDa
55kDa
40kDa
Actin
70kDa
55kDa
40kDa
SLC7A11
35KDa
ALKBH5
50KDa
Actin
42KDa
NC
ALKBH5 OE

Figure 5B

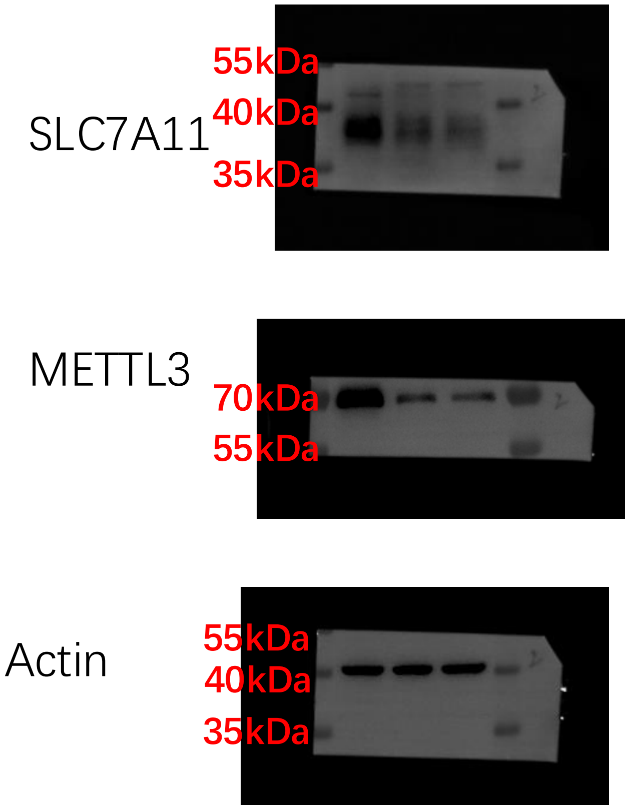
SLC7A11
55kDa
40kDa
35kDa
METTL3
70kDa
55kDa
Actin
55kDa
40kDa
35kDa

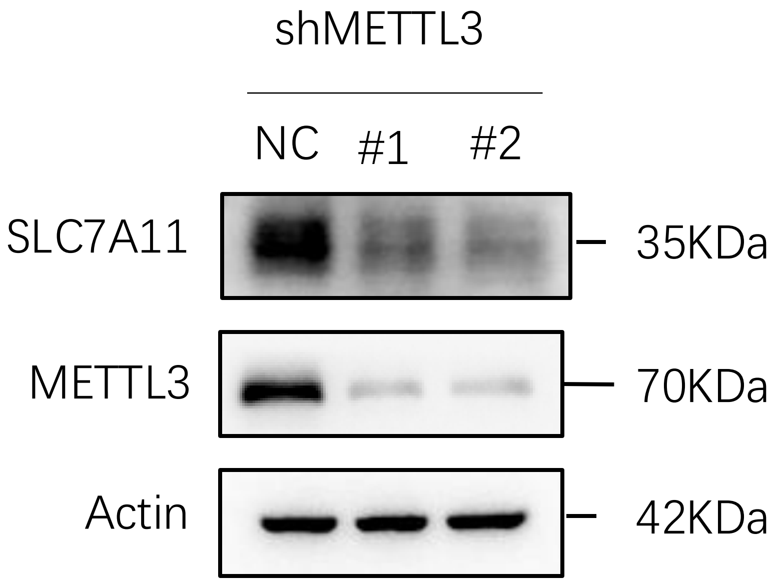
shMETTL3
NC
#1
#2
SLC7A11
35KDa
METTL3
70KDa
Actin
42KDa

Figure 5D

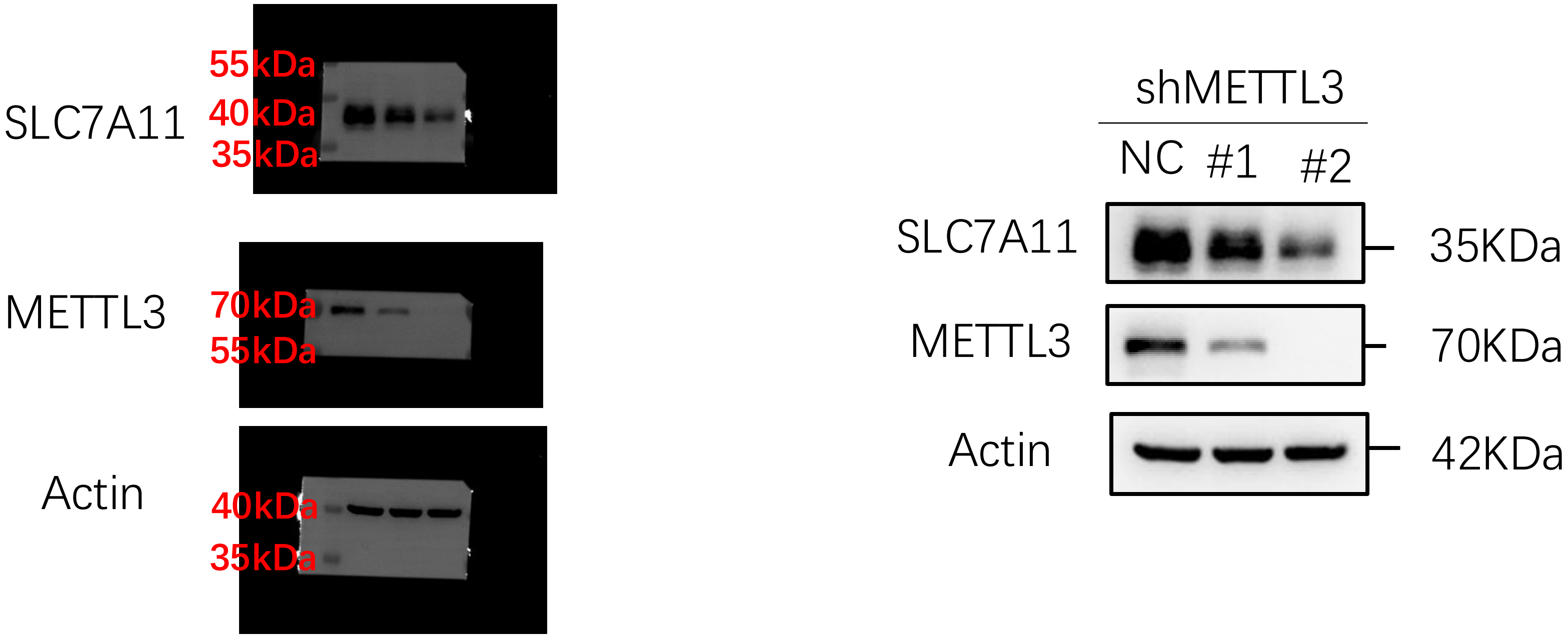
SLC7A11
55kDa
40kDa
35kDa
METTL3
70kDa
55kDa
Actin
40kDa
35kDa
shMETTL3
NC
#1
#2
SLC7A11
35KDa
METTL3
70KDa
Actin
42KDa

Figure 5J

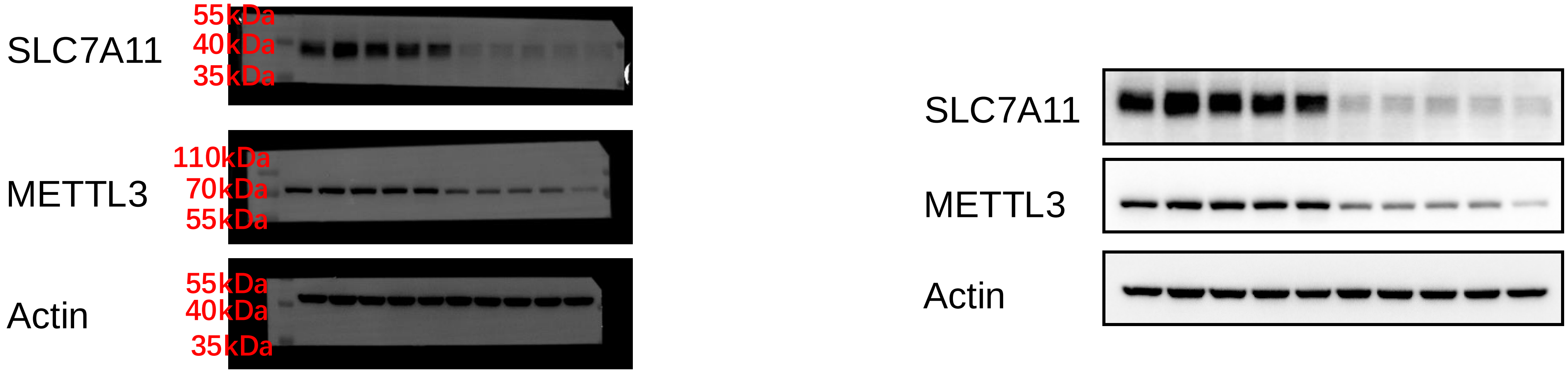
SLC7A11
55kDa
40kDa
35kDa
METTL3
110kDa
70kDa
55kDa
Actin
55kDa
40kDa
35kDa
SLC7A11
METTL3
Actin

Figure 5K

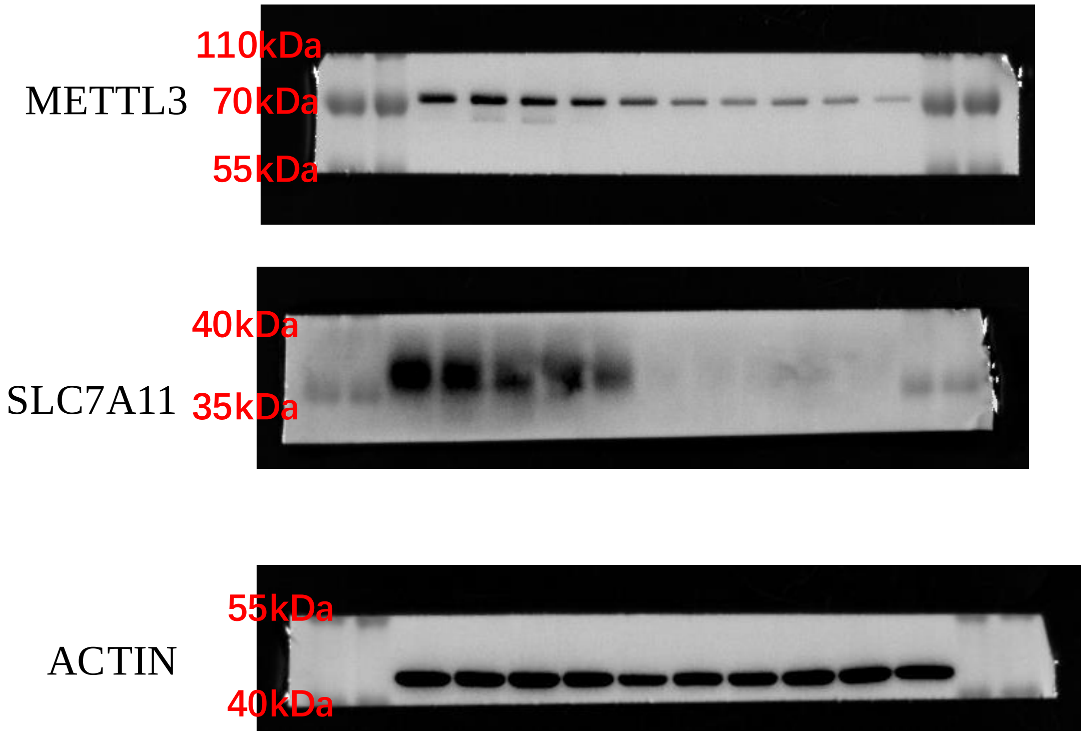

METTL3
110kDa
70kDa
55kDa
SLC7A11
40kDa
35kDa
ACTIN
55kDa
40kDa

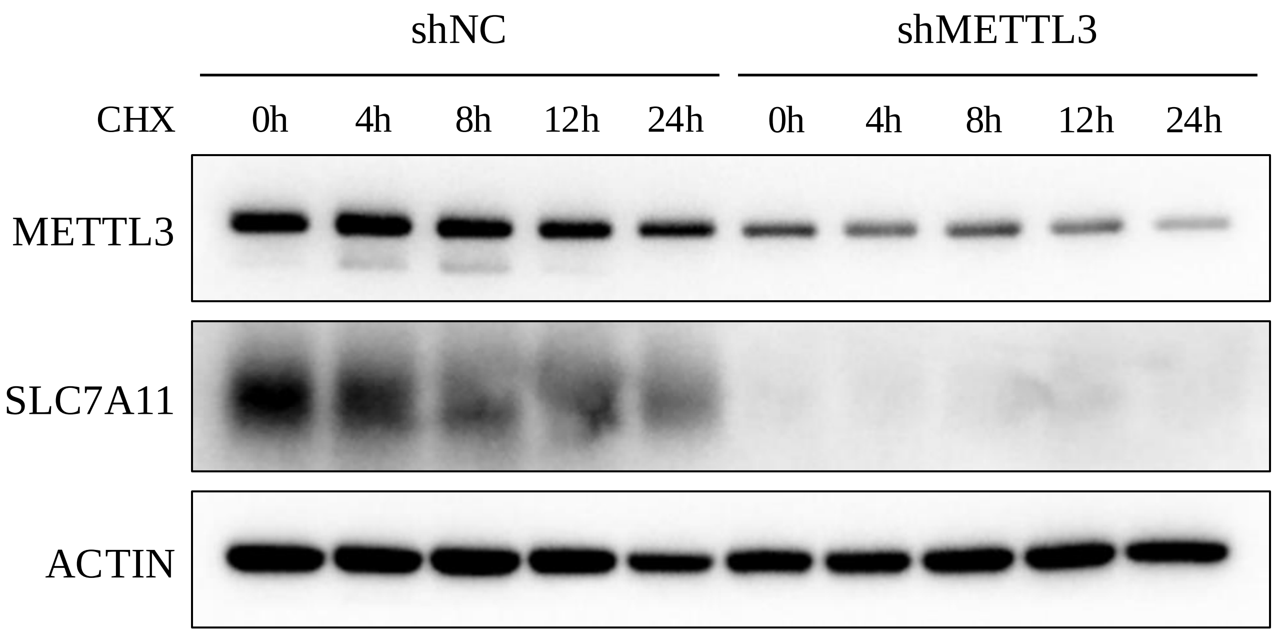

shNC
shMETTL3
CHX
0h
4h
8h
12h
24h
0h
4h
8h
12h
24h
METTL3
SLC7A11
ACTIN

Figure 5L

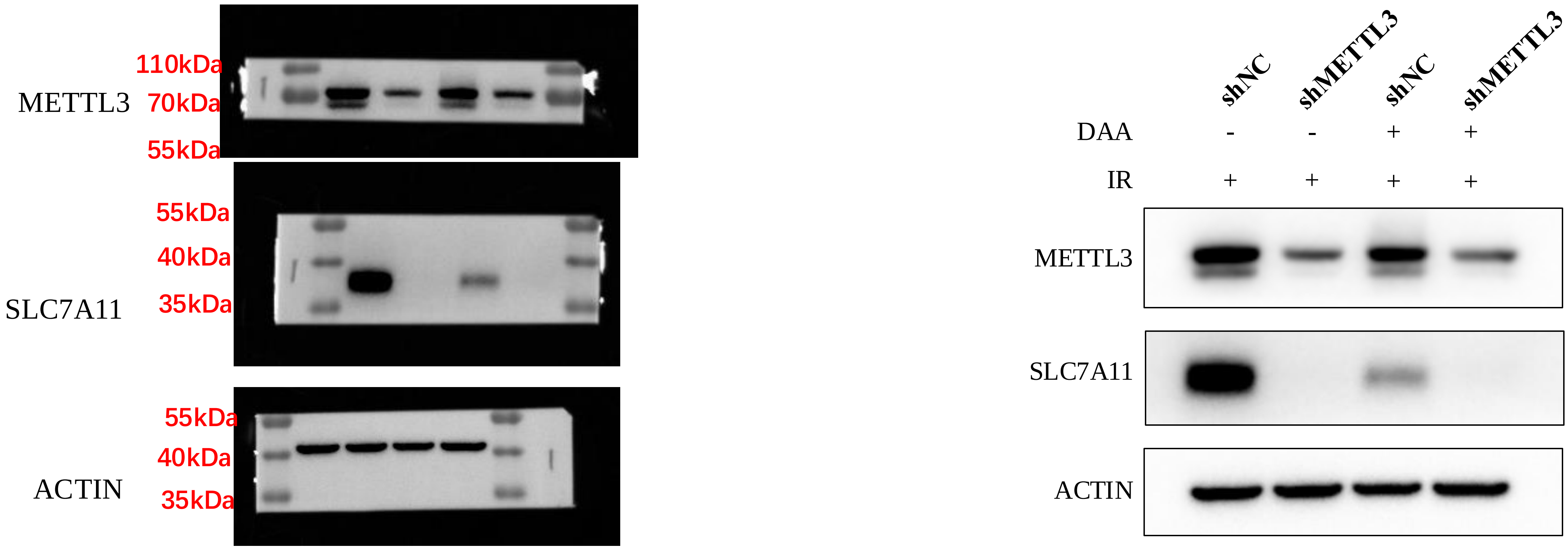
METTL3
110kDa
70kDa
55kDa
SLC7A11
55kDa
40kDa
35kDa
ACTIN
55kDa
40kDa
35kDa
shNC
shMETTL3
shNC
shMETTL3
DAA - - + +
IR + + + +
METTL3
SLC7A11
ACTIN

Figure 6I

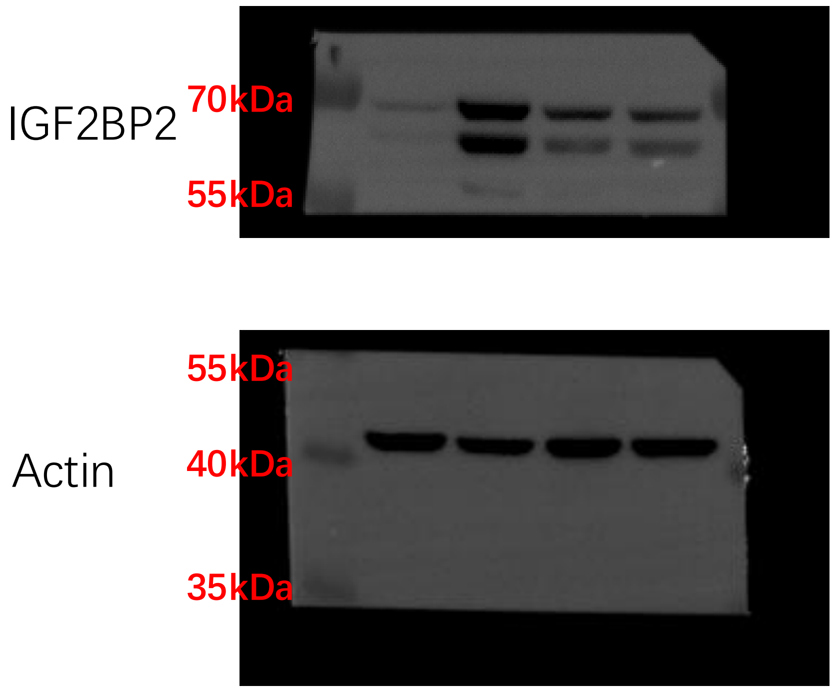

IGF2BP2
70kDa
55kDa
Actin
55kDa
40kDa
35kDa

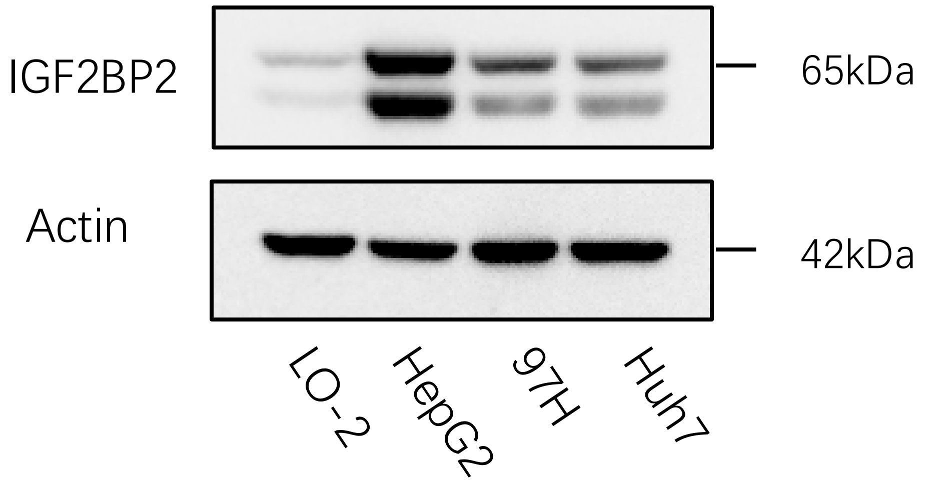

IGF2BP2
65kDa
Actin
42kDa
LO-2
HepG2
97H
Huh7

Figure 7B

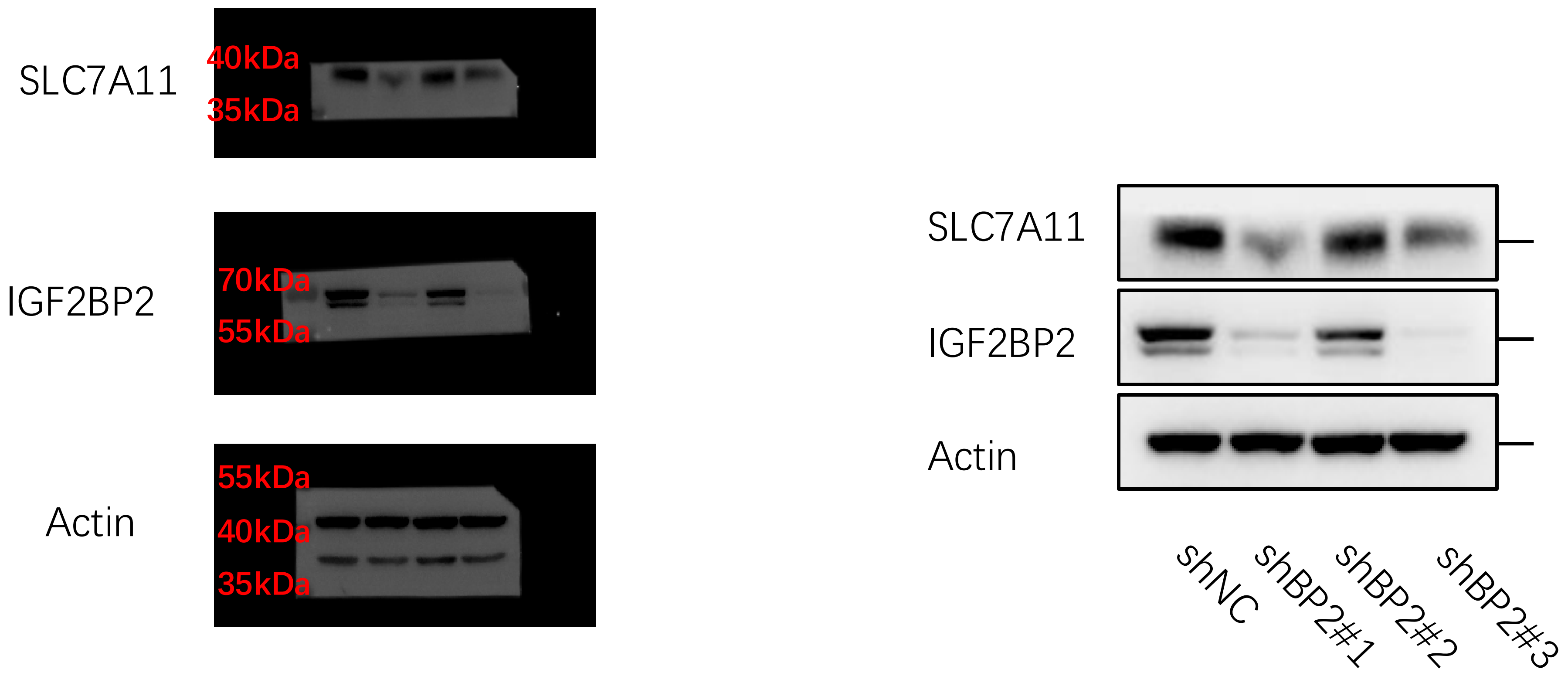

SLC7A11
40kDa
35kDa
IGF2BP2
70kDa
55kDa
Actin
55kDa
40kDa
35kDa
SLC7A11
35kDa
IGF2BP2
65kDa
Actin
42kDa
shNC
shBP2#1
shBP2#2
shBP2#3

Figure 7G

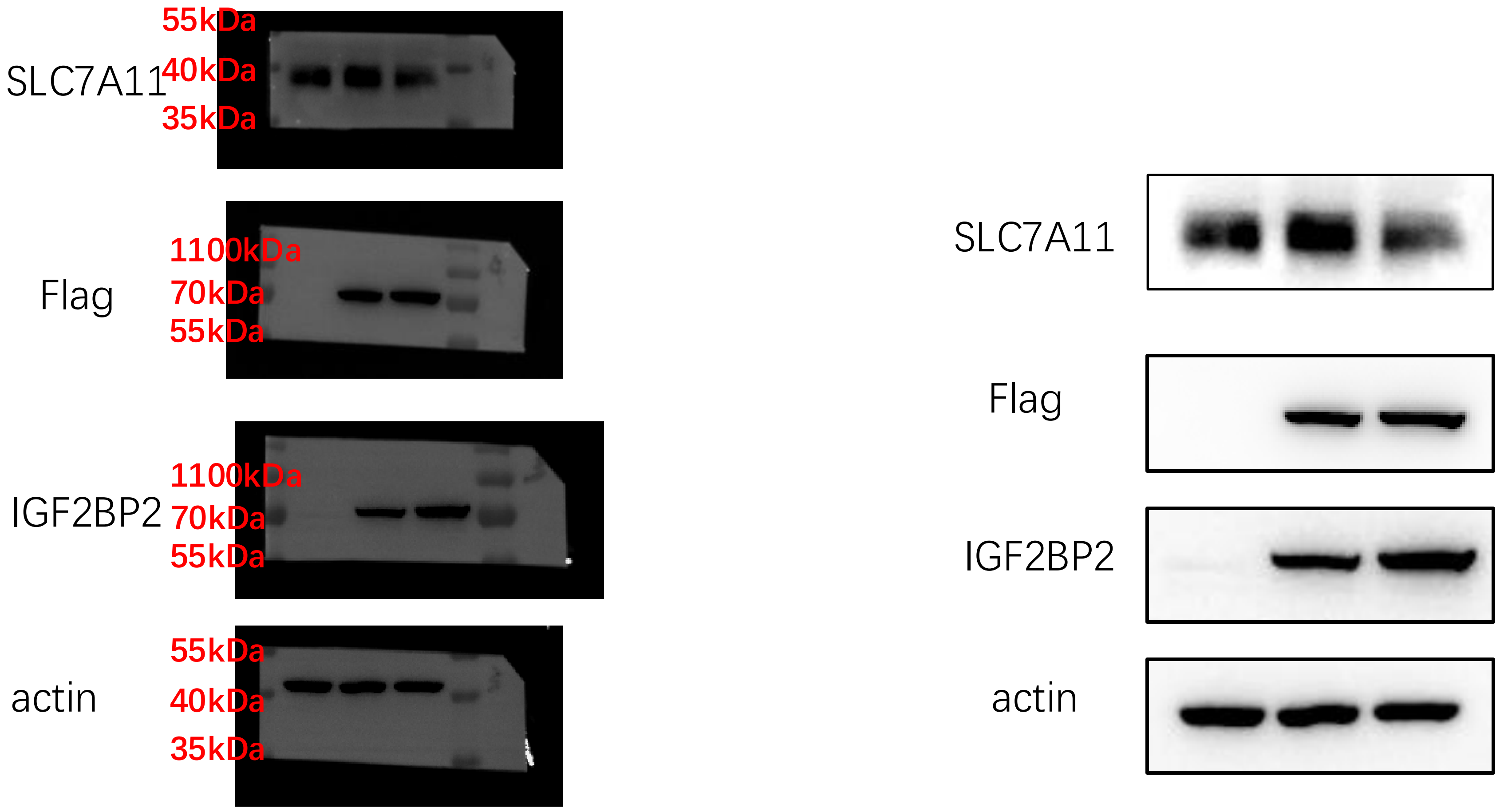
SLC7A11
55kDa
40kDa
35kDa
Flag
1100kDa
70kDa
55kDa
IGF2BP2
1100kDa
70kDa
55kDa
actin
55kDa
40kDa
35kDa
SLC7A11
Flag
IGF2BP2
actin

Fig 7K

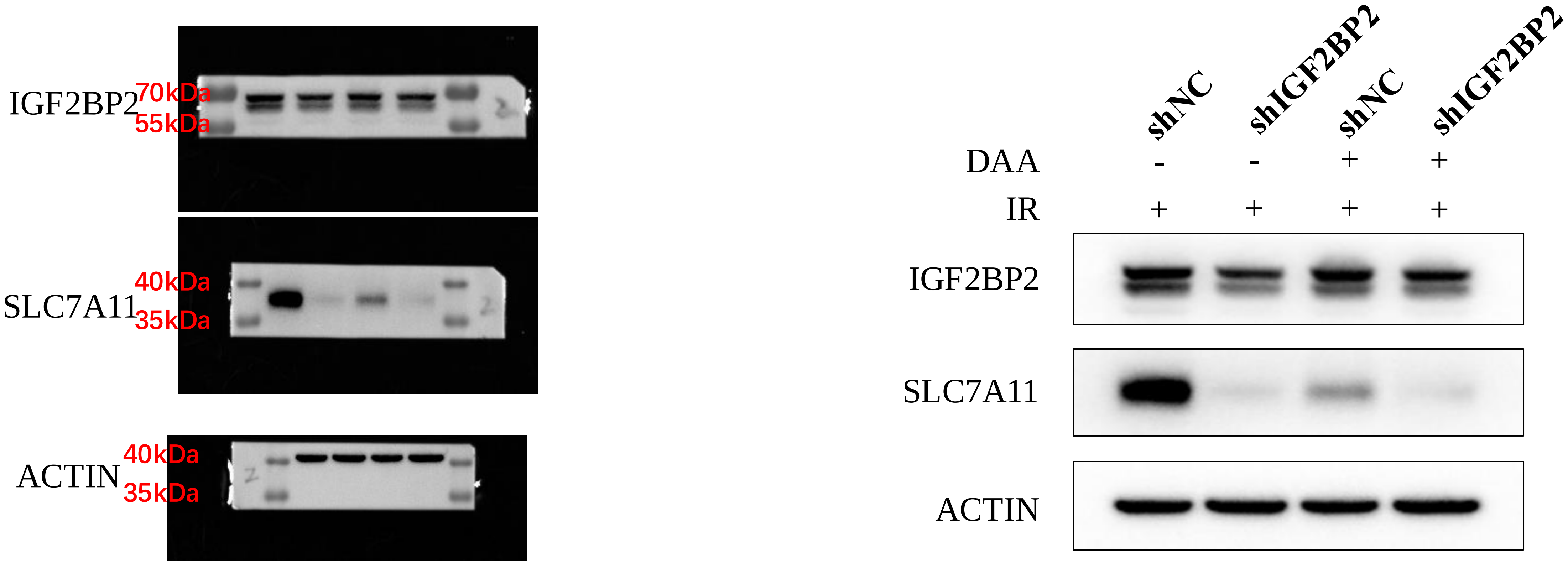
IGF2BP2
70kDa
55kDa
SLC7A11
40kDa
35kDa
ACTIN
40kDa
35kDa
shNC
shIGF2BP2
shNC
shIGF2BP2
DAA
-
-
+
+
IR
+
+
+
+
IGF2BP2
SLC7A11
ACTIN

Fig 8F

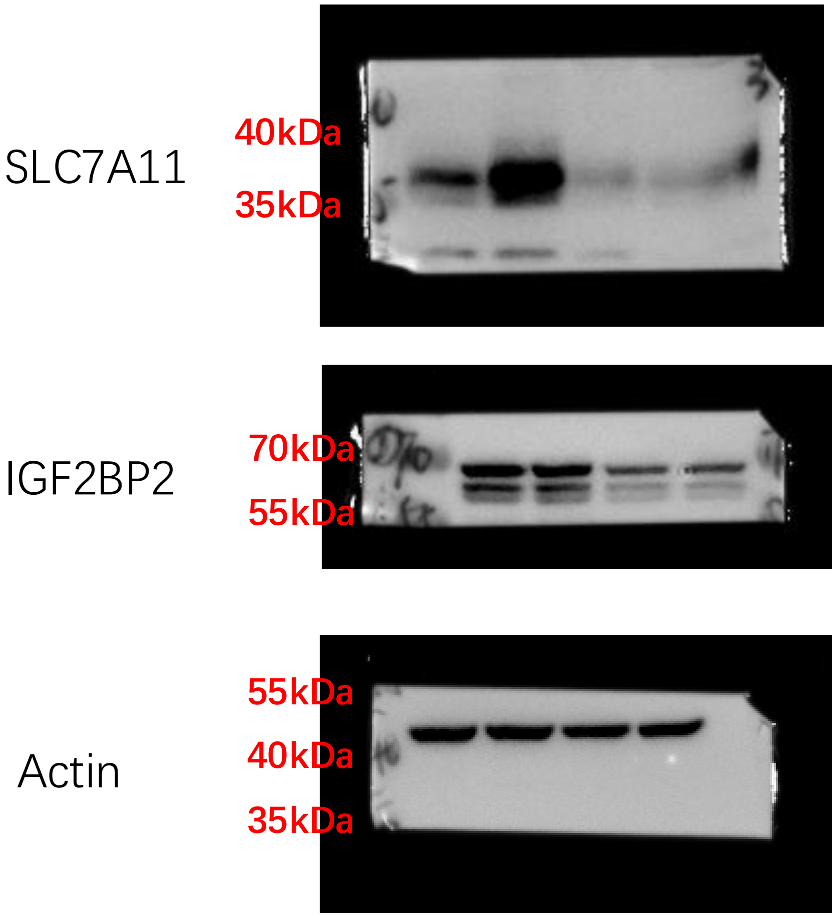
SLC7A11
40kDa
35kDa
IGF2BP2
70kDa
55kDa
Actin
55kDa
40kDa
35kDa

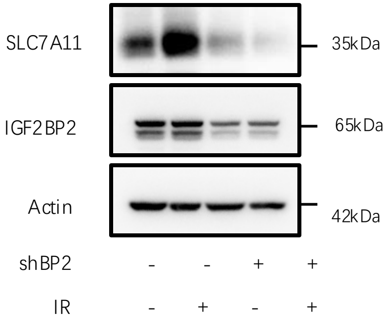
SLC7A11
35kDa
IGF2BP2
65kDa
Actin
42kDa
shBP2 - - + +
IR - + - +

Fig 8G

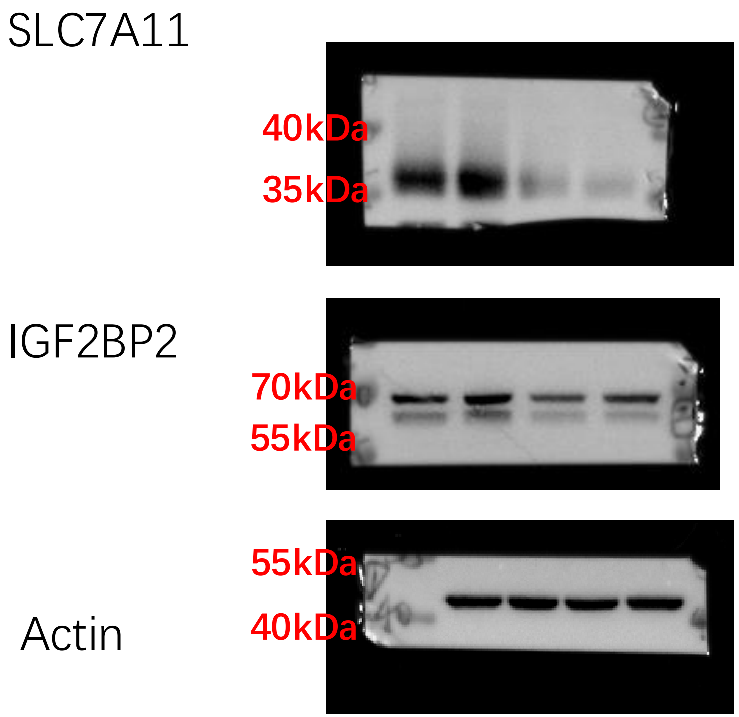
SLC7A11
40kDa
35kDa
IGF2BP2
70kDa
55kDa
Actin
55kDa
40kDa

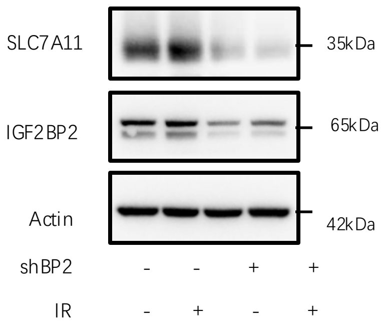
SLC7A11
35kDa
IGF2BP2
65kDa
Actin
42kDa
shBP2 - - + +
IR - + - +

Figure 8K

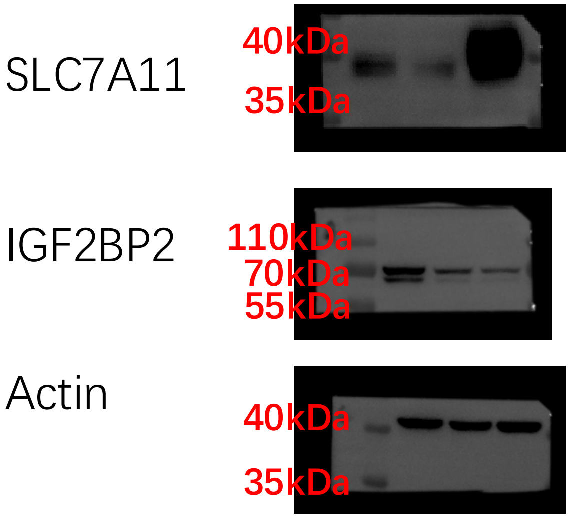
SLC7A11
40kDa
35kDa
IGF2BP2
110kDa
70kDa
55kDa
Actin
40kDa
35kDa

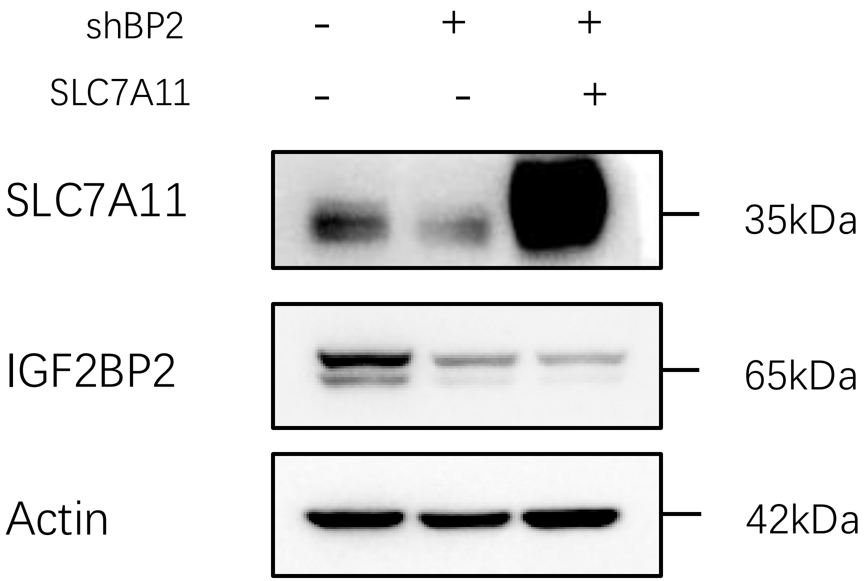
shBP2 - + +
SLC7A11 - - +
SLC7A11
35kDa
IGF2BP2
65kDa
Actin
42kDa

Figure 8N

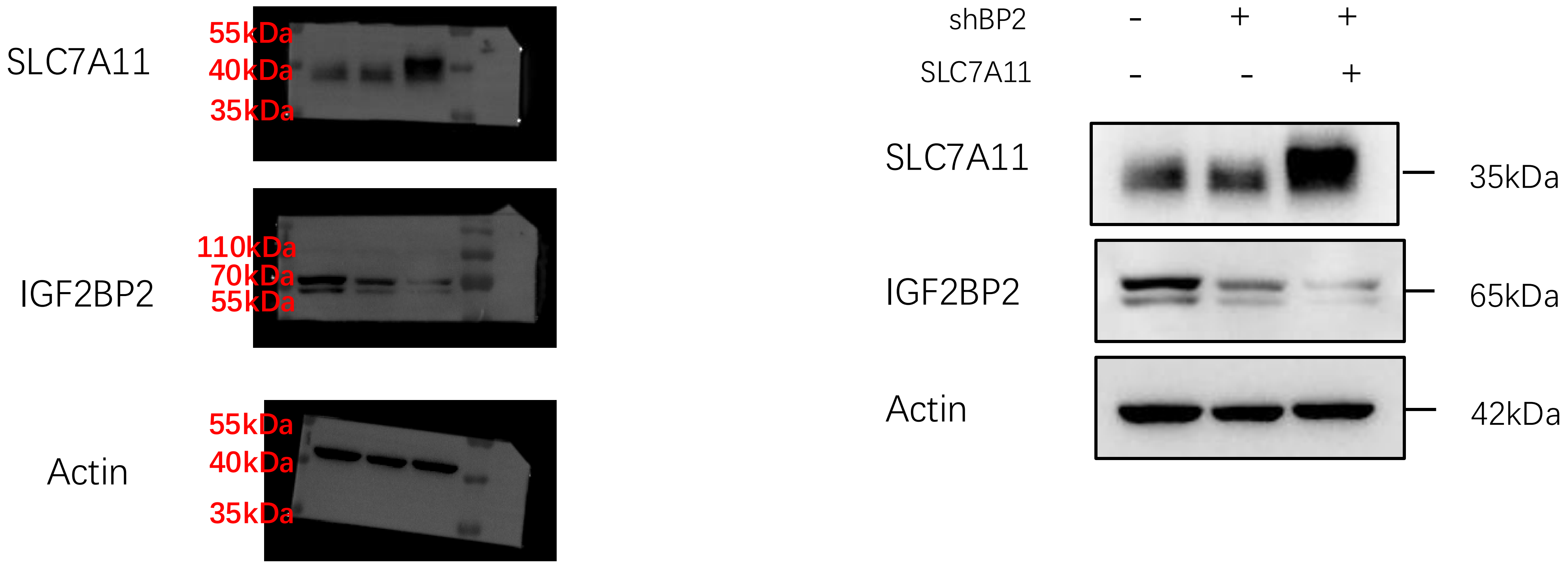
SLC7A11
55kDa
40kDa
35kDa
IGF2BP2
110kDa
70kDa
55kDa
Actin
55kDa
40kDa
35kDa
shBP2 - + +
SLC7A11 - - +
SLC7A11
35kDa
IGF2BP2
65kDa
Actin
42kDa

Fig 9F

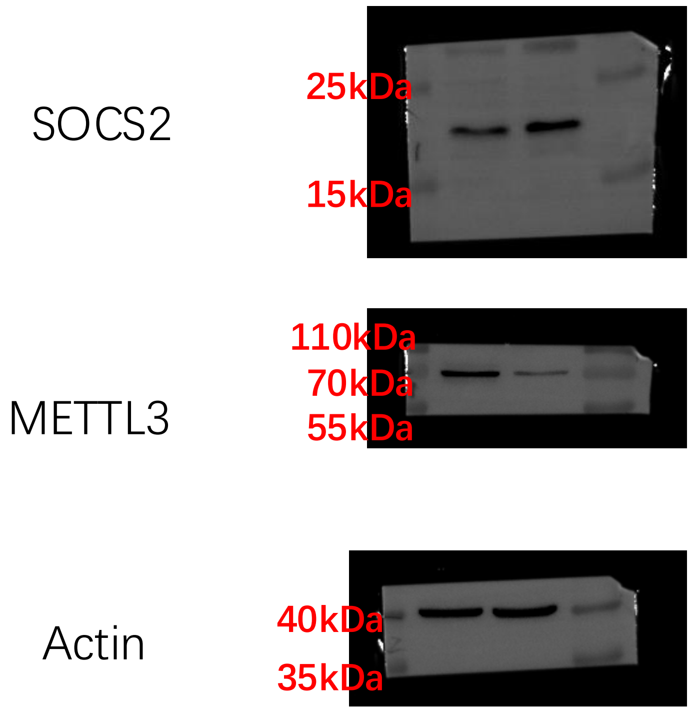
SOCS2
25kDa
15kDa
METTL3
110kDa
70kDa
55kDa
Actin
40kDa
35kDa

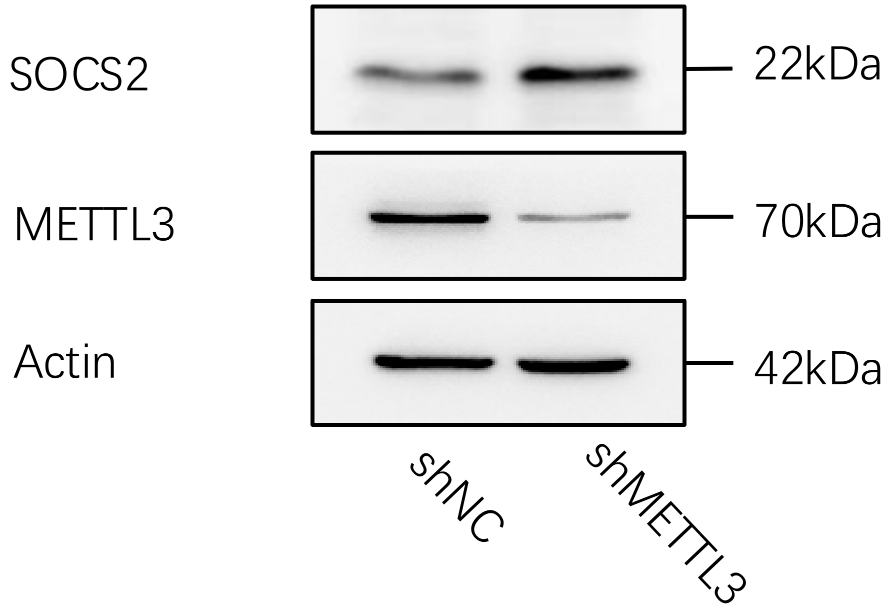
SOCS2
22kDa
METTL3
70kDa
Actin
42kDa
shNC
shMETTL3

Fig 9G

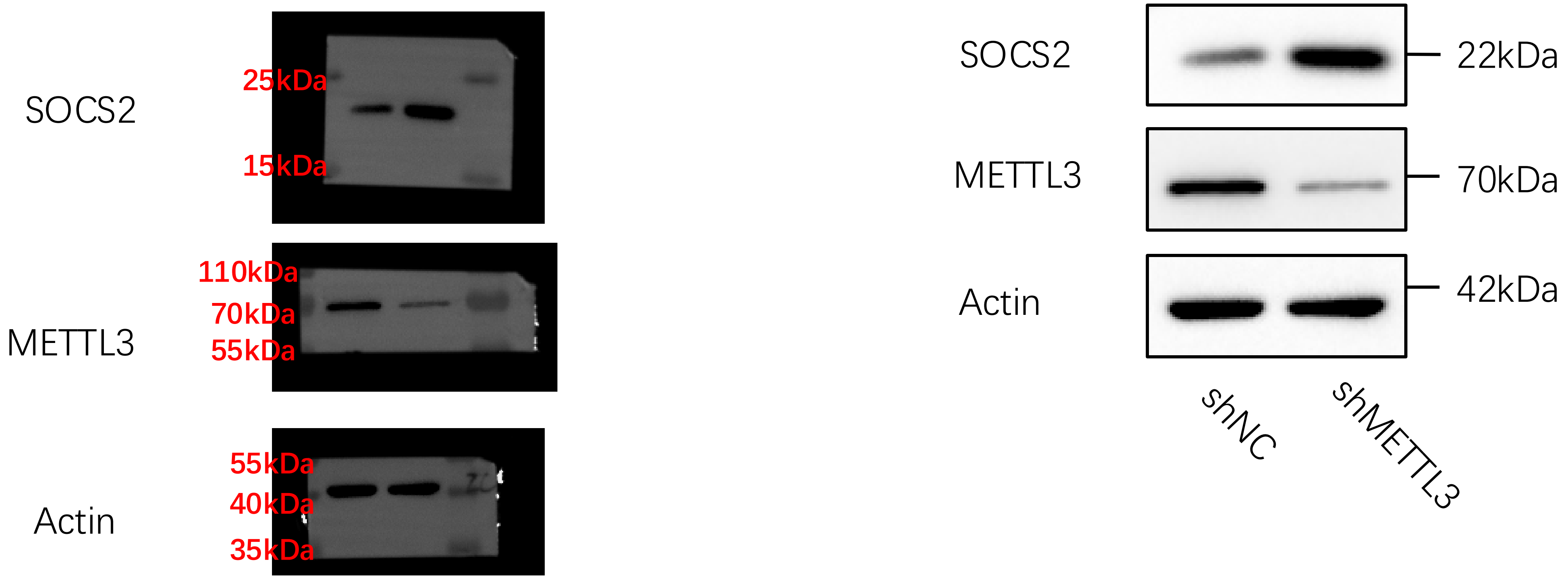
SOCS2
25kDa
15kDa
METTL3
110kDa
70kDa
55kDa
Actin
55kDa
40kDa
35kDa
SOCS2
22kDa
METTL3
70kDa
Actin
42kDa
shNC
shMETTL3

Figure 9H

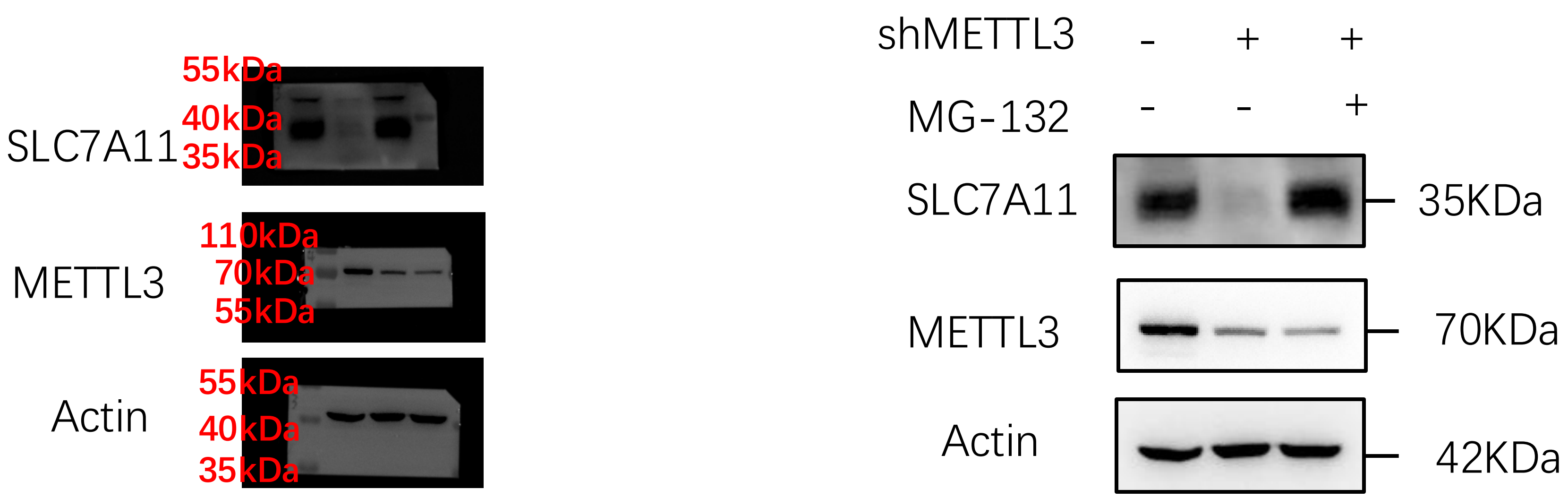

SLC7A11
55kDa
40kDa
35kDa
METTL3
110kDa
70kDa
55kDa
Actin
55kDa
40kDa
35kDa
shMETTL3 - + +
MG-132 - - +
SLC7A11
35KDa
METTL3
70KDa
Actin
42KDa

Figure 9I

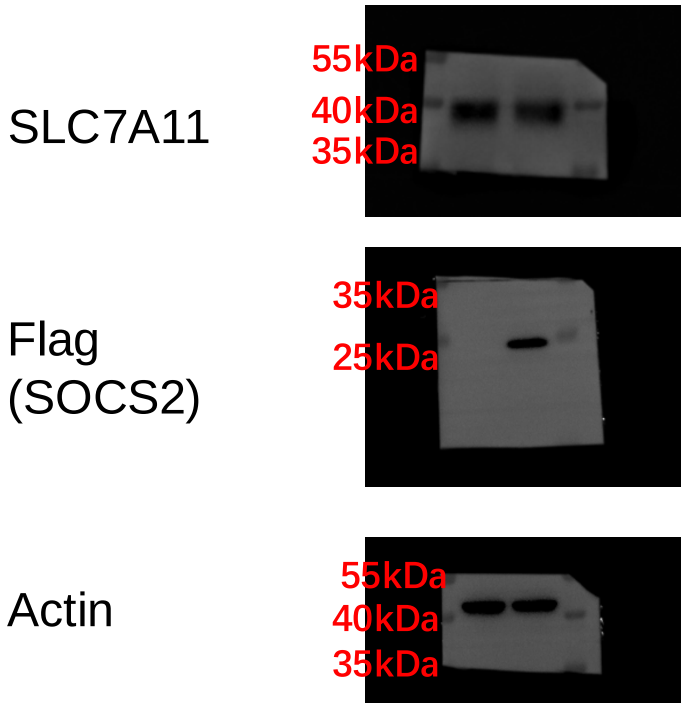
SLC7A11
55kDa
40kDa
35kDa
Flag
(SOCS2)
35kDa
25kDa
Actin
55kDa
40kDa
35kDa

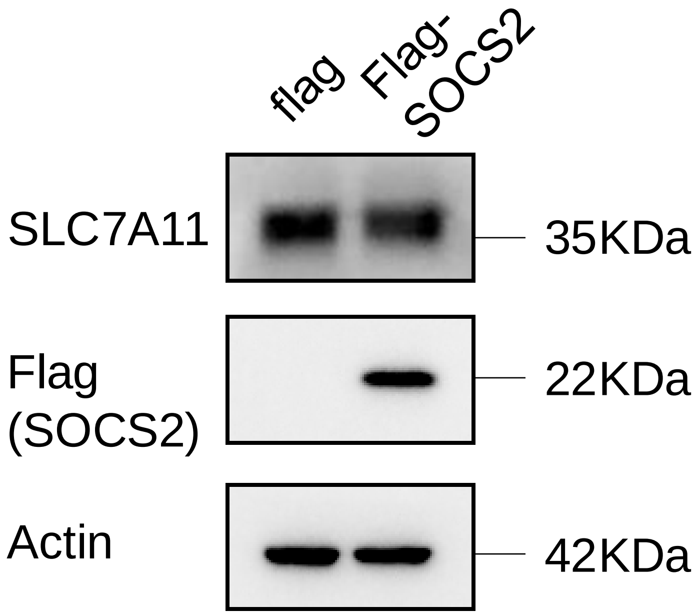
flag
Flag-
SOCS2
SLC7A11
35KDa
Flag
(SOCS2)
22KDa
Actin
42KDa

Fig 9J

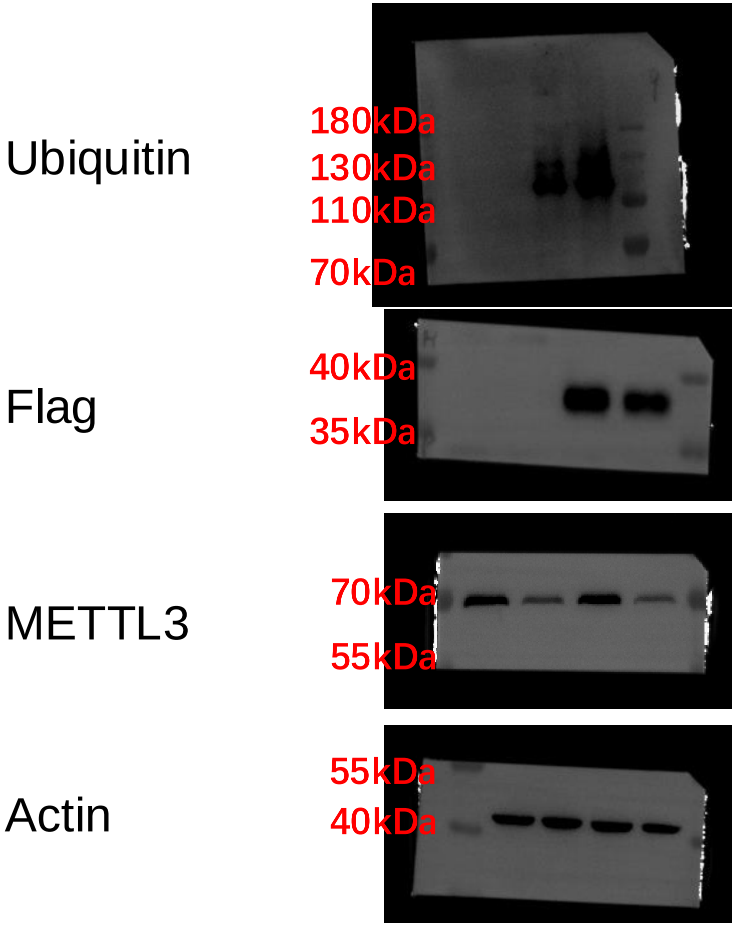
Ubiquitin
180kDa
130kDa
110kDa
70kDa
Flag
40kDa
35kDa
METTL3
70kDa
55kDa
Actin
55kDa
40kDa

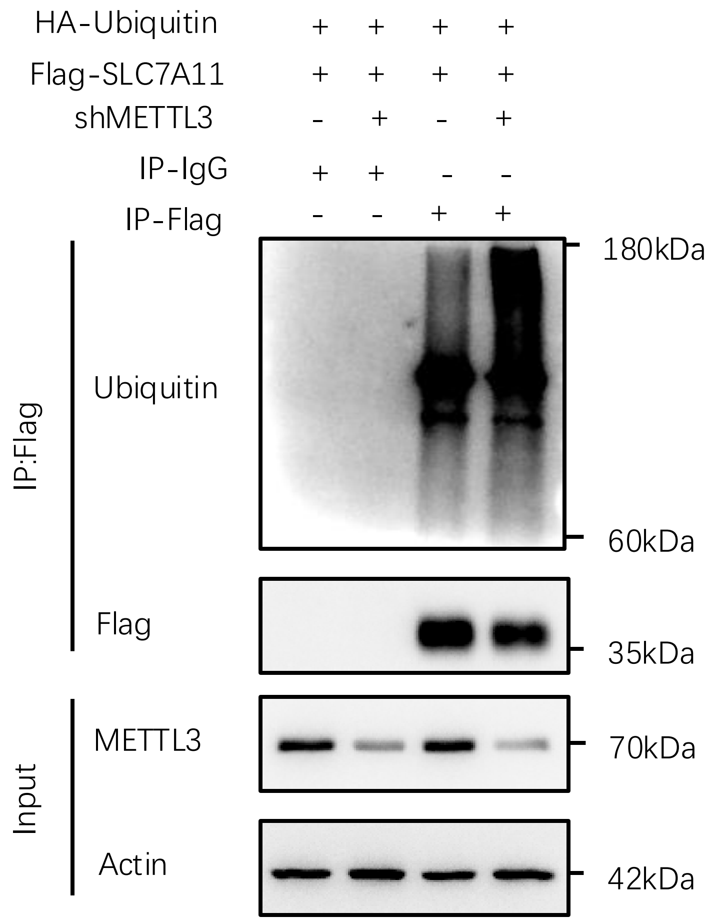
HA-Ubiquitin + + + +
Flag-SLC7A11 + + + +
shMETTL3 - + - +
IP-IgG + + - -
IP-Flag - - + +
IP:Flag
Ubiquitin
180kDa
60kDa
Flag
35kDa
Input
METTL3
70kDa
Actin
42kDa

Fig 9N

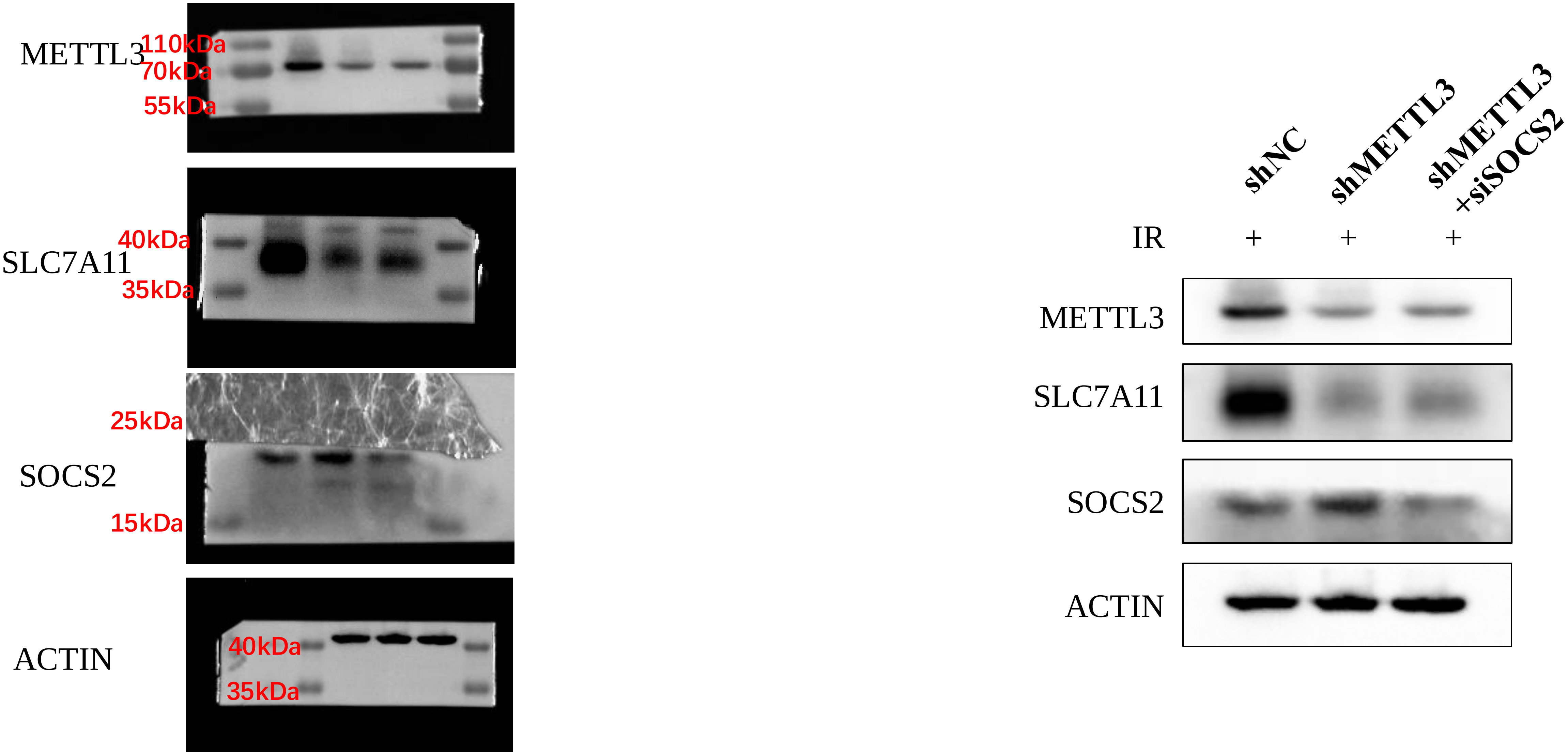
METTL3
110kDa
70kDa
55kDa
SLC7A11
40kDa
35kDa
SOCS2
25kDa
15kDa
ACTIN
40kDa
35kDa
shNC
shMETTL3
shMETTL3
+siSOCS2
IR
+
+
+
METTL3
SLC7A11
SOCS2
ACTIN

Figure S1A,B

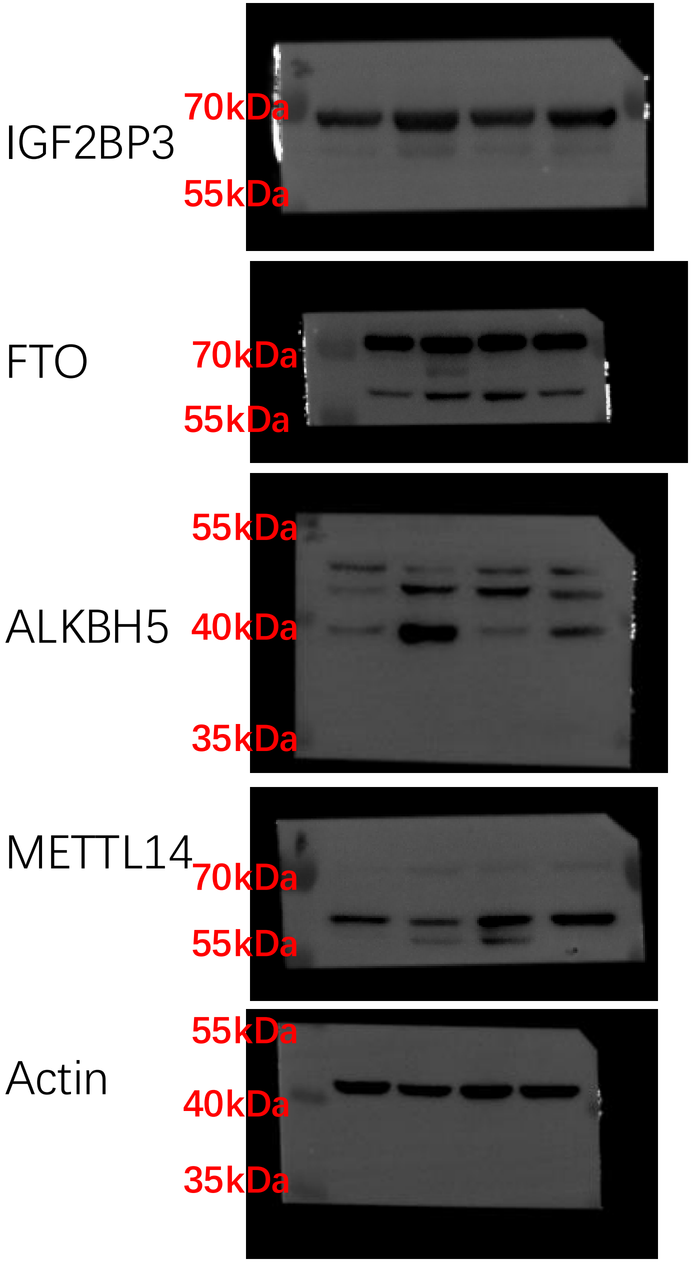
IGF2BP3
70kDa
55kDa
FTO
70kDa
55kDa
55kDa
ALKBH5
40kDa
35kDa
METTL14
70kDa
55kDa
55kDa
Actin
40kDa
35kDa

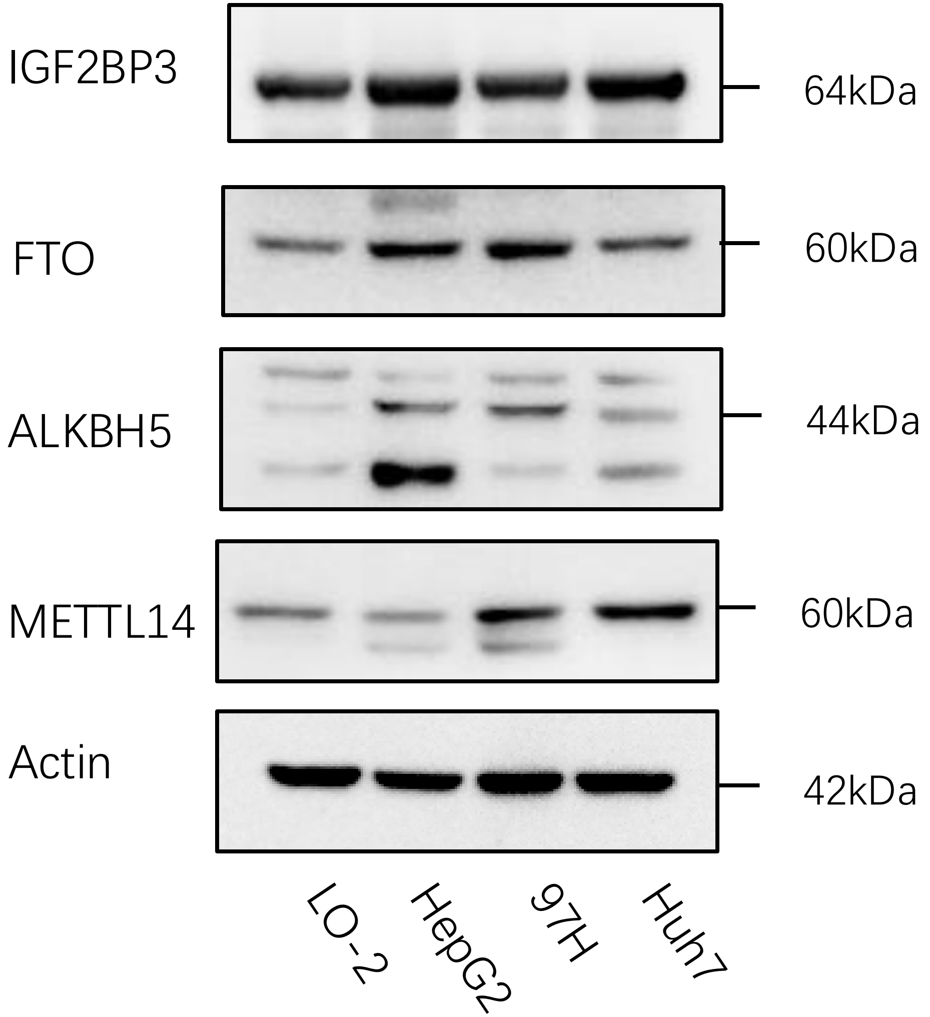
IGF2BP3
64kDa
FTO
60kDa
ALKBH5
44kDa
METTL14
60kDa
Actin
42kDa
LO-2
HepG2
97H
Huh7

Figure S1C

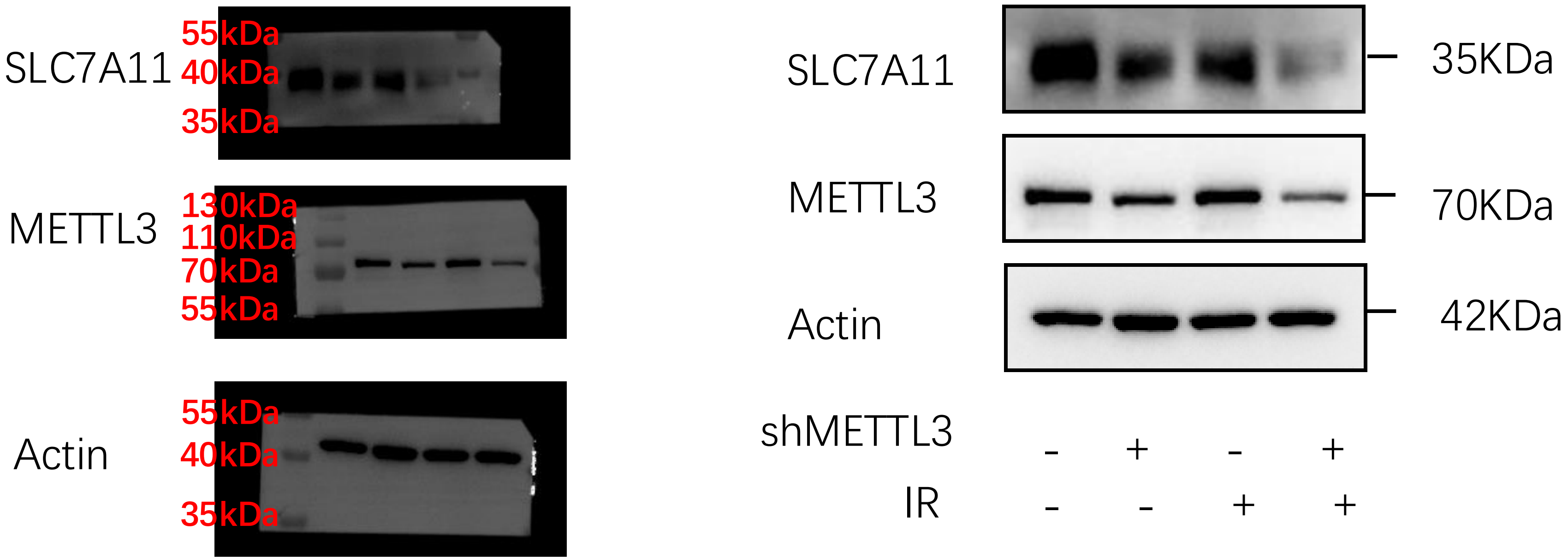
SLC7A11
55kDa
40kDa
35kDa
METTL3
130kDa
110kDa
70kDa
55kDa
Actin
55kDa
40kDa
35kDa
SLC7A11
35KDa
METTL3
70KDa
Actin
42KDa
shMETTL3
- + - +
IR
- - + +

Figure S1E

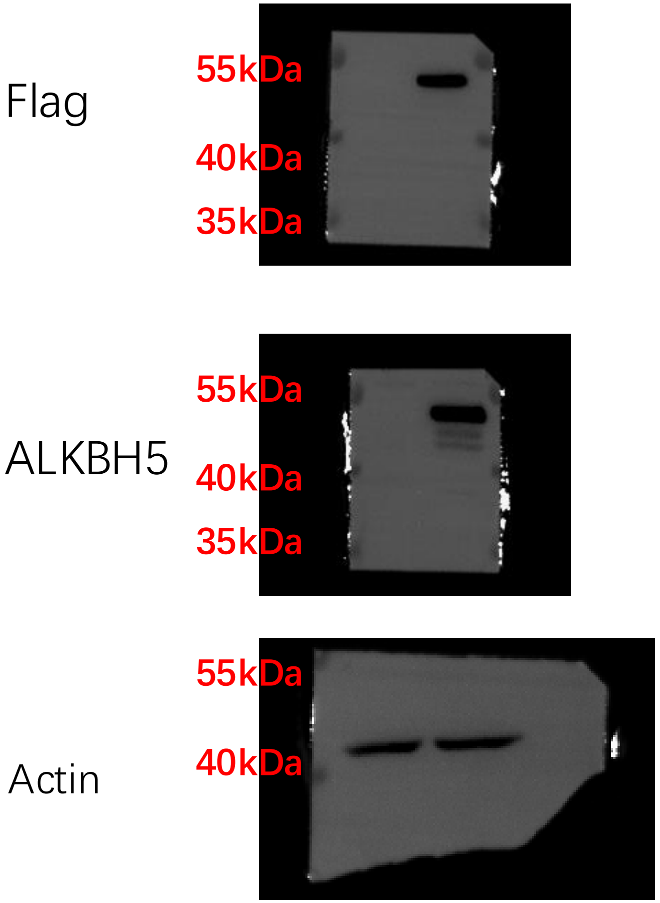
Flag
55kDa
40kDa
35kDa
ALKBH5
55kDa
40kDa
35kDa
Actin
55kDa
40kDa

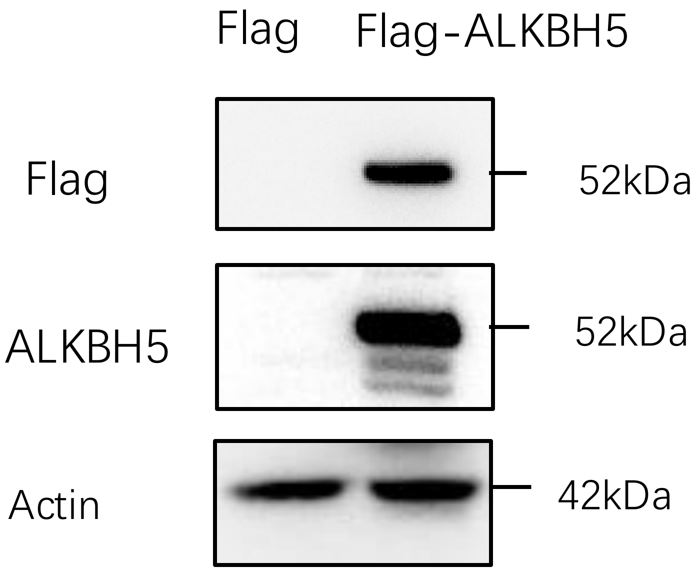
Flag
Flag-ALKBH5
Flag
52kDa
ALKBH5
52kDa
Actin
42kDa

Fig S1H

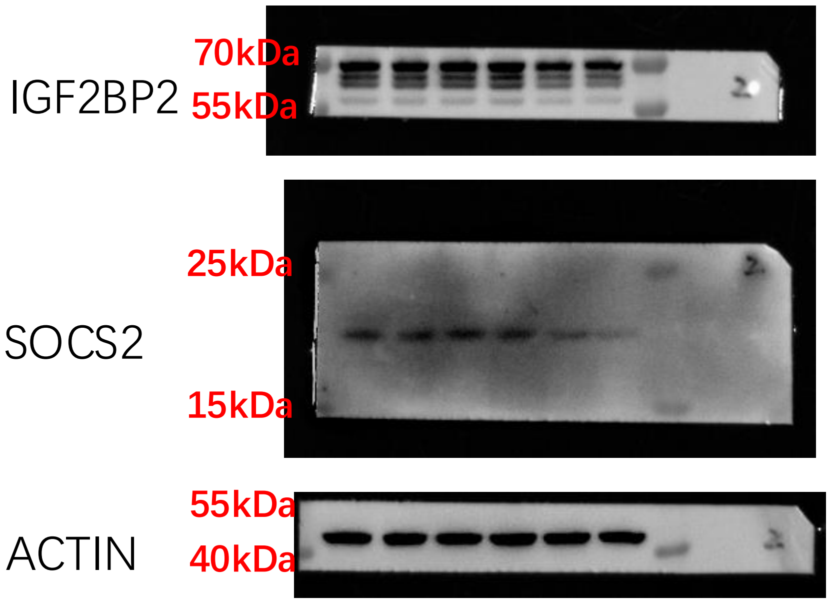
IGF2BP2
70kDa
55kDa
SOCS2
25kDa
15kDa
ACTIN
55kDa
40kDa

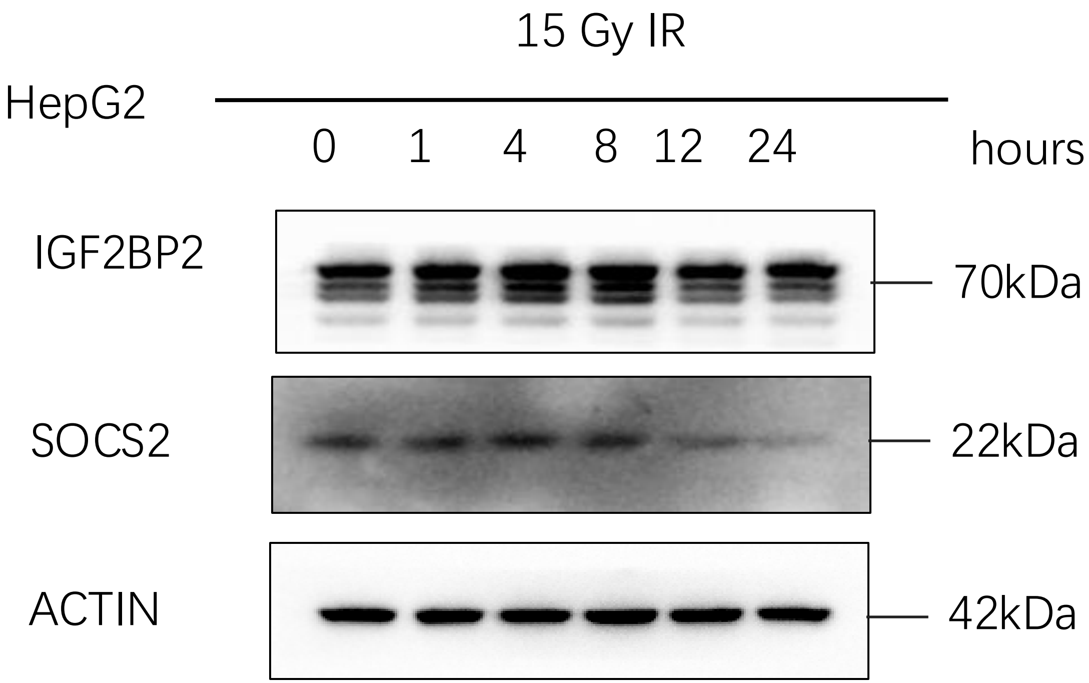
15 Gy IR
HepG2
0
1
4
8
12
24
hours
IGF2BP2
70kDa
SOCS2
22kDa
ACTIN
42kDa

Fig S1I

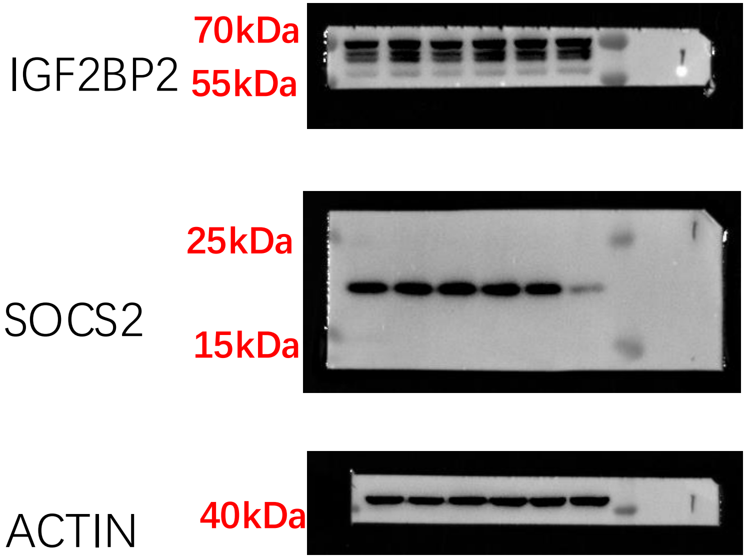
IGF2BP2
70kDa
55kDa
SOCS2
25kDa
15kDa
ACTIN
40kDa

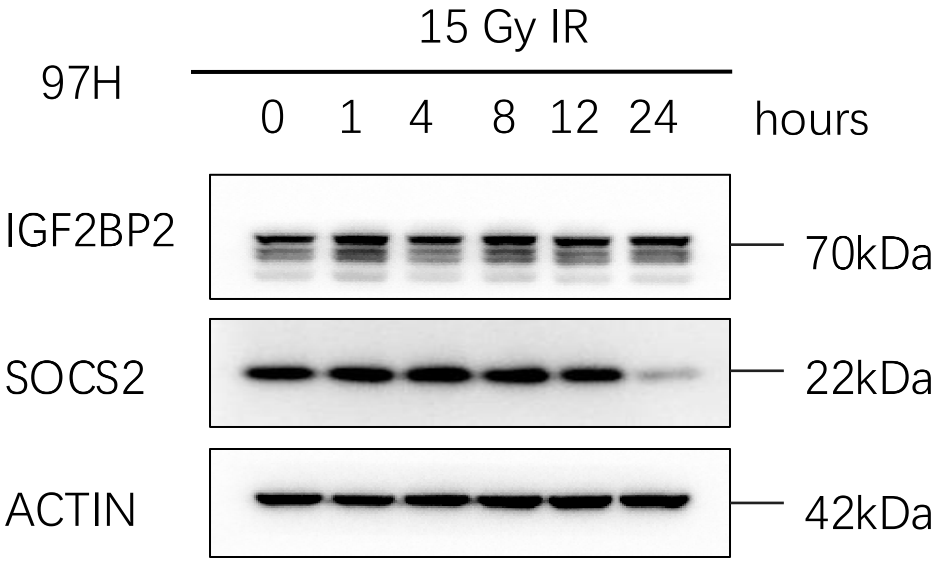
15 Gy IR
97H
0 1 4 8 12 24
hours
IGF2BP2
70kDa
SOCS2
22kDa
ACTIN
42kDa

Figure S3A

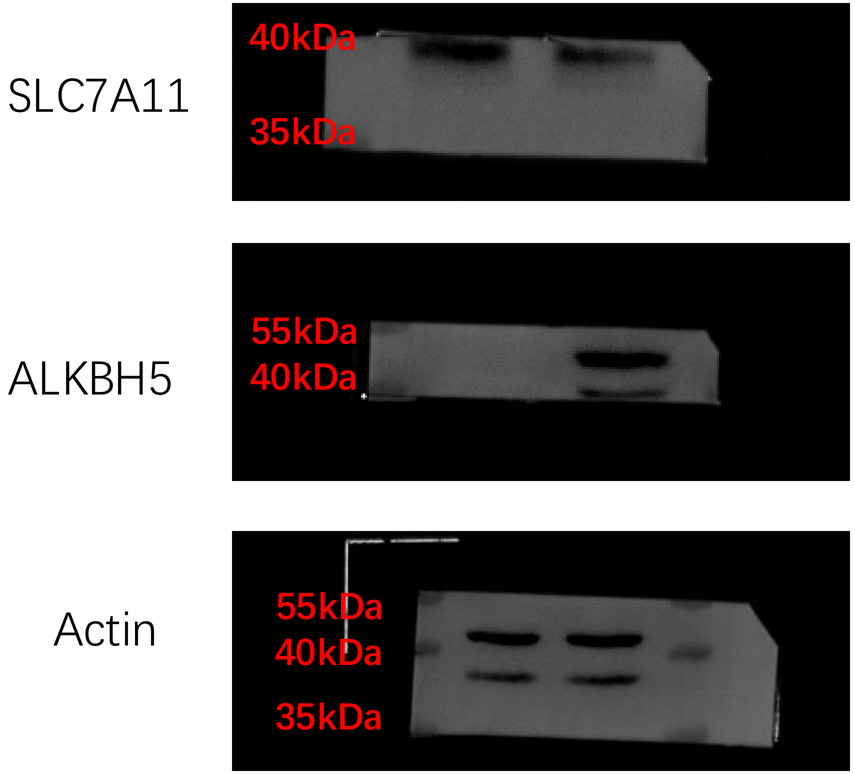
SLC7A11
40kDa
35kDa
ALKBH5
55kDa
40kDa
Actin
55kDa
40kDa
35kDa

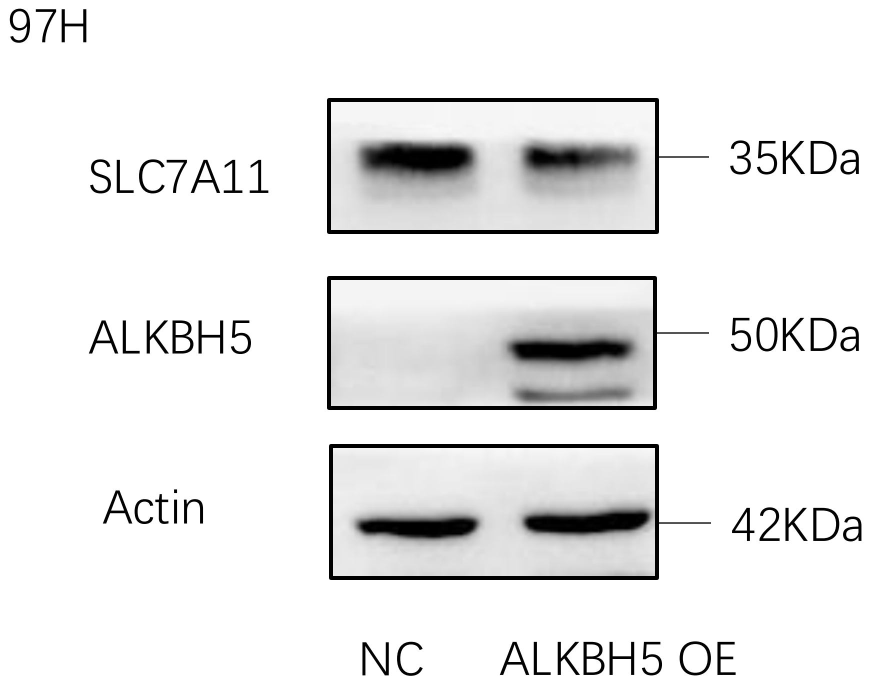
97H
SLC7A11
35KDa
ALKBH5
50KDa
Actin
42KDa
NC
ALKBH5 OE

Figure S3B

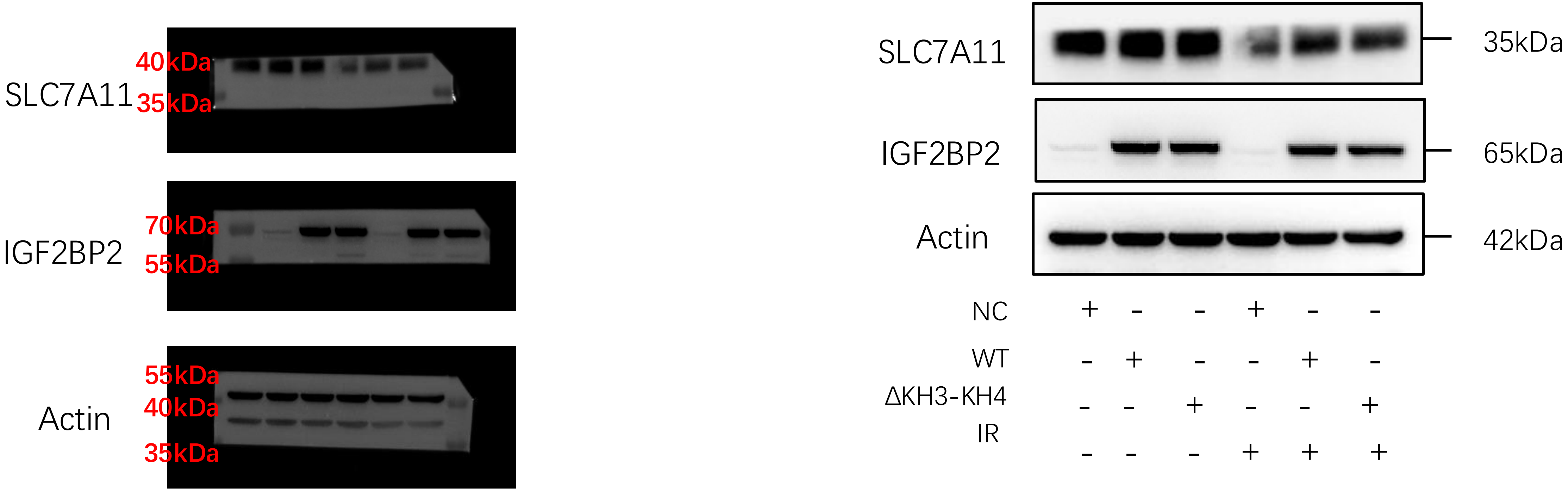
SLC7A11
40kDa
35kDa
IGF2BP2
70kDa
55kDa
Actin
55kDa
40kDa
35kDa
SLC7A11
35kDa
IGF2BP2
65kDa
Actin
42kDa
NC + - - + - -
WT - + - - + -
ΔKH3-KH4 - - + - - +
IR - - - + + +

Figure S3D

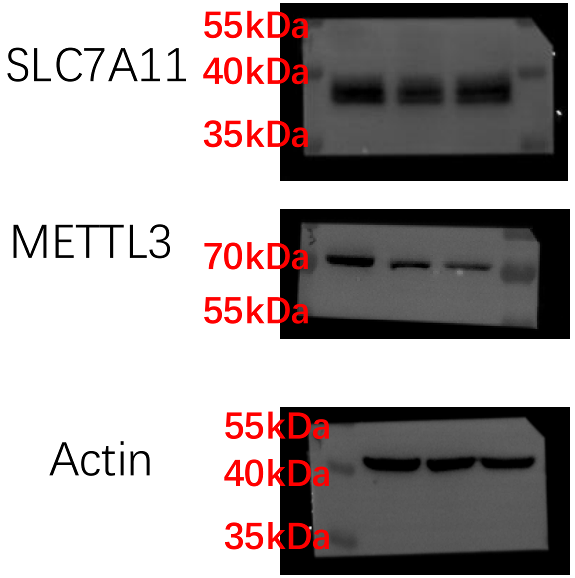

SLC7A11
55kDa
40kDa
35kDa
METTL3
70kDa
55kDa
Actin
55kDa
40kDa
35kDa

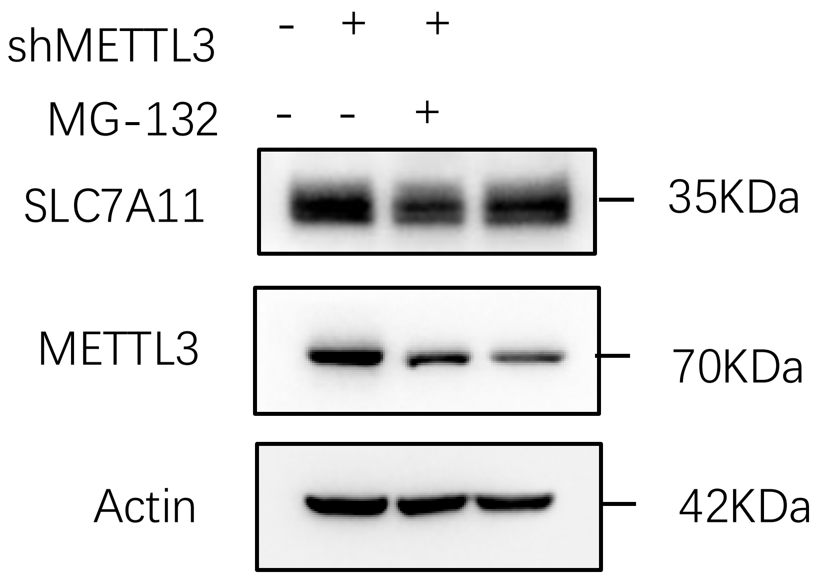

shMETTL3 - + +
MG-132 - - +
SLC7A11
35KDa
METTL3
70KDa
Actin
42KDa

Figure S3G

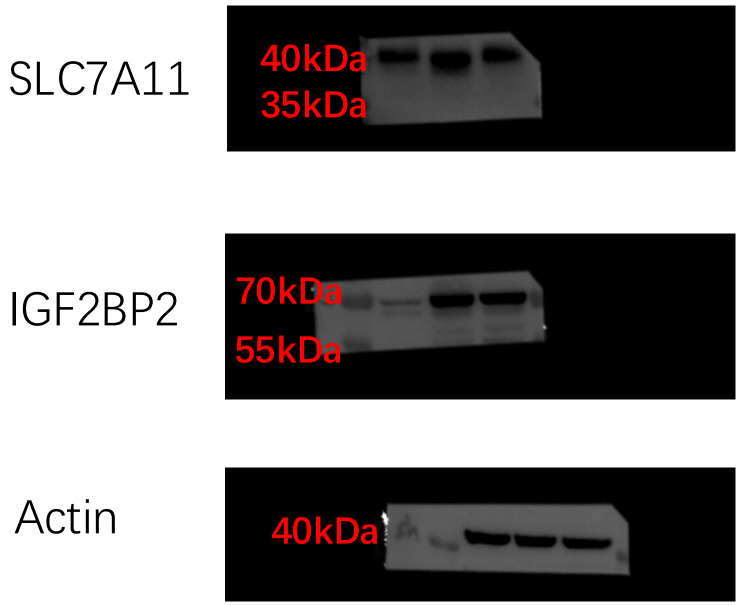

SLC7A11
40kDa
35kDa
IGF2BP2
70kDa
55kDa
Actin
40kDa

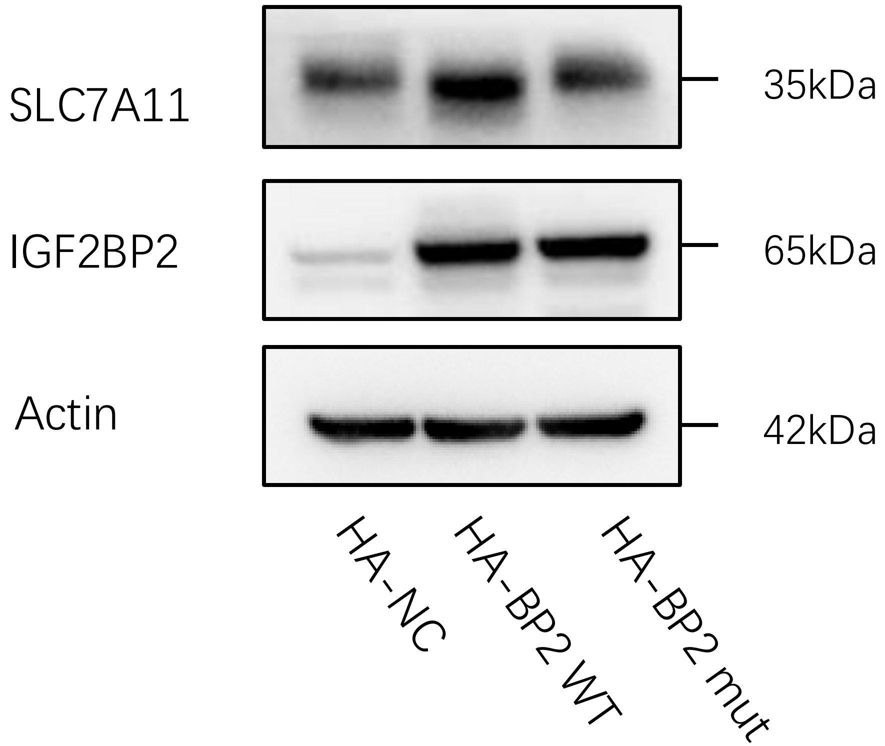

SLC7A11
35kDa
IGF2BP2
65kDa
Actin
42kDa
HA-NC
HA-BP2 WT
HA-BP2 mut

Figure S3I

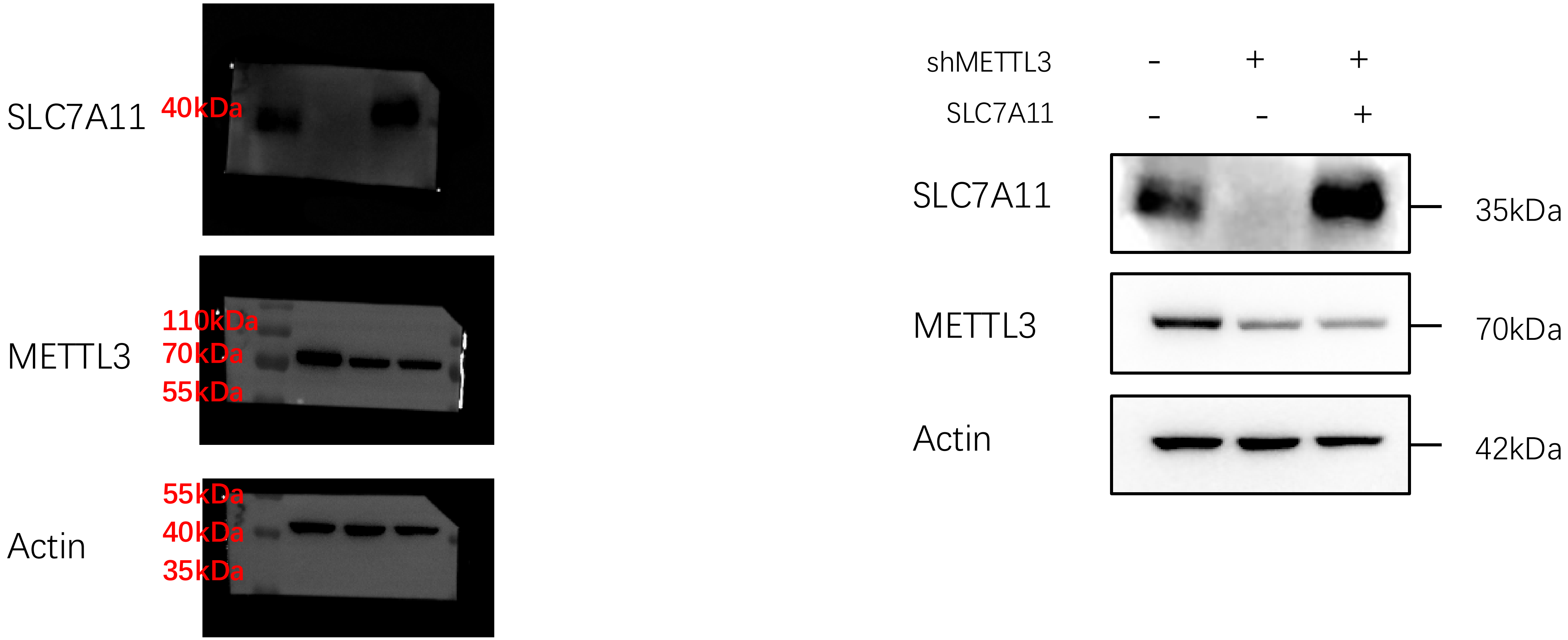

SLC7A11
40kDa
METTL3
110kDa
70kDa
55kDa
Actin
55kDa
40kDa
35kDa
shMETTL3 - + +
SLC7A11 - - +
SLC7A11
35kDa
METTL3
70kDa
Actin
42kDa

Figure S3L

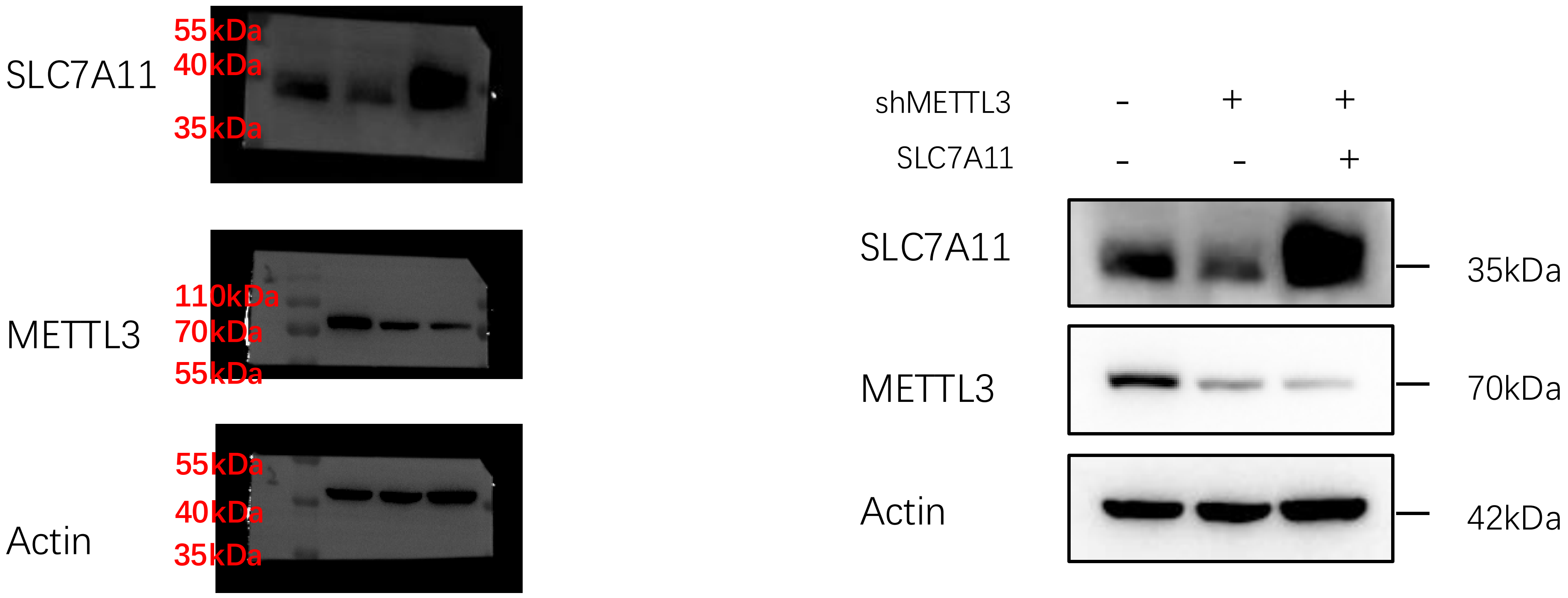
SLC7A11
55kDa
40kDa
35kDa
METTL3
110kDa
70kDa
55kDa
Actin
55kDa
40kDa
35kDa
shMETTL3 - + +
SLC7A11 - - +
SLC7A11
35kDa
METTL3
70kDa
Actin
42kDa
